# Synergies and trade-offs in the heat shock response mechanism

**DOI:** 10.1371/journal.pcbi.1014729

**Published:** 2026-09-02

**Authors:** Rupal Chauhan, Biswajit Das, Ajeet K. Sharma

**Affiliations:** 1 Department of Physics, Indian Institute of Technology Jammu, Jammu, India; 2 Amrita School of Artificial Intelligence, Amrita Vishwa Vidyapeetham, Coimbatore, India; 3 Department of Biosciences and Bioengineering, Indian Institute of Technology Jammu, Jammu, India; Christian Albrechts Universitat zu Kiel, GERMANY

## Abstract

*E. coli* relies on the heat shock response (HSR) to preserve protein homeostasis under stress, through three feedback modules: feedforward translational control, chaperone-mediated sequestration and targeted degradation. Although previous studies have highlighted how this layered architecture ensures rapid and robust protection compared to simpler designs, not much attention is paid to how these modules interact. Moreover, how do interactions among the three modules balance performance trade-offs, where gains in one module may come at the expense of another, yet together yield an optimal overall response? We address this using a mathematical model that integrates protein folding with $\sigma_{32}$ regulation. We show that the feedback modules both cooperate and compete, giving rise to nonmonotonic dynamics that govern HSR performance. Specifically, increasing feedforward strength does accelerate response, but beyond a threshold, despite increasing chaperone levels, it paradoxically slows recovery. Similarly, while sequestration enhances relative chaperone production and per-chaperone efficiency, when excessive, it traps $\sigma_{32}$ in inactive complexes, prolonging recovery and delaying shutdown. Mapping the parameter space reveals regimes of synergy as well as trade-offs between speed and efficiency, with wild-type parameters lying near the optimal region. These results reveal design principles that produces a robust and efficient heat shock response.

## Author summary

Proteins must adopt a specific three-dimensional structure to perform their function within the cell. However, they can misfold into non-functional states, especially under stress conditions such as elevated temperature. Misfolded proteins can accumulate and disrupt cellular processes, making their timely repair essential for cell survival. To manage this, cells employ the heat shock response, a conserved regulatory system that maintains protein homeostasis under stress. In *Escherichia coli*, this response is controlled by three interconnected modules: rapid production of the key regulator $\sigma_{32}$, its inactivation through chaperone binding, and its degradation. Together, these modules enable a rapid response while ensuring timely recovery once normal conditions are restored. We use a mathematical model to examine how these modules interact. Because they share common molecular components, particularly $\sigma_{32}$ and chaperones, and therefore do not act independently. Our analysis reveals that interactions between regulatory modules can be cooperative or competing, depending on the relative strength of the modules. Such interactions generate nonmonotonic behavior and trade-offs among recovery speed, efficient chaperone utilization, and timely shutdown of the stress response. We also identify an optimal operating regime in which the combined regulation balances competing demands, revealing general design principles of stress adaptation.

## Introduction

Proteins are indispensable biomolecules that perform a wide range of cellular functions, including catalyzing biochemical reactions [1,2], regulating gene expression [3], and providing structural support [4]. These functions depend on the acquisition of precise three-dimensional conformations [5], which are inherently vulnerable to disruption by environmental fluctuations [6], sequence-specific characteristics [5], and translation kinetics [7,8]. Misfolding events can produce conformations with diminished or abolished activity [8], often resulting in cytotoxicity and contributing to neurodegenerative diseases such as Alzheimer’s disease and systemic amyloidoses [9,10]. To protect proteome integrity, cells employ molecular chaperones, specialized proteins that facilitate correct folding and prevent aggregation [11–13]. Any failure in the chaperone system can severely compromise cellullar fitness and viability [14,15].

Cells are constantly exposed to environmental and physiological fluctuations that threaten protein stability [15,16]. Under stress conditions, such as elevated temperatures or altered pH levels, the likelihood of protein misfolding increases, potentially overwhelming the cellular chaperone machinery [6,17]. The resulting accumulation of misfolded proteins can disrupt essential biochemical pathways, form toxic aggregates, and ultimately lead to cell death [15]. To counteract such proteotoxic stress, cells activate the heat shock response (HSR), an evolutionarily conserved defense mechanism that induces the transient expression of heat shock proteins (HSPs) [18,19]. HSPs include molecular chaperones, which refold misfolded proteins, and proteases, which selectively degrade irreversibly damaged or aggregation-prone substrates [20]. Driven by heat shock factors that control the transcription of genes encoding HSPs [18,21], HSR is tightly regulated at both the transcriptional and translational levels, ensuring a rapid and efficient restoration of protein homeostasis [21,22].

An effective heat shock response must achieve a delicate balance between speed and efficiency, ensuring that cells mount a timely defense against proteotoxic stress while avoiding unnecessary metabolic expenditure [23,24]. In *Escherichia coli*, this protective system is governed by the alternative sigma factor $\sigma_{32}$ (RpoH), which directs the transcription of heat shock proteins, mainly molecular chaperones and proteases [25,26]. The activity of $\sigma_{32}$ is tightly regulated through three interconnected control modules. First, a feedforward translation control mechanism rapidly increases $\sigma_{32}$ synthesis upon a rise in temperature, enabling immediate activation of stress-protective genes [27]. Second, chaperone-mediated sequestration, primarily through DnaK / DnaJ and GroEL / GroES, transiently binds to $\sigma_{32}$, modulating its availability according to the demand for folding assistance [28,29]. Third, FtsH-dependent proteolytic degradation ensures that excess $\sigma_{32}$ is removed once homeostasis is restored, preventing wasteful overproduction of chaperones [30,31]. These regulatory layers act in concert to match chaperone synthesis both proportionally and temporally to the severity of stress, while facilitating a rapid return to baseline activity when protein homeostasis is restored [28,29,32,33]. This interplay of feedback not only protects cell fitness during acute heat stress, but also optimizes resource allocation for long-term survival [34–36].

Each of the three modules in the *E. coli* heat shock response is well characterized [20,24,37–39]. Previous studies have focused primarily on how these three feedback controls work cooperatively to deliver a rapid and robust response to protein misfolding [23,24,34,36]. Early modeling by Kurata et al. [36] studied the dynamic behavior of HSR and highlighted the evolutionary advantage of combining feedforward and feedback control over simpler strategies. It was later extended by El-Samad et al. [23], who utilized control theory framework and showed that the fast response to heat stress, noise rejection, and robustness are emergent properties of the HSR’s complex and modular regulatory architecture. Building on this, Kurata et al. [24] demonstrated that removing feedback limits the kinetic range in which the HSR can function efficiently. Together, these studies justify the existence of specific design motifs in terms of efficiency, and sensitivity, with the HSR architecture representing an evolutionarily selected robust solution.

Despite these insights, previous analyses have primarily examined how individual regulatory modules contribute to overall HSR performance and have not fully explored the interactions between these modules when they operate simultaneously. Because feedforward translation, chaperone-mediated sequestration, and degradation modules all act on overlapping pools of $\sigma_{32}$ and chaperones, strengthening one pathway to improve a functional aspect of the HSR (e.g., response speed) can interfere with the other. In this study, we investigate whether the combined action of these modules universally leads to a cooperative enhancement of performance or whether their mechanistic interaction can instead generate competing dynamics and nonmonotonic behavior. Competing interactions can also lead to optimal operating regimes, where response speed and efficiency are balanced under the constraints imposed by these modules. For example, the feedforward translation module enables a quick response by increasing $\sigma_{32}$ synthesis. At moderate levels, this can accelerate recovery by boosting chaperone production. However, excessive $\sigma_{32}$ may trigger premature sequestration of chaperones even when misfolded proteins are still abundant, slowing the overall response. Such competing dynamics can also give rise to trade-offs between the efficient use of chaperone machinery and the overall response time of the HSR. Therefore, we investigate how mechanistic coupling of HSR modules shapes overall performance and whether optimal function arises from maximizing individual module strength or from balancing interacting feedback pathways within a constrained regulatory architecture.

We address these questions using a mathematical model of the *E. coli* heat shock response that integrates the protein folding cycle with regulation of the heat shock sigma factor. Compared to simpler regulatory configurations, the layered architecture of the full HSR enables rapid responses and minimizes protein misfolding while preserving chaperone efficiency. While these observations agree with previous studies, which largely focused on cooperative interactions, our analysis reveals that such optimality emerges only within specific regimes of operation. We further show that the three feedback modules exhibit both cooperation and competition, giving rise to nonmonotonic behaviors and trade-offs between response speed and chaperone efficiency. These interactions create distinct HSR regimes, where feedback modules either act synergistically to boost overall performance or compete, where benefits from one module limit the performance of another. For example, at low and high feedforward strengths, the HSR exhibits two such regimes: a synergistic regime, where response speed and chaperone efficiency improve together, and a trade-off regime, where faster responses come at the expense of efficiency. The wild-type system lies within the trade-off regime, suggesting evolutionary tuning for rapid recovery while limiting the metabolic cost of excessive feedforward activity. Similarly, while stronger sequestration improves individual chaperone efficiency, it can slow the overall response. Finally, we identify an optimal operating regime in which the combined action of all feedbacks overcomes individual limitations, enabling faster responses and efficient chaperone use. Our analysis reveals that the HSR prioritizes a balance between response speed, chaperone efficiency, and recovery, rather than optimizing any single metric. This strategy reflects a design principle that likely shaped the evolution of bacterial stress resilience.

## Method

### Model of HSR in *E. coli*

**Fig 1:**
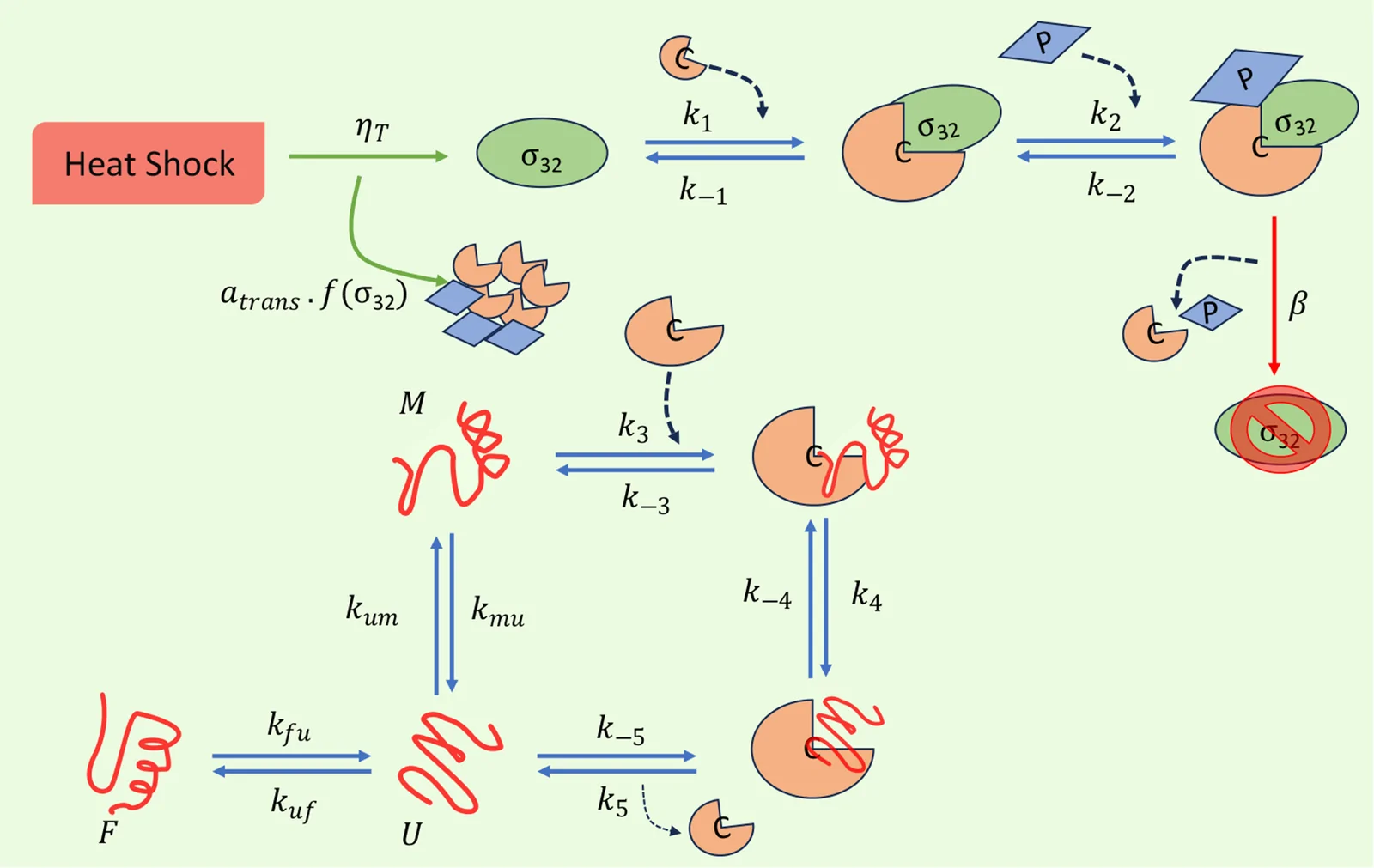
A coarse-grained schematic of the heat shock response model in *Escherichia coli.* The model includes three regulatory mechanisms: feedforward translational control, chaperone-mediated sequestration, and proteolytic degradation. Detailed descriptions of these mechanisms are provided in the Methods section, and the corresponding rate constants for each kinetic step are listed in Table 1.

We built a simplified mathematical model of the heat shock response in *E. coli*, capturing the essential features and dynamics of the system (Fig 1). The model includes: transcription factor ($\sigma_{32}$), chaperones (C), proteases (P), and protein substrates. The proteins in our model can exist in three different states, *i.e.*, misfolded (M), unfolded (U), and folded (F). The transitions between these states are governed by four kinetic parameters: folding ($k_{uf}$, U → F) and unfolding ($k_{fu}$, F → U), as well as misfolding ($k_{um}$, U → M) and rescue to the unfolded state ($k_{mu}$, M → U) of the protein. We assume that chaperones bind only to the misfolded state of the protein, forming C·M complex at a rate of *k*_3_, and the reverse step occurs at a rate of *k*_-3_. Upon binding to misfolded proteins, chaperones undergo conformational changes driven by ATP hydrolysis that trigger the unfolding of the misfolded protein [40,41]. This led to the formation of C·U complex with a forward rate *k*_4_ and the backward step occurs at a rate *k*_-4_. The unfolded protein then dissociates at rate *k*_5_, and the reverse reaction occurs at rate *k*_-5_. Once released, these unfolded proteins can refold into their native conformation.

The production of chaperones and proteases in our model is controlled by $\sigma_{32}$, whose activity is regulated by three interconnected regulatory layers, sequestration, degradation, and feedforward control [20,27,38,42]. Under non-stress conditions, the activity of $\sigma_{32}$ is suppressed via sequestration by chaperones (C) [32,43]. $\sigma_{32}$ binds with chaperones at a forward rate *k*_1_, forming an inactive $\sigma_{32}\cdot C$ complex. The backward step occurs at rate *k*_-1_. This complex prevents $\sigma_{32}$ from binding to RNA polymerase (RNAP), thereby repressing the transcription of heat shock genes [37]. Sequestration of sigma factor also facilitates the degradation loop, in which the $\sigma_{32}\cdot C$ complex recruits proteases (forming $\sigma_{32}\cdot C\cdot P$) at rate *k*_2_ with reverse rate *k*_-2_, leading to degradation of $\sigma_{32}$ at rate β [30–32]. Under optimal growth conditions, the combined actions of sequestration by chaperones and degradation by proteases limit the accumulation of $\sigma_{32}$, thus maintaining it at low basal levels [20,21].

Heat shock disrupts the steady-state balance by greatly increasing the proportion of misfolded proteins, which promotes the dissociation of chaperones from $\sigma_{32}$ to facilitate refolding of these substrates [44,45]. As a result, $\sigma_{32}$ is released from $\sigma_{32}\cdot C$ complexes, stabilizes, and binds to RNA polymerase to initiate heat shock gene transcription [44,46]. At the same time, a feedforward temperature-sensing mechanism enhances $\sigma_{32}$ synthesis [27]. Under normal conditions, secondary structures in the rpoH mRNA fold in a way that hides the ribosome binding site, preventing translation initiation [46]. However, during heat shock, these secondary structures are destabilized, exposing the ribosome binding site and allowing translation to proceed, which increases the translation of $\sigma_{32}$ with the rate $\eta_{T}$ [44,46].

While the model adopts a simplified representation of protein folding and chaperone classes, it retains all essential regulatory molecules and processes governing $\sigma_{32}$ activity, including feedforward synthesis, chaperone-mediated sequestration and protease-mediated degradation; these constitute the core focus of this study.

### Model equations

We investigate the dynamics of the heat shock response, described in Fig 1 by solving ten coupled ordinary differential equations (ODEs) with three mass-balance constraints. We denote the total concentration of a molecular species *X* by $[X{]}_{t}$, while its free (unbound) concentration is denoted by $[X{]}_{f}$. The governing equations are expressed as follows:


$$\begin{array}{ccc} \frac{d[\sigma_{32}{]}_{t}}{dt} & = & \eta_{T}-\beta [\sigma_{32}.C.P]-\beta_{dil}[\sigma_{32}{]}_{t} \end{array}$$ (1)

$$\begin{array}{ccc} \frac{d[\sigma_{32}.C]}{dt} & = & k_{1}[C{]}_{f}[\sigma_{32}{]}_{f}-k_{-1}[\sigma_{32}.C]+k_{-2}[\sigma_{32}.C.P]-k_{2}[P{]}_{f}[\sigma_{32}.C] \end{array}$$ (2)

$$\begin{array}{ccc} \frac{d[\sigma_{32}.C.P]}{dt} & = & k_{2}[P{]}_{f}[\sigma_{32}.C]-k_{-2}[\sigma_{32}.C.P]-\beta [\sigma_{32}.C.P] \end{array}$$ (3)

$$\begin{array}{ccc} \frac{d[M]}{dt} & = & k_{-3}[C.M]-k_{3}[C{]}_{f}[M]+k_{um}[U]-k_{mu}[M] \end{array}$$ (4)

$$\begin{array}{ccc} \frac{d[C.M]}{dt} & = & k_{3}[C{]}_{f}[M]-k_{-3}[C.M]+k_{-4}[C.U]-k_{4}[C.M] \end{array}$$ (5)

$$\begin{array}{ccc} \frac{d[C.U]}{dt} & = & k_{4}[C.M]-k_{-4}[C.U]+k_{-5}[C{]}_{f}[U]-k_{5}[C.U] \end{array}$$ (6)

$$\begin{array}{ccc} \frac{d[U]}{dt} & = & k_{mu}[M]-k_{um}[U]+k_{fu}[F]-k_{uf}[U]+k_{5}[C.U]-k_{-5}[C{]}_{f}[U] \end{array}$$ (7)

$$\begin{array}{ccc} \frac{d[F]}{dt} & = & k_{uf}[U]-k_{fu}[F] \end{array}$$ (8)

$$\begin{array}{ccc} \frac{d[C{]}_{t}}{dt} & = & a_{trans}\frac{[\sigma_{32}{]}_{f}}{K_{\sigma }+[\sigma_{32}{]}_{f}}-\beta_{dil}[C{]}_{t} \end{array}$$ (9)

$$\begin{array}{ccc} \frac{d[P{]}_{t}}{dt} & = & a_{trans}\frac{[\sigma_{32}{]}_{f}}{K_{\sigma }+[\sigma_{32}{]}_{f}}-\beta_{dil}[P{]}_{t} \end{array}$$ (10)

$$\begin{array}{ccc} {}[\sigma_{32}{]}_{t} & = & \left[\sigma_{32}{]}_{f}+[\sigma_{32}.C]+[\sigma_{32}.C.P\right] \end{array}$$ (11)

$$\begin{array}{ccc} {}[C{]}_{t} & = & \left[C{]}_{f}+[\sigma_{32}.C]+[\sigma_{32}.C.P]+[C.M]+[C.U\right] \end{array}$$ (12)

$$\begin{array}{ccc} {}[P{]}_{t} & = & \left[P{]}_{f}+[\sigma_{32}.C.P\right] \end{array}$$ (13)

The synthesis of chaperones (Eq. 9) and proteases (Eq. 10) is transcriptionally activated by free $\sigma_{32}$. This activation is represented using a Michaelis-Menten-like formulation, where $K_{\sigma }$ denotes the dissociation constant that characterizes the affinity between $\sigma_{32}$ and the transcriptional machinery, and *a*_trans_ specifies the maximum achievable production rate under saturating concentrations of $\sigma_{32}$. The mass balance relationships are described in Eqs. (11)-(13), which account for the total concentrations of $\sigma_{32}$, chaperones (C), and proteases (P). The complete set of rate constants and parameter values used in the model is listed in Table 1.

**Table 1:** Parameters of *E. coli* heat shock mechanism used in the model.

S. No.	Parameter	Value	References
1	$\eta_{T}$	0.075, 0.3 μM min^-1^	*
2	*k*_1_	19.8 $\mu {\text{M}}^{-1}$ min^-1^	[32]
3	*k*_-1_	0.372 min^-1^	[32]
4	*k*_2_	20 $\mu {\text{M}}^{-1}$ min^-1^	Similar to *k*_1_ [32]
5	*k*_-2_	1.998 min^-1^	*[47]
6	*k*_3_	59.4 $\mu {\text{M}}^{-1}$ min^-1^	[48,49]
7	*k*_-3_	0.372 min^-1^	[48,49]
8	*k*_4_	108 min^-1^	estimated from [50]
9	*k*_-4_	1.08 min^-1^	estimated^*a*^
10	*k*_5_	120 min^-1^	[51]
11	*k*_-5_	1.2 min^-1^	[52]
12	β	3 min^-1^	[21,23]
13	$k_{mu}$	0.12 min^-1^	[53]^*b*^
14	$k_{um}$	75, 150 min^-1^	[53]^*b*^
15	$k_{fu}$	0.48 min^-1^	[53]^*b*^
16	$k_{uf}$	750 min^-1^	[53]^*b*^
17	$a_{trans}$	2 min^-1^	[54]
		0.2 min^-1^ (for protease)	[54]
18	$K_{\sigma }$	0.05 μM	[55]
19	$\beta_{dil}$	0.03 min^-1^	[56,57]

Rate constants were taken from published experimental studies of the heat shock response wherever available. In cases where direct measurements were not available, parameters were chosen within reported physiological ranges. ^*^As $\sigma_{32}$ is present in low concentrations [21,32,58], the translation rate $\eta_{T}$ was chosen to reproduce steady-state concentrations in the experimentally observed few hundred nanomolar range, following the approach in Ref. [55]. The dissociation rate *k*_-2_ was calculated such that $\frac{k_{2}}{k_{-2}}=10^{7}M^{-1}$, consistent with Refs. [23,47]. ^*a*^Due to unavailability of a specific value for *k*_-4_, it was estimated such that $\frac{k_{4}}{k_{-4}}=100$, reflecting the experimentally supported forward bias of ATP-driven unfolding reactions [40]. ^*b*^Protein folding rates were chosen to be comparable to those of malate dehydrogenase protein [53]. See S1 Text for more details on model calibration and validation.

### Numerical dissection of the *E. coli* heat shock response mechanism

To quantify the contribution of each regulatory mechanism, we developed three model variants derived from the full HSR network. The first variant, termed the *feedforward-only*, retained the transcriptional activation of chaperone production by $\sigma_{32}$, but excluded both sequestration (by setting $k_{1}=k_{-1}=0$) and targeted degradation ($k_{2}=k_{-2}=\beta =0$). The second variant incorporated both feedforward activation and sequestration of $\sigma_{32}$ but omitted targeted degradation. Finally, the *full HSR* model included all three layers, representing the entire regulatory network. To ensure comparable folding levels before heat shock, we calibrated the pre-heat shock steady-state concentrations of $\sigma_{32}$ and chaperones in each reduced versions to match those of the full HSR model. It was achieved by tuning the translation rates of sigma factor ($\eta_{T}$) and chaperones ($a_{trans},K_{\sigma }$). This approach, based on established methodologies [23,24,59], sets a consistent baseline, allowing us to isolate the effects of each regulatory mechanism.

For a systematic evaluation of the cellular response and the efficiency of chaperone action, we considered several quantitative performance metrics. The first metric is *folding yield*, denoted by $\eta_{y}$, which quantifies the fraction of folded proteins [*F*] relative to the total protein concentration [*T*]. It is mathematically expressed as


$$\begin{array}{c} \eta_{y}=\frac{\left[F\right]}{\left[T\right]}. \end{array}$$ (14)

By definition, $\eta_{y}$ ranges from 0 to 1, with higher values corresponding to a higher proportion of correctly folded proteins [52]. The second metric is *benefit-to-cost ratio* ($\eta_{bc}$), which quantifies the increase in functional (folded) proteins attributable to chaperone activity relative to the total number of chaperones. It is defined as


$$\begin{array}{c} \eta_{bc}=\frac{[F{]}_{c}-[F{]}_{nc}}{\left[C\right]}, \end{array}$$ (15)

where $[F{]}_{c}$ and $[F{]}_{nc}$ represent the concentrations of folded proteins in the presence and absence of chaperones, respectively. [*C*] denotes the total concentration of chaperones. This formulation measures the number of folded proteins gained per chaperone molecule.

We also define response time as the time required for the concentration of folded proteins to recover to 97% of their preheat shock steady-state value. This threshold was chosen to represent the point at which protein homeostasis is effectively restored, leaving only a minimal deviation from the pre-stress condition. Finally, we define *performance index* denoted by $\eta_{p}$, a qualitative metric that integrates both the efficiency (benefit-to-cost, $\eta_{bc}$) and the speed (response time, RT) of the heat shock response. It is given by


$$\begin{array}{c} \eta_{p}=\frac{\eta_{bc}({\text{RT}}^{max}-\text{RT})}{\eta_{bc}^{max}{\text{RT}}^{max}}. \end{array}$$ (16)

Here, ${\text{RT}}^{max}$ and $\eta_{bc}^{max}$ denote the maximum response time and the maximum benefit-to-cost ratio, respectively, observed across all combination of parameters considered in this study. This product of normalized response time and benefit-to-cost ratio ensures that $\eta_{p}$ ranges from 0 to 1. A higher performance index corresponds to faster and more efficient recovery from heat-induced protein misfolding.

In addition, to fully capture the response profile, we define adaptation time as the total duration required for the cell to activate its protective response and subsequently down-regulate to a new steady state as homeostasis is restored. Longer adaptation times may reflect prolonged high chaperone production, which can delay growth and other essential cellular processes.

### Extension of the model to include ATP dependence

To examine how cellular energy availability influences the heat shock response, we extended the model by introducing Michaelis-Menten type dependence [60–62] in two key processes (i) protease-mediated $\sigma_{32}$ degradation (β) [30,31] and (ii) chaperone-mediated refolding of misfolded proteins on ATP concentration (*k*_4_) [40,41].

Specifically, the corresponding rate constants were scaled as follows:


$$\begin{array}{c} \beta^{\prime }=\beta \frac{\left[\text{ATP}\right]}{K_{\text{ATP}}+[\text{ATP}]}\;\text{and}\;k_{4}^{\prime }=k_{4}\frac{\left[\text{ATP}\right]}{K_{\text{ATP}}+[\text{ATP}]}, \end{array}$$

where, β and *k*_4_ denote the maximum degradation rate and chaperone-assisted protein folding rate, respectively. *K*_ATP_ is the Michaelis constant that is the ATP concentration at which we get half of the maximal rate. This extension introduces ATP dependence only at the level of specific regulatory reactions (chaperone-assisted refolding and $\sigma_{32}$ degradation). While ATP availability may influence broader aspects of cellular physiology, modeling full metabolic coupling lies beyond the scope of the present study.

ATP concentration was defined as a normalized dimensionless variable A ∈ [0,1],


$$\begin{array}{c} \text{A}=\frac{\left[\text{ATP}\right]}{[\text{ATP}{]}_{\text{max}}}, \end{array}$$

where [ATP]_max_ represents a reference ATP concentration corresponding to saturating cellular ATP levels. This normalization allows ATP availability to be varied systematically while keeping the functional form of ATP-dependent reaction kinetics unchanged. Here, A = 0 corresponds to strongly ATP-limited conditions, while A = 1 represents high ATP availability under which ATP-dependent reactions approach their maximal rates.

The effective degradation rate and folding rate constant were modified as:


$$\begin{array}{c} \beta^{\prime }=\beta \frac{\text{A}}{K_{\text{A}}+\text{A}}\;\text{and}\;k_{4}^{\prime }=k_{4}\frac{\text{A}}{K_{\text{A}}+\text{A}}, \end{array}$$

Here, *K*_A_ is a dimensionless half-saturation parameter ($K_{\text{A}}=K_{\text{ATP}}/[\text{ATP}{]}_{\text{max}}$) that determines the sensitivity of these reactions to ATP availability. Smaller values of *K*_A_ correspond to higher ATP affinity, such that saturation is achieved at lower ATP levels, whereas larger values indicates weaker dependence on ATP.

## Result

### A Trade-off between Response Time and Benefit-to-Cost

**Fig 2:**
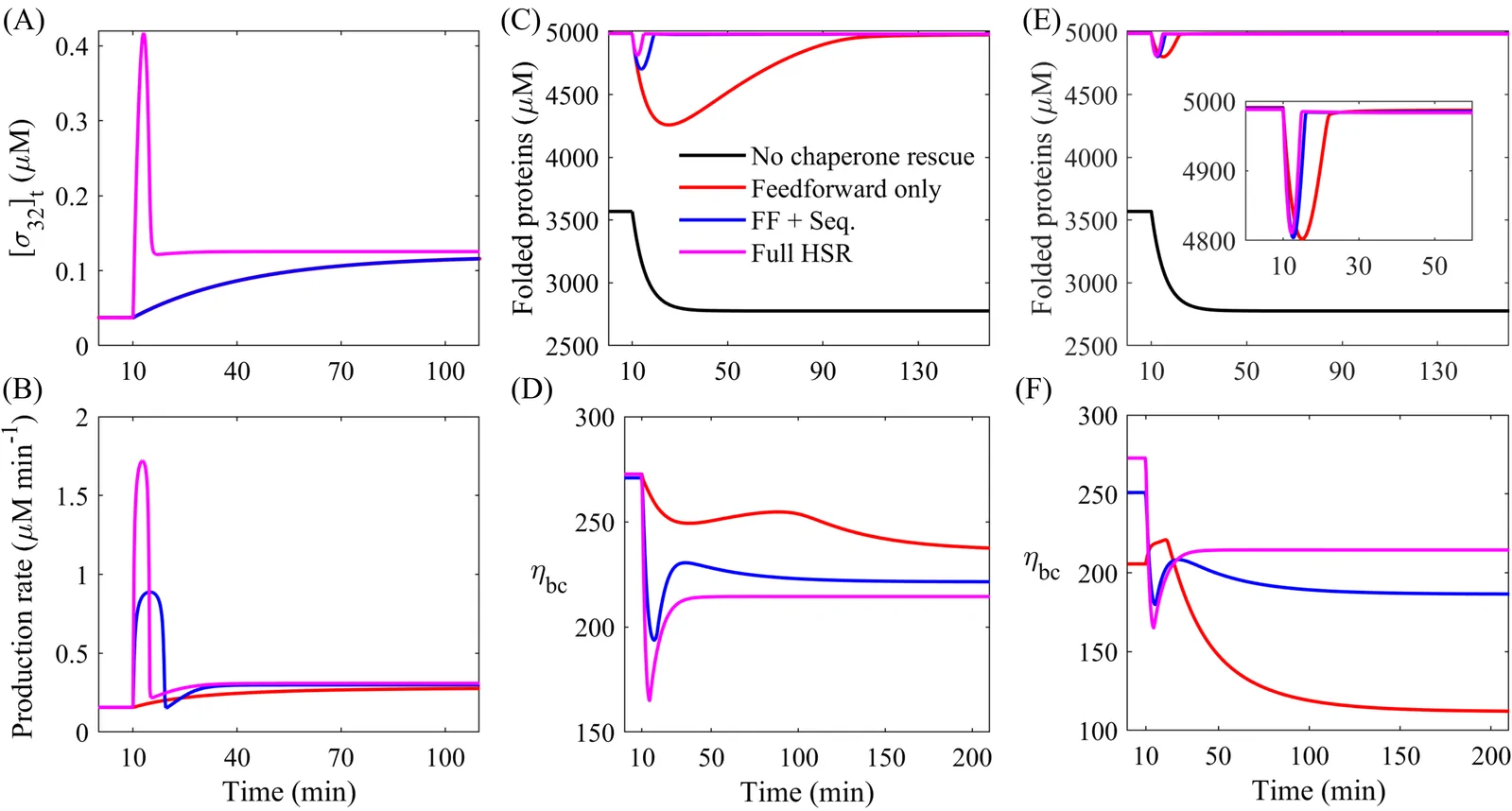
Comparison of regulatory architectures under heat shock. (A) Concentration of $[\sigma_{32}{]}_{t}$, (B) production rate of chaperones, (C,E) folded proteins, and (D,F) benefit-to-cost are plotted for feedforward (red line), feedforward with sequestration (blue line) and full HSR with feedforward, sequestration, and degradation (magenta line). Heat shock is introduced at time 10 min. The black line in (C,E) corresponds to folded proteins in the absence of chaperones. For panels (A–D), pre–heat shock steady-state levels were matched across all modules. Subsequently, the translation rates were increased in the reduced modules to match the recovery dynamics of the full HSR, and the resulting behavior is shown in panels (E,F).

The heat shock response (HSR) mechanism in *E. coli* is tightly regulated by feedback loops. To dissect their individual role and associated trade-offs in the regulation of the stress response, we studied the temporal dynamics of HSR under different regulatory configurations, *i.e.*, feedforward only, feedforward with sequestration and full HSR (feedforward, sequestration, and degradation). We calculated the time evolution of the intracellular concentration of the heat shock sigma factor, $\sigma_{32}$, as well as the rate of chaperone production, for each of the variants of the feedback module. The model was allowed to reach steady-state pre-stress conditions, after which a heat shock was applied at t = 10 min. We calibrated the reduced models to match the pre-stress steady-state levels of $\sigma_{32}$ and chaperones to those of the full HSR module (see Methods). The resulting dynamics of $\sigma_{32}$ and chaperone production rate are shown in Fig 2A and 2B, enabling direct comparison of how each feedback architecture affects both activation kinetics and resource allocation within the HSR machinery. When considering only translational control on $\sigma_{32}$ synthesis*, i.e.*, feedforward only, we observed an increase in $\sigma_{32}$ concentration and, correspondingly, in chaperone production (red line), as the heat shock enhances $\sigma_{32}$ translation. Incorporating sequestration of $\sigma_{32}$ by chaperones yielded a similar increase in $\sigma_{32}$ concentration (blue line), but with a pronounced peak in chaperone production at the onset of heat shock, followed by a drop to a steady state close to that of the feedforward module. This transient peak is a result of sequestration-based regulation. Because, under normal conditions, chaperones bind to $\sigma_{32}$, keeping its activity low. At the onset of stress, they trigger a twofold response: they unbind to assist misfolded proteins, while simultaneously releasing $\sigma_{32}$ to bind RNA polymerase and initiate heat shock gene expression. As folded proteins are recovered, chaperones rebind $\sigma_{32}$, reducing its activity and thus production. This feedback mechanism also adds an extra layer of stress sensing that is driven by the accumulation of misfolded proteins. In the full HSR module, we observe a sharp peak in $\sigma_{32}$ concentration that later drops to a steady state close to the other two feedback modules (magenta line). We also observed a sharp symmetric peak in chaperone production, closely matching experimental observations [21,28]. (Note that we validated our model with three independent experimental observations including steady state levels, transient fold changes and full time-course dynamics; see S1 Text for more details regarding model calibration and validation). This behavior is driven by the rapid translation of heat shock factors and chaperones that accelerate the protective response, along with the faster degradation of $\sigma_{32}$ that quickly reduces the expression of chaperones once homeostasis is restored. This complex regulation of $\sigma_{32}$ allows the cell to initiate a strong, timely response and faster shut-off.

Further, to study the effect of feedback modules on protein folding, we plotted the concentration of folded proteins for the three modules and compared them with the folding state in the absence of chaperones (Fig 2C). Without a rescue mechanism, the folded protein level remained very low and dropped even further during heat shock (black line). In contrast, all three modules maintained high levels of folded protein, showing an initial drop followed by recovery during heat stress. Among them, the full HSR exhibits the fastest recovery, and the drop in folded proteins is also minimal. It is followed by the feedforward with sequestration module, which is slightly slower, while the feedforward alone is the slowest and experiences the greatest drop in folded proteins at heat shock. Beyond this rapid recovery, does the full HSR offer additional advantages? To address this, we define a parameter called the *Benefit-to-Cost*, which measures chaperone efficiency by quantifying the contribution of each chaperone molecule in improving protein folding levels (see Methods for details). As shown in Fig 2D, this ratio remained nearly comparable across all three modules (at t > 100 min), despite the fact that the full HSR achieved the fastest recovery.

To examine whether the slower modules could match the rapid recovery of the full HSR, we increased the translation rates of $\sigma_{32}$ and subsequent chaperone production in the feedforward and feedforward with sequestration modules. This produced a response comparable to the full HSR, but at the cost of reduced benefit-to-cost both before and after heat shock (Fig 2E and 2F). This analysis reveals a trade-off between response time and benefit-to-cost across different modules. The trade-off shows that response time and benefit-to-cost ratio cannot be optimized simultaneously. However, the presence of all three modules enables the full HSR module to better balance rapid response with high chaperone efficiency. Together, these results are consistent with architecture-dependent differences under controlled baseline assumptions (see S3 Text for more details). This interpretation is further supported by the non-calibrated comparison presented in the S2 Text. In addition to that, while relying solely on translational control can accelerate the protective response, it may lead to inefficient utilization of chaperones, highlighting the need for an integrated regulatory framework.

### Slowing of response time at high feedforward strength

**Fig 3:**
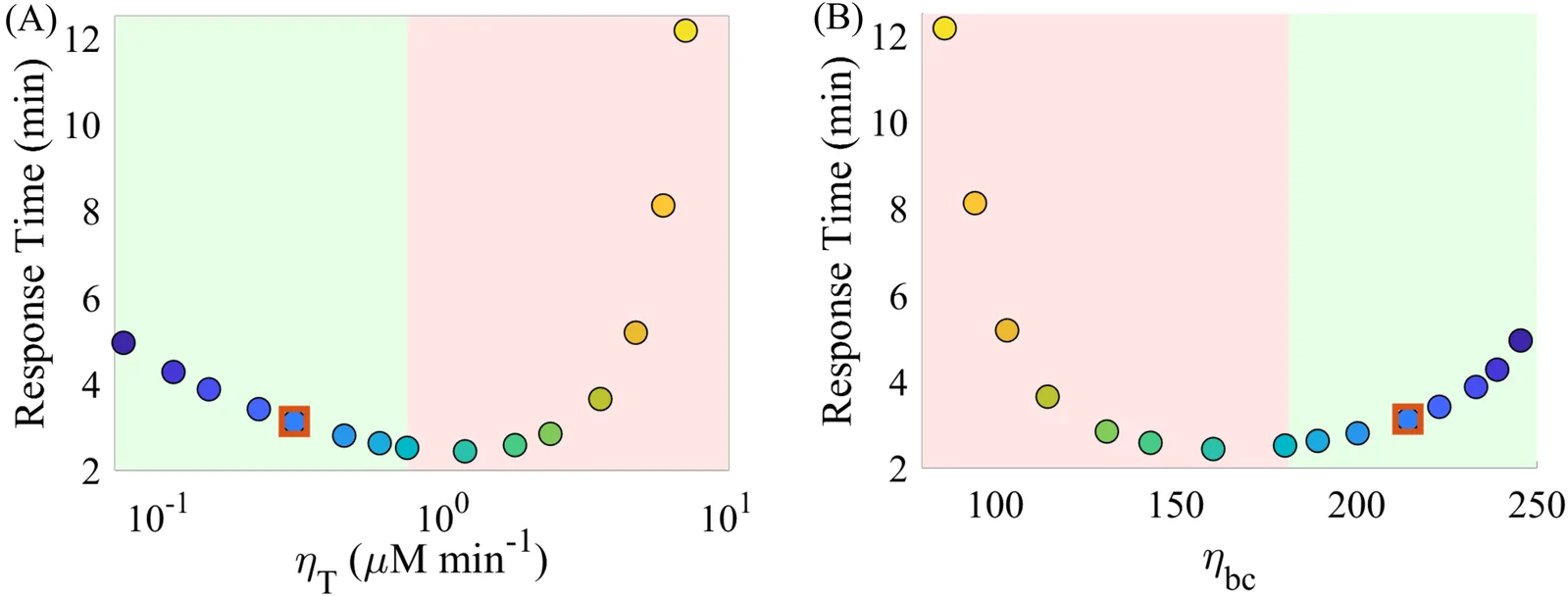
Nonmonotonic response time and emergence of synergistic and trade-off regimes with increasing feedforward strength. Response time for varying feedforward strength ($\eta_{T}$ values) is plotted against $\eta_{T}$ and benefit-to-cost ratio in (A,B), respectively. The green and red shaded areas represent trade-off and synergistic regimes, respectively. The red square represents the system at wild-type rate parameters. The gradient from blue to yellow represents increasing $\sigma_{32}$ sequestration with $\eta_{T}$,which also affects the timing of chaperone availability for misfolded proteins.

We have shown that the full HSR, integrating all three modules, achieves the fastest response while simultaneously maintaining an optimal benefit-to-cost ratio. Building on this, we next examine how the strength of each module influences the heat shock response. Specifically, we aim to determine whether increasing module strength consistently improves HSR performance or gives rise to nonlinear, nonmonotonic trends that shapes the heat shock recovery. To this end, we study the heat shock response for varying feedforward strengths, i.e., $\eta_{T}$ values. As expected, the concentration of $\sigma_{32}$, and consequently the chaperone production rate increases with the feedforward strength ($\eta_{T}$), while the benefit-to-cost ratio decreases (S1 Fig). Surprisingly, the response time exhibits a nonmonotonic trend (Fig 3A), showing a paradoxical slowing at high $\eta_{T}$, even if the chaperone concentration is high (see S2 Fig). The fastest recovery occurs with a response time of 2.4 min, compared to 3.1 min in the wild-type system (red square Fig 3A). Note that unlike the response time, the steady state folding yield remains nearly constant (S3 Fig), suggesting a timing mismatch between the availability of chaperones for misfolded proteins and their sequestration by $\sigma_{32}$. It is evident from the sharp increase in the concentration of $\sigma_{32}$ bound chaperones, while the concentration of chaperones bound to misfolded proteins always reaches a comparable peak, but more slowly at high $\eta_{T}$ (S4 Fig). This is because, at high $\eta_{T}$, the translation of $\sigma_{32}$ increases rapidly, which in turn increases the production of heat shock proteins. However, after a certain threshold, the excess $\sigma_{32}$ sequester chaperones before they could bind to misfolded proteins and assist them. In this regime, sequestration feedback dominates and restricts further (over) production of chaperones.

We also plot response time versus benefit-to-cost ($\eta_{bc}$) in Fig 3B, since both rapid recovery and high benefit-to-cost are desirable but potentially conflicting objectives. Again, we identify two operating regimes: (i) a *synergistic regime* where both metrics improve simultaneously and (ii) a *trade-off regime* where improving the benefit-to-cost ratio slows the response time. The trade‑off regime corresponds to $\sigma_{32}$ concentrations of approximately 300–700 molecules per cell (green shading), consistent with physiological levels in *E. coli* during heat shock [20,33]. Furthermore, mapping these regimes to Fig 3A reveals that the trade-off regime corresponds to lower values of $\eta_{T}$, where increasing $\eta_{T}$ accelerates recovery. In this region, the benefit-to-cost ratio is higher than in the synergistic regime, while the response time remains comparable, representing an optimal balance. The wild-type rate parameters (red square) fall within this trade-off regime, suggesting evolutionary selection for lower feedforward strength to balance rapid recovery with efficient resource use.

We also study the effect of varying sequestration (α) and degradation (β) strengths on HSR performance (see S5 and S6 Figs). Sequestration strength is varied by multiplying the forward rate constant (*k*_1_) for chaperone binding to $\sigma_{32}$ by a factor of α. Increasing sequestration strength (α) produced only a trade-off regime (S5 Fig), where both benefit-to-cost and response time increased due to tighter regulation on the availability of free $\sigma_{32}$ and consequently on chaperone production. In contrast, increasing the degradation strength (β) shows both synergistic and trade-off regimes (S6 Fig). At very low degradation strength, the response time remains fast due to high chaperone levels (low benefit-to-cost); however, at high β, both response time and benefit-to-cost ratio improve, indicating a more balanced regime. These results show that while feedforward and sequestration introduce trade-offs between speed and efficiency, the model predicts that degradation can improve both under appropriate conditions. Thus, coordinated regulation among these modules is required to achieve robust heat shock recovery.

### Sequestration feedback optimizes the adaptation time

**Fig 4:**
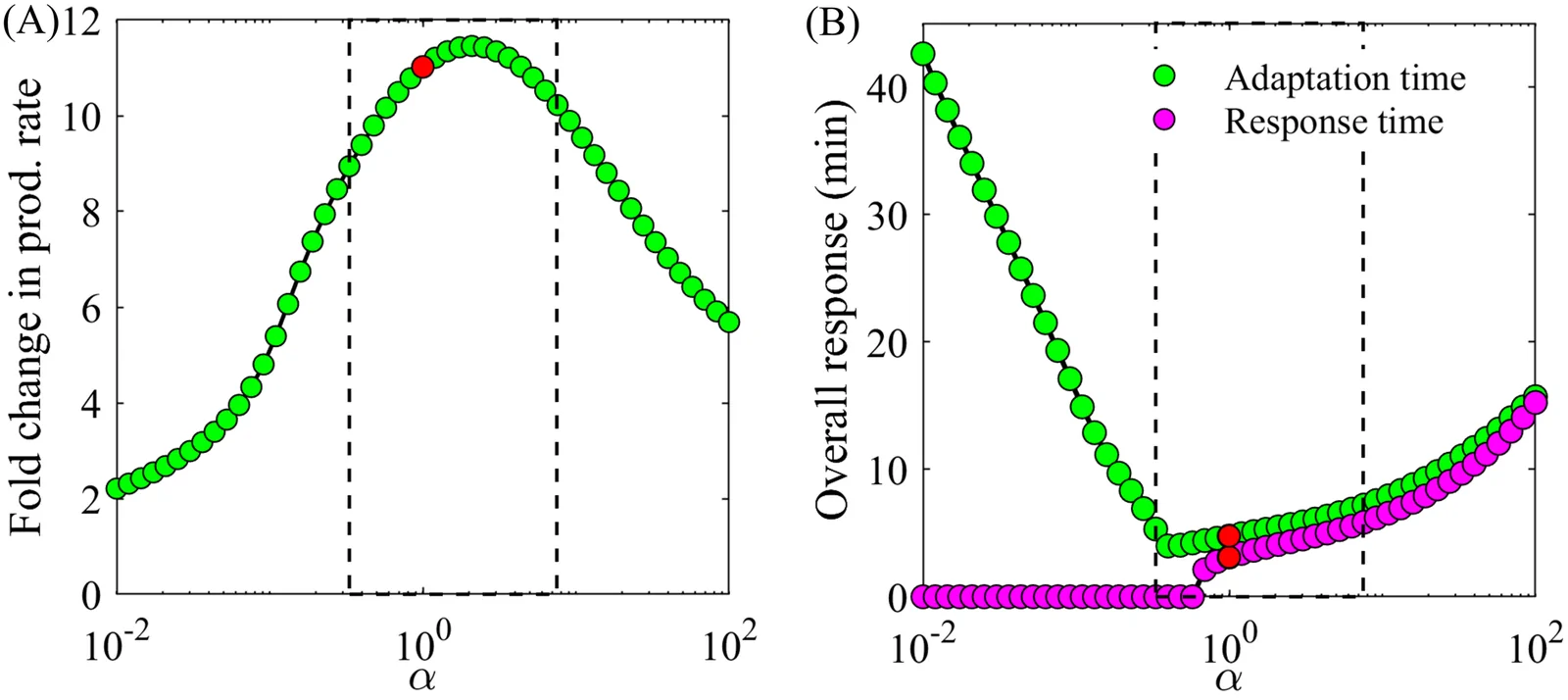
Nonmonotonic effects of sequestration strength on induction and recovery dynamics of HSR. (A) Fold change in the peak production rate relative to the pre-stress production, and (B) adaptation and response times are plotted for varying sequestration strength (α). The red dot corresponds to the wild-type parameter set. The region enclosed inside dashed lines represent optimal regime.

The sequestration module through the formation of the $\sigma_{32}\cdot C$ complex, exerts a dual influence on chaperone regulation. Under normal conditions, it suppresses chaperone production by binding and inactivating the transcription factor $\sigma_{32}$. At the same time, this interaction forms a latent reservoir of $\sigma_{32}$ that can be rapidly mobilized. During stress, chaperones dissociate from this complex to engage misfolded proteins, releasing $\sigma_{32}$ to initiate the production of more HSPs. It is therefore important to understand how this module switches between these two opposing roles: acting as a “reservoir of $\sigma_{32}$“ to amplify chaperone production and as a “degradation facilitator” to terminate the response. To explore this interplay, we first analyze how the rate of chaperone production depends on the sequestration strength α (see S7 Fig). We observe that the peak in chaperone production varies nonmonotonically with α, reaching its highest value at moderate sequestration strength (S7 Fig), while the pre-stress production rate steadily decreases. To capture these opposing effects together, we plot the fold change, defined as the ratio of the peak production rate to the pre-stress production rate, as a function of α in Fig 4A. This also shows a nonmonotonic trend, with a maximum of 10–12 fold increase at moderate sequestration strengths.

Initially with α, the fold change increases (Fig 4A). Because, sequestration builds up a large reserve pool of the $\sigma_{32}\cdot C$ complex that is released under stress. This extra supply boosts chaperone production, while the lower pre-stress production further amplifies the fold change. However, towards higher α, the fold change decreases. This is because, under excessive sequestration, $\sigma_{32}$ remains bound in complexes even during stress, which limits its access to RNA polymerase and reduces the peak production rate and fold change.

To examine this further, we define the adaptation time as the time required for the cell to initiate its stress response and then down-regulate to reach a new steady state. This metric is critical for cellular fitness: a longer adaptation time indicates that chaperone production remains elevated, keeping the cellular machinery highly engaged in the stress response and potentially delaying other essential processes. We plot adaptation time as a function of α in Fig 4B (green dots). As α increases, $\sigma_{32}$ is targeted more effectively to the degradation machinery, leading to a decrease in adaptation times. At very high α, adaptation time increases again. In this regime, excessive sequestration limits the availability of free heat shock factors and chaperones (see S8 Fig). With too few chaperones to assist misfolded proteins, the stress signal persists, keeping the HSR active (S7 Fig). Thus, adaptation time depends nonmonotonically on α, as both extended engagement of stress machinery and insufficient chaperone production can delay shutdown, depending on the regime. We also plot the response time as a function of α in Fig 4B (magenta dots). Up to a certain α, the response time remains zero, indicating that the folded proteins never drop below the set threshold (97% of the pre-heat shock values). Beyond this point, the response time increases. This transition occurs at moderate sequestration strength, close to the minimum of adaptation time.

The wild-type rate parameters (red dot in Fig 4) fall within a regime (inside the dashed region) where adaptation and response times are both low and the fold change in chaperone production is high. Deviations from this optimal regime in either direction reduce response efficiency: higher α overinhibits chaperone production, slowing the response, whereas lower α overactivates it, keeping response time low but prolonging adaptation. Importantly, while individual chaperone effectiveness (*i.e.*, benefit-to-cost) may increase with sequestration, beyond this optimal range (dashed region) it comes at the cost of prolonged production, delayed recovery, and reduced folding yield.

We also analyzed the effect of protein misfolding kinetics (γ) on the HSR dynamics (S9 Fig). To do so, we multiplied both the forward ($k_{um}$) and backward ($k_{mu}$) rates of protein misfolding, thereby increasing the kinetics without altering thermodynamic stability. Increasing γ does not change the overall qualitative behavior, but it shifts the maximum fold change, adaptation time, and the onset of response delays toward lower α (S9 Fig). At the same time, the fold change magnitude decreases and the overall response becomes slower. This occurs because, at higher misfolding kinetics, direct transitions between misfolded (M) and unfolded (U) states occur more frequently, increasing the M/U ratio. Therefore, a higher chaperone levels are needed to counteract this increased pool of misfolded proteins arising from faster misfolding kinetics. At lower α, the concentration of $\sigma_{32}$ bound in chaperone complexes and its flux to degradation are reduced, leaving more free $\sigma_{32}$ to drive chaperone production (see S7 and S8 Figs for more details). Consequently, the system shifts to lower α, which also increases the pre-stress chaperone production and thereby reduces the overall fold change. These results show that sequestration regulates chaperone production, but its strength must be optimized to balance high production with efficient shutdown. This optimal level depends on misfolding kinetics and chaperone binding affinities for misfolded proteins and $\sigma_{32}$.

### Synergies and trade-offs in the regulation of HSR

**Fig 5:**
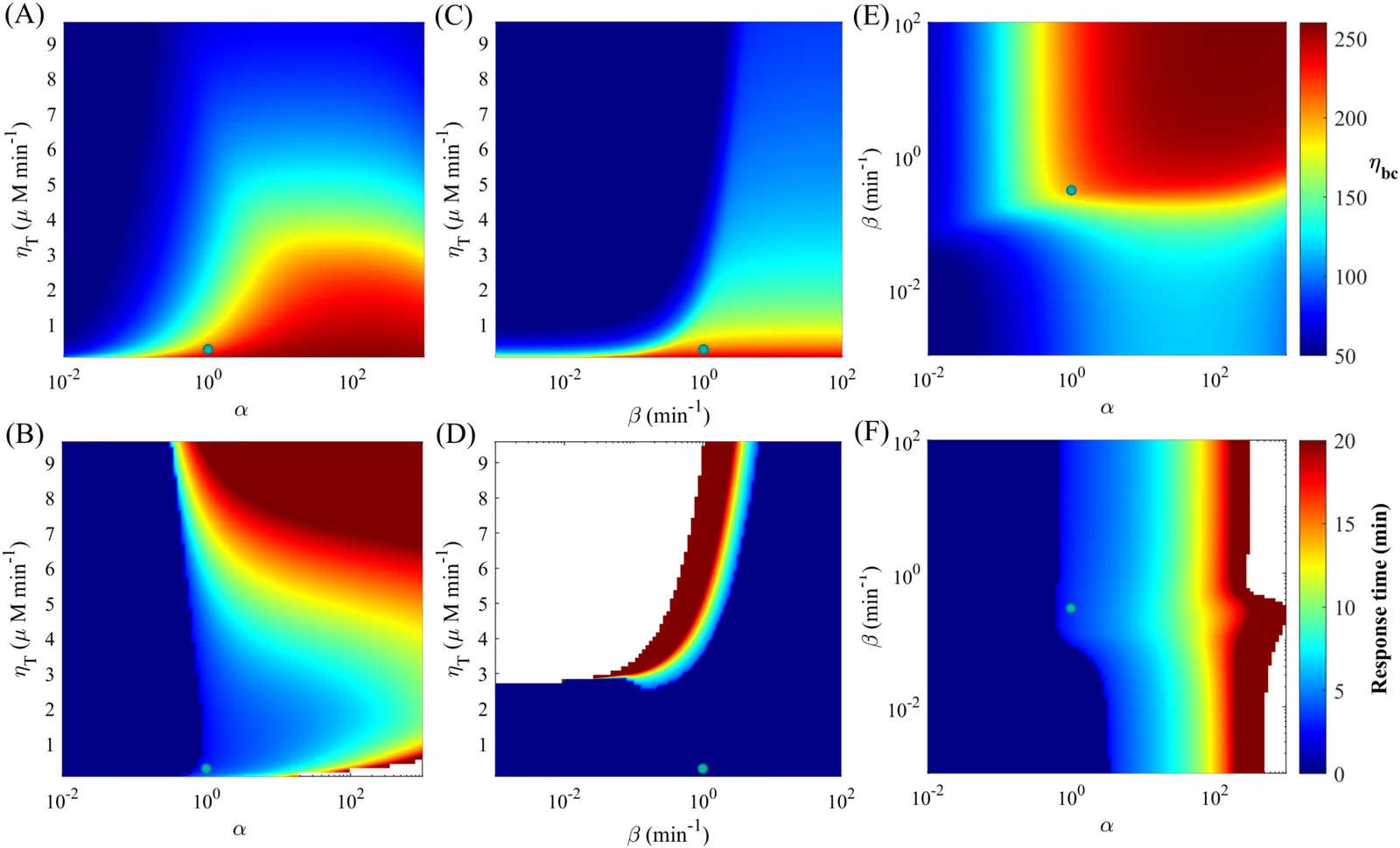
A fast and efficient response arises through cooperative and competitive interactions among feedback modules. Heat maps showing the variation in benefit-to-cost (top) and response time (bottom) across different combinations of feedback strengths: (A,B) feedforward vs. sequestration, (C,D) feedforward vs. degradation, and (E,F) degradation vs. sequestration. Each plot illustrates how pairwise tuning of feedback mechanisms influences system performance during heat shock response. The green dot represents the system at wild-type rate parameters. The white regions show parameter combinations where recovery of folded proteins is below 97%.

Based on the analysis of individual contributions of feedforward, sequestration, and degradation, we further examine how these feedback modules interact and influence one another. Our goal is to identify the synergies and trade-offs that emerge from their combined effects. To understand this, we first varied the strengths of feedforward ($\eta_{T}$) and sequestration (α) while keeping the degradation (β) fixed (Fig 5A and 5B). As expected, stronger sequestration improves the benefit-to-cost ratio but slows down the response time (right side of Fig 5A and 5B) and reduces the folding yield (S10 Fig). At low sequestration, the response remains fast, but with a low benefit-to-cost ratio (left side of Fig 5A and 5B). This suggests that both extremes of sequestration lead to suboptimal regulation via different mechanisms. In contrast, stronger feedforward feedback increases chaperone production, enabling faster recovery and higher folding yield, albeit at the expense of a reduced benefit-to-cost ratio (Figs 5A and 5B and S10). These results indicate that independently strengthening feedforward ($\eta_{T}$) or sequestration (α) leads to suboptimal performance, as the benefit of one is offset by its inherent cost. However, analysis of their combined parameter space shows that co-tuning these feedback modules is essential to achieve an optimal balance between response speed and chaperone efficiency. Furthermore, the response time shows non-monotonic behavior at moderate η and α values (as in Fig 3). This is where the binding affinity between heat shock factor and misfolded proteins is comparable, leading to rapid release and re-sequestration of $\sigma_{32}$. Therefore, the response remains rapid in this parameter space.

Next, we varied the feedforward and degradation feedback strengths while keeping sequestration fixed at a moderate level (Fig 5C and 5D). Increasing degradation consistently improves both the benefit-to-cost ratio and response time. Stronger degradation also mitigates the slowdown caused by increasing $\eta_{T}$ (red region decreases in Fig 5D) and the white space corresponding to less than 97% recovery disappears with increasing β. This occurs because excess $\sigma_{32}$ that would otherwise be sequestered is now actively degraded, freeing chaperones to assist misfolded proteins and improve folding yield (S11 Fig). Thus, the model predicts that degradation acts synergistically with feedforward, accelerating recovery by increasing the pool of active chaperones and limiting excess $\sigma_{32}$. However, its beneficial effect saturates at higher feedforward strengths, beyond which performance gains are minimal (right side of Fig 5C). This is because, at high $\eta_{T}$, excessive translation of $\sigma_{32}$ saturates the degradation pathway. Now, the accumulated $\sigma_{32}$ continues to drive chaperone production, increasing the cost. As a result, the benefit-to-cost ratio plateaus and cannot be improved further. In this case, the optimal regime emerges with a relatively low feedforward strength paired with high degradation, balancing a rapid response with efficient resource use.

Finally, we examine the combined effects of sequestration and degradation while keeping the feedforward fixed at a low level (Fig 5E and 5F). Interestingly, at low degradation strength, the benefit-to-cost ratio varies nonmonotonically with α (Fig 5E). This is because sequestration reduces free $\sigma_{32}$, lowering excess chaperone production and thereby improving the ratio. However, beyond a threshold, stronger suppression of $\sigma_{32}$ activity limits chaperone production and availability. As a result, folding yield (*i.e.*, the numerator of $\eta_{bc}$) decreases (S12 Fig), which lowers the benefit-to-cost ratio and creates a peak at moderate sequestration strength. In contrast, at high β, benefit-to-cost ratio improves and eventually saturates with increasing α. This is because high degradation removes excess $\sigma_{32}$, limiting chaperone overproduction and increasing the availability of free chaperones. Consequently, although folded protein levels decrease (S12 Fig), the benefit-to-cost ratio improves as fewer chaperones are required per folded protein. Importantly, high sequestration alone cannot achieve a high benefit-to-cost ratio without rapid degradation, and degradation alone is ineffective without sequestration, which promotes the binding and removal of $\sigma_{32}$-chaperone complex. In addition, a higher sequestration slows the response due to a stronger suppression of $\sigma_{32}$ activity. At high sequestration strength, degradation modulates the allocation of chaperones between $\sigma_{32}$ and misfolded proteins, which compete for a shared chaperone pool. When degradation is low, chaperones are primarily bound to $\sigma_{32}$, limiting their availability for folding and resulting in a slower response. As degradation increases, a fraction of chaperones is freed from $\sigma_{32}$ and can bind more readily to misfolded proteins, thereby improving the response time. However, at sufficiently high degradation, $\sigma_{32}$ levels are strongly suppressed, reducing chaperone production and total chaperone availability, which again slows the response. Therefore, the model predicts that the best operating regime combines moderate sequestration with high degradation. Interestingly, the wild-type parameter set (green dot) always lies within these optimal regimes of the explored parameter space, consistent with the regulatory strategy revealed by our analysis.

### Optimal regime of operation

Competition between feedback modules shapes the trade-off between response time and benefit-to-cost (Fig 5), suggesting the existence of an optimal operating regime. To capture this, we define a composite metric, performance index ($\eta_{P}$), that integrates rapid recovery and efficient chaperone utilization into a single measure (see Methods for details).

**Fig 6:**
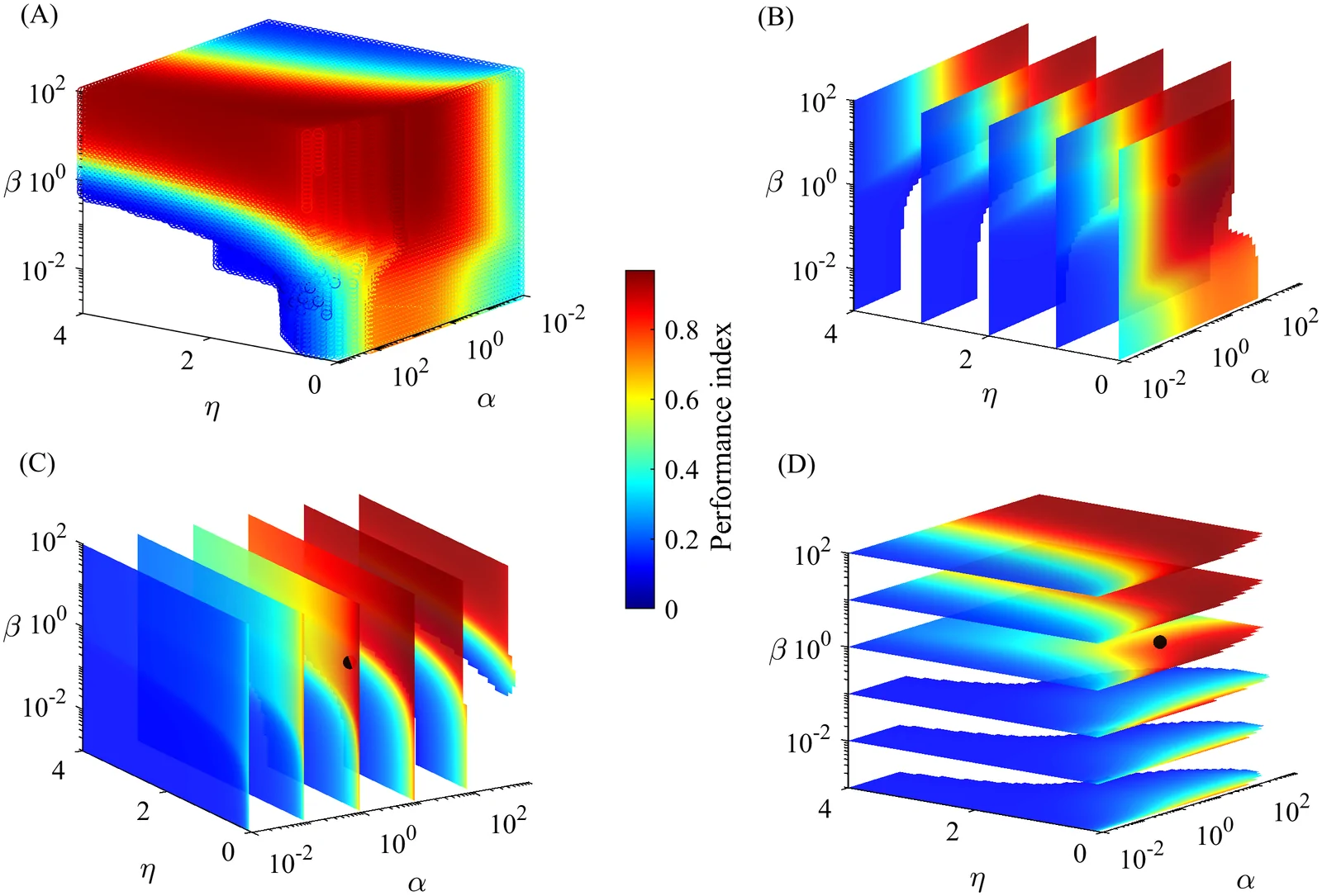
Balanced optimization of heat shock performance across regulatory modules. (A) Performance index plotted as a function of feedforward, sequestration, and degradation feedback strengths. Slice plots showing side and top views of the performance index are shown in (B), (C), and (D), respectively. The black dot in (C) and (D) corresponds to the wild-type parameter set.

We plotted the performance index as a function of feedforward, sequestration, and degradation feedback strengths in Fig 6. The red region marks the optimal regime, characterized by fast recovery and high chaperone efficiency, whereas the white region corresponds to parameter regimes where the HSR fails to achieve a 97% folding yield. With increasing $\sigma_{32}$ translation and sequestration, the HSR struggles to achieve the desired response (97% recovery of folded proteins) regardless of the benefit-to-cost ratio, leading to an expansion of the white region (Fig 6B and 6C). This is consistent with the findings in Figs 3-5, showing that excessive feedforward or sequestration impairs recovery by triggering overproduction or limiting chaperone availability. Furthermore, the region with a high performance index shrinks rapidly when $\sigma_{32}$ production is high but sequestration and degradation remain weak (red region shrinks in Fig 6B and 6C). In contrast, increasing degradation rate of $\sigma_{32}$ not only improves overall performance (red region expands in Fig 6D), but also expands the region of desired response, where recovery is 97% (white spaces are filled). This highlights that timely removal of excess $\sigma_{32}$ works in concert with other feedbacks to ensure robust and efficient regulation.

To ensure that these observations are not sensitive to the specific choice of performance metrics, we repeated the analysis using alternative recovery thresholds (85% and 90%; S13 Fig). While lowering the threshold increases the fraction of parameter space capable of recovery, the qualitative structure of the performance landscape, including the location of the optimal regime, remains unchanged. In particular, parameter combinations that fail to reach 97% recovery can still achieve lower thresholds, leading to a modest reduction in the white regions. However, the emergence of synergy and trade-off regimes, as well as the identification of a high-performance operating region, are robust to these variations.

These results reveal an optimal operational regime at low feedforward and low-to-moderate sequestration strengths (red region). Incorporating strong degradation feedback shifts this regime to a higher feedforward and sequestration range. In the optimal regime, the HSR system achieves better balance between rapid response and high benefit-to-cost ratio. In contrast, regions with lower performance index reflect biased optimization, either fast but inefficient responses or efficient yet slow recovery (see S14 and S15 Figs). Finally, our model predicts that the wild-type parameter set (black dot in Fig 6) lies within the high-performance region of the explored parameter space. Moreover, the HSR is also tuned to minimize adaptation time (S16 Fig), prioritizing rapid recovery while limiting the energetic cost of prolonged chaperone production. Note that $\eta_{P}$ depends on the chosen parameter bounds and normalization procedure. It should be interpreted as a comparative metric within the sampled regime rather than as an absolute biological objective function. Nevertheless, $\eta_{P}$ serves as a composite metric for visualizing regions where rapid recovery and efficient chaperone utilization are jointly favored. However, a comprehensive assessment of HSR performance also requires examining response time and $\eta_{bc}$ separately (S14 and S15 Figs). While these two objectives cannot be optimized simultaneously, regions with high $\eta_{P}$ identify parameter combinations that achieve a favorable compromise between rapid recovery and efficient chaperone utilization within the explored parameter space. These observations suggest that the HSR is not optimized for a single metric but represents a balanced compromise among response speed, adaptation time, and chaperone efficiency, a likely consequence of evolutionary pressures.

Additionally, we performed both one-at-a-time sensitivity and correlated multi-parameter perturbation analysis to identify the influence of individual parameters on model behavior and to evaluate the robustness of our conclusions to simultaneous uncertainty in multiple interacting parameters (see S4 Text for details). Together, these analyses demonstrate that the principal conclusions of this study arise from the underlying HSR regulatory architecture rather than from a particular choice of parameter values.

### Effect of ATP availability on HSR dynamics

**Fig 7:**
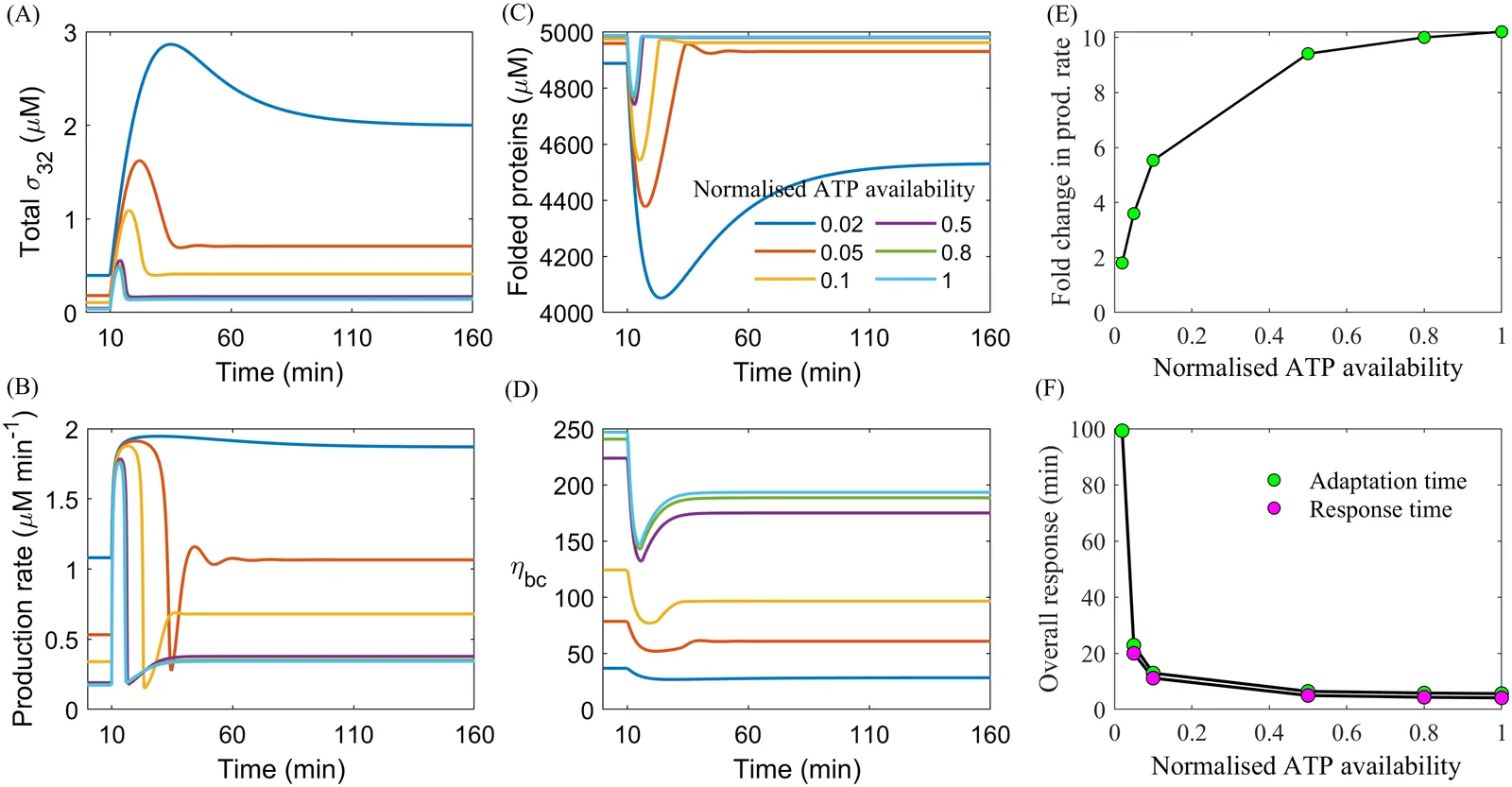
Heat shock response dynamics under varying normalized ATP availability for *K*_A_ = 0.3. (A) Total $\sigma_{32}$, (B) chaperone production rate, (C) folded proteins, (D) benefit-to-cost ratio, (E) fold change in production rate, and (F) response and adaptation times. ATP availability is normalized between limiting (0) and saturating (1) conditions.

ATP plays a central role in both chaperone-mediated protein folding [40,41,53] and FtsH-dependent degradation of $\sigma_{32}$ [30,31]. To incorporate this, we model ATP dependence using a Michaelis-Menten type formulation. Here, ATP concentration and the corresponding Michaelis constant are defined as normalized dimensionless variables, *A* and $K_{A}$, respectively (see Methods for details). We then analyze how ATP availability influences HSR dynamics by examining total $\sigma_{32}$ levels, chaperone production rate, folded protein levels, benefit-to-cost ratio, fold change in production rate, response time and adaptation time across normalized ATP availability for a range of *K*_A_ (Fig 7).

As ATP levels decline, steady-state $\sigma_{32}$ increases due to reduced protease-mediated degradation (Fig 7A). At the same time, folded protein levels decrease (Fig 7C), and both response and adaptation times become longer (Fig 7F). Although ATP limitation leads to accumulation of $\sigma_{32}$ and increased chaperone production (Fig 7B), this does not translate into improved folding yield (Fig 7C). This reflects the ATP dependence of chaperone-mediated refolding, where insufficient ATP limits substrate turnover. Consequently, even when more chaperones are present, the overall folding process becomes slower. In addition, elevated $\sigma_{32}$ also competes with misfolded proteins for the available chaperone pool, further limiting chaperone-assisted protein folding.

ATP limitation also impacts HSR performance metrics. Both the benefit-to-cost ratio and the fold change in production rate decrease with decreasing ATP (Fig 7D and 7E). Under strongly ATP-limited conditions, chaperone production remains elevated both before and after heat shock, rather than exhibiting the characteristic transient increase followed by a decline observed at higher ATP levels. This sustained chaperone production (thus, high chaperone concentration), together with reduced folding yield, greatly lowers the overall benefit-to-cost ratio. Consequently, the relative fold change in production rate is also reduced.

The extent of these effect depends on the ATP sensitivity parameter *K*_A_. As $K_{A}$ increases, the impact of ATP limitation becomes more pronounced. Because larger $K_{A}$ values reduce the effective sensitivity of reactions to ATP, leading to greater accumulation of $\sigma_{32}$ (S18A Fig), sustained chaperone production (S18B Fig), impaired recovery of folded proteins (S18C Fig), and slower response dynamics (S18F Fig). In contrast, for smaller *K*_A_ values, reactions approach saturation even at moderate ATP levels, making HSR less sensitive to ATP depletion (S17 Fig).

Overall, higher ATP availability improves response time and folding efficiency, enabling faster adaptation to heat shock. At the same time, the qualitative behavior and core conclusions remain robust to variations in cellular energy levels.

### Effect of irreversible aggregation on HSR dynamics

Irreversible protein aggregation can influence protein folding kinetics during heat shock and may alter recovery dynamics. Thus, to examine its impact on synergies and trade-offs between regulatory modules, we extended the model to include an irreversible aggregation pathway for misfolded proteins.

In the extended model, misfolded proteins form irreversible aggregates with rate $k_{agg}$. We considered aggregation rates of $k_{agg}=0.01{min}^{-1}$ under basal conditions and $k_{agg}=0.05{min}^{-1}$ during heat shock, consistent with experimentally reported values for protein aggregation kinetics [63,64]. Unfolded proteins are produced at a rate of $9\mu {Mmin}^{-1}$, representing effective proteome-level fluxes [54,55]. To make the protein more aggregation-prone, we reduced the refolding rate $k_{uf}$ by 5 times, thereby increasing the persistence of misfolded proteins. Other parameters were maintained as in the main model (Fig 1).

As in Fig 2, we plot the total $\sigma_{32}$, folded proteins, chaperone production rate, and benefit-to-cost ratio for the extended model (S19 Fig). Including irreversible aggregation does not change the qualitative behavior of the system. Among the three regulatory modules, the full HSR module still shows the fastest recovery and the smallest transient drop in folded proteins (S19B Fig). It also shows a strong induction of chaperone production followed by rapid shutdown (S19D Fig). However, the overall benefit-to-cost ratio is reduced in all modules (S19E Fig) compared to the main model (Fig 1) as the continuous influx of unfolded proteins and increased misfolding propensity reduce the number of proteins that can be successfully rescued by chaperones.

We increased the translation rates of $\sigma_{32}$ and chaperones in the feedforward-only and feedforward with sequestration modules to match the recovery dynamics of the full HSR as done in Fig 2. Consistent with our earlier results, this accelerates recovery in both modules (S19C Fig) at the cost of reduced benefit-to-cost ratio (S19F Fig). However, the feedforward-only module displays only a modest improvement, experiencing the largest transient loss of folded proteins and the slowest recovery. This occurs because irreversible aggregation further amplifies the consequences of delayed chaperone assistance in the feedforward-only module, which lacks a transient peak in chaperone production during stress. Consequently, even after increasing translation rates, it shows limited improvement and exhibits a lower benefit-to-cost ratio compared to the other modules. Further increases in translation would accelerate recovery only at the expense of additional reductions in the benefit-to-cost ratio. In contrast, the full HSR module consistently achieves both faster recovery and a higher benefit-to-cost ratio. Thus, even in the presence of irreversible aggregation, the inclusion of all three regulatory modules allows the full HSR module to balance rapid response with chaperone efficiency, once again highlighting the trade-off between response time and benefit-to-cost across different regulatory architectures.

Furthermore, to examine how irreversible aggregation influences synergy and trade-offs between regulatory modules, we repeated the analyses using the extended model. We find that the qualitative behavior of the HSR remains consistent with the main model (Fig 1). In particular, the response time continues to show a nonmonotonic dependence on feedforward strength $\eta_{T}$ (S20A Fig). As in Fig 3, two regimes are observed: a synergistic regime in which response time and benefit-to-cost improve together, and a trade-off regime in which further increases in benefit-to-cost are accompanied slower recovery (S20B Fig). However, in contrast, at high $\eta_{T}$ the response time shows longer delays. This arises because irreversible aggregation and reduced refolding slow the clearance of misfolded proteins in the presence of a continuous influx of unfolded species during stress. Consequently, a temporary reduction in chaperone assistance (see Fig 3 for explanation) amplifies recovery delays, producing the observed asymmetry at high $\eta_{T}$.

Similarly, varying sequestration strength α produces trends qualitatively comparable to those observed in Fig 4. Both the fold change in production rate and the response dynamics show a nonmonotonic dependence on α (S21 Fig). However, in the extended model, folded protein levels fail to reach the 97% recovery threshold at high sequestration strengths (magenta dots in S21B Fig). This occurs because strong sequestration suppresses $\sigma_{32}$ activity, limiting chaperone production; in the presence of increased misfolding and irreversible aggregation, this prevents the complete recovery of folded proteins. The wild-type rate parameter set (red dot in S21 Fig) lie within a regime where adaptation and response times remain low and fold change in production rate is high.

Next, we examine how these feedback modules interact. As in Fig 5, we first varied feedforward strength ($\eta_{T}$) and sequestration strength (α) while keeping the degradation (β) fixed (S22A and S22B Fig). We again observe that independently strengthening feedforward ($\eta_{T}$) or sequestration (α) leads to suboptimal performance, indicating that co-tuning of these feedback modules is essential to balance response speed and chaperone efficiency. We then varied feedforward ($\eta_{T}$) and degradation (β) while keeping sequestration (α) fixed (S22C and S22D Fig). Consistent with our previous results, degradation acts synergistically with feedforward, simultaneously improving benefit-to-cost (S22C Fig) and accelerating recovery (S22D Fig) except at very low $\eta_{T}$. Finally, varying sequestration (α) and degradation (β) at fixed feedforward ($\eta_{T}$) (S22E and S22F Fig) reveals that optimal performance occurs with moderate sequestration and high degradation. The wild-type parameter set (green dot) consistently lies in the optimal regime within the explored parameter space, reinforcing the regulatory strategy identified in our main analysis. Moreover, as also observed in the preceding analyses, high $\eta_{T}$ and high α lead to a more pronounced decline in performance in the extended model, as excessive feedforward or sequestration cannot be effectively counterbalanced under sustained misfolding flux and irreversible aggregation.

Finally, we examine the performance index as a function of feedforward, sequestration, and degradation feedback strengths (S23 Fig). Consistent with Fig 6, the qualitative structure of the performance landscape remains unchanged. However, in the extended model the high-performance region (red) is reduced, as achieving near-complete (97%) recovery of folded proteins becomes more difficult under sustained unfolded protein flux and irreversible aggregation. Consequently, the low-performance region (white) expands. In particular, very high feedforward and both extremes of sequestration strength lead to larger deviations from optimal performance in the extended model. Despite this quantitative reshaping of the landscape, the location of the optimal operating regime is preserved. The wild-type parameter set (black dot in S23 Fig) continues to lie in this optimal regime within the explored parameter space.

Overall, including irreversible aggregation quantitatively changes the efficiency of the HSR but does not alter the main conclusions of the study. The cooperative and competing relationships between feedforward, sequestration, and degradation modules remain intact, and the optimal operating regime of the heat shock response is preserved. Furthermore, to examine whether the conclusions of the extended model depend on the specific aggregation scenario considered, we systematically varied the aggregation rate, unfolded-protein influx, and protein folding rate by an order of magnitude above and below their reference values (S5 Text). The observed behavior closely replicated the qualitative conclusions of this study and these perturbations primarily altered the severity of proteotoxic stress by changing the balance between protein damage and repair. A qualitatively different picture emerged only under two conditions: when the protein folding rate was increased and when the unfolded-protein influx was reduced. In both cases, the characteristic nonmonotonic dependence of response time on feedforward strength and fold change on sequestration strength was not observed within the explored parameter range. These two cases share a common feature: proteotoxic stress is substantially milder, with reduced misfolding and aggregation tendency, where the chaperone burden is low. Despite this, the trade-off between response speed and chaperone efficiency, the paradoxical slowing of recovery at high feedforward activation, the dual regulatory role of sequestration in simultaneously enhancing chaperone production and enabling timely shutdown, the synergistic contribution of degradation, and the emergence of a high-performance operating region, remain robust across the explored parameter ranges (see S5 Text for more details). These findings demonstrate that the main conclusions of this study arise from the interplay among the three feedback modules of the HSR rather than from any particular choice of aggregation, folding, or unfolding parameters.

## Discussion

The bacterial heat shock response must coordinate protein folding with the activity of its master regulator, $\sigma_{32}$. To understand this balance between recovery and resource use, our mechanistic model explicitly couples the folding cycle with $\sigma_{32}$ regulation. We evaluate metrics such as folding yield, benefit-to-cost ratio, response time, and performance index, allowing a direct comparison of alternative regulatory architectures under stress conditions. To compare regulatory strategies, we examine: (1) feedforward translation control alone, (2) feedforward combined with sequestration, and (3) the complete HSR with all three modules. Each showed distinct performance characteristics. Feedforward alone provided steady chaperone production, but lacked the transient sharp activation necessary for rapid recovery. Adding sequestration improved the sharpness of the response by allowing a sudden release of active $\sigma_{32}$. The complete HSR reproduced an even sharper peak in $\sigma_{32}$ and chaperone production levels as observed experimentally [21,28], providing the fastest recovery overall. Our analysis also shows that simply increasing translation rates accelerates recovery, but at the expense of chaperone efficiency (Fig 2). This underscores the importance of the layered feedback architecture in the full HSR model, which enables rapid response and reduced misfolding while preserving efficiency compared to simpler regulatory designs.

The heat shock response in *E. coli* has been extensively studied both experimentally and theoretically [28,34,38,65,66]. These studies highlighted that the integration of feedforward activation with sequestration and degradation feedback not only initiates a rapid response, but also expands the kinetic range of efficient response compared to simpler regulatory designs [24,36]. Our analysis extends this perspective by showing cooperative and competitive interactions among the three feedback modules. These interactions give rise to nonmonotonic dynamics, where optimal performance arises only under specific regimes of operation. For example, although the feedforward module accelerates the response, excessive activation can saturate the degradation pathway, ultimately limiting its own benefit and slowing recovery (Figs 3 and 5). Similarly, sequestration can enhance chaperone production and accelerate adaptation within an optimal range, but beyond this regime, it suppresses production, prolongs stress response, and delays clearance (Fig 4).

Beneficial interactions between regulatory modules can become inhibitory depending on the relative strengths of these feedback modules. This behavior arises from their coupling through shared molecular components, particularly $\sigma_{32}$ and chaperones. Although this coupling enables coordinated regulation, it can also introduce competition for shared components. For example, sequestration feedback initially complements feedforward activation by regulating the availability of free $\sigma_{32}$ during stress, increasing the fold change in the heat shock protein production rate and improving response dynamics (Fig 4 of the main text). However, when sequestration becomes too strong, it limits the pool of free $\sigma_{32}$ available for transcriptional activation. This delays the response and reduces the fold change in the rate of production of heat shock proteins, thereby weakening the contribution of the feedforward module and ultimately slowing recovery. These inter-module interactions translate directly into biologically relevant quantities. As a result, antagonistic interactions at the molecular level manifest as trade-offs between recovery speed, efficient utilization of chaperones, and timely shutdown of the stress response (Figs 3-6). From a biological perspective, these trade-offs are closely related to cellular fitness, as successful adaptation requires not only surviving acute stress but also efficiently restoring normal cellular activity once stress conditions subside. Because it is not possible to simultaneously maximize multiple functional objectives, the cell must find a balance between these competing demands. The optimal regimes identified in our analysis are consistent with this, indicating parameter regions where competing functional demands are balanced rather than any single performance objective being maximized (Fig 6). While quantities such as response time and the benefit-to-cost ratio provide measures of system performance, the trade-off between them arises from the coupling between $\sigma_{32}$ regulation, chaperone availability, and degradation dynamics, which prevents these objectives from being optimized simultaneously.

At the most fundamental level, the trade-offs identified in this study arise from a single underlying constraint: the three regulatory modules, feedforward, sequestration and degradation, compete for a finite pool of $\sigma_{32}$ and chaperones. Since these molecules cannot simultaneously serve all competing demands (driving transcription, sequestering $\sigma_{32}$, refolding misfolded proteins, and targeting $\sigma_{32}$ for degradation), any regulatory action that preferentially directs them toward one functional objective may reduce their availability for another. This molecular competition manifests at three complementary levels. At the state-space level, it produces loop-like trajectories in the three-dimensional state space ($\sigma_{32}$, chaperones, protease), where the shared chaperone pool is recruited toward misfolded protein refolding during activation and toward $\sigma_{32}$ sequestration during recovery, preventing the trajectory to follow a simple monotonic rise towards steady state as seen in feedforward-only module (S3 Text). At the performance level, it shapes the relationship between response time and benefit-to-cost ratio. Because both quantities depend on the shared pool of $\sigma_{32}$ and chaperones, strategies that accelerate recovery reduce the benefit-to-cost ratio (Fig 3), while strategies that improve efficiency slow recovery (Fig 4), so the two objectives cannot be simultaneously maximized. At the model level, the nonmonotonic dependence of HSR performance on individual feedback strengths (Figs 3-5) reflects how the balance of this competition shifts with relative module strengths, giving rise to distinct synergistic and trade-off regimes. Taken together, these are not independent observations but three expressions of the same underlying constraint, and the optimal operating regime (Fig 6) represents the parameter region where this competition is most evenly balanced across all functional demands.

The trade-off between response time and benefit-to-cost ratio reflects a general feature of multi-objective optimization in biology, where fitness integrates demands belonging to fundamentally different categories [67,68]. The two objectives capture complementary dimensions of cellular fitness, the speed of protection from proteotoxic damage, and the metabolic efficiency of the protective response, both of which are relevant for survival and growth in fluctuating environments. Faster recovery improves acute stress survival, while efficient chaperone usage preserves cellular resources for post-stress adaptation and growth. Such cross-dimensional couplings are recognized within the chaperone and HSR literature: In one of our previous studies [52], we have shown a speed-energy-efficiency trade-off in the Hsp70 system where we cannot simultaneously optimize folding efficiency and energy efficiency of the process. More importantly, Kurata et al. [24] demonstrated a trade-off between folding yield and the dynamic sensitivity of DnaK concentration to parameter perturbations. In both cases, as in this study, the traded quantities are dimensionally distinct because the organism must simultaneously satisfy constraints that operate on different timescales and resources.

The HSR exhibits a strong, nonlinear dependence on feedback strengths, giving rise to distinct operating regimes. Each regime is characterized by specific synergies and trade-offs among the feedback modules. For instance, moderate increases in feedforward strength ($\eta_{T}$) speed up activation and recovery; but beyond a certain $\eta_{T}$, excess $\sigma_{32}$ triggers autoinhibition, slowing response time. This produces two regimes: a synergistic regime in which both chaperone efficiency and response speed improve together and a trade-off regime in which improvement of one comes at the expense of the other. Interestingly, in the synergistic regime, chaperone efficiency remains low even at response speeds comparable to those in the trade-off regime. The wild-type system operates within this trade-off regime, exemplifying a common biochemical strategy to avoid overactivation through built-in attenuation or negative feedback, thereby balancing responsiveness with stability [69–72]. Sequestration strength (α) creates even richer dynamics. When sequestration is weak, the HSR is dominated by chaperone production, which gives fast activation but slow shutdown. When too strong, chaperones are overcommitted to $\sigma_{32}$, leaving too few to resolve misfolded proteins. The HSR achieves the best balance between activation speed and efficient shutdown only at intermediate levels. This nonmonotonic pattern arises because $\sigma_{32}$ and misfolded proteins compete for a shared pool of chaperones, creating trade-offs between activation and adaptation.

Cells maintain protein homeostasis with the help of heat shock proteins (HSPs), but their production and maintenance is metabolically costly. The HSR therefore balances the protective benefits against cost through a layered regulatory architecture (Figs 2-4). Feedforward and sequestration often act in opposition, yet together they can offset each other’s drawbacks: sequestration reduces the metabolic cost of feedforward-driven responses, while feedforward accelerates the response otherwise delayed by sequestration (Fig 5). Sequestration couples $\sigma_{32}$ activity with the folding state of the protein, lowering baseline production via $\sigma_{32}\cdot C$ complex formation and releasing $\sigma_{32}$ under stress to boost production. Its optimal strength depends on the protein misfolding kinetics and chaperone binding affinities: faster misfolding favors weaker sequestration to drive higher chaperone production. Thus, sequestration functions as a regulatory module that coordinates activation with timely shutdown. This delicate balance between activation and deactivation is a core principle of heat shock systems across species [15,70,73], as exemplified by the regulation of the master transcription factor in eukaryotes [74,75]. Degradation, in contrast, synergizes with both modules by clearing excess $\sigma_{32}$, releasing chaperones for folding, and preventing wasteful overproduction. Consistent with previous work [23], this highlights the dual role of HSR in optimizing immediate performance while safeguarding long-term stability. Note that protease synthesis is scaled relative to chaperone synthesis using ribosome profiling data, protease regulation is represented in a simplified manner in our model. Consequently, conclusions regarding the degradation strength (β) should be interpreted as qualitative insights made by the model into the role of proteolysis in shaping HSR dynamics.

To explore the optimal operating regime of the HSR, we define a performance index that combines the response speed and benefit-to-cost ratio. Higher values indicate more efficient performance, and analyzing the metrics together or individually provides additional insight into system behavior. The wild-type parameters (black dot in Fig 6) lie within the region of optimal performance index and rapid adaptation time (S16 Fig), achieving fast recovery while limiting the energetic cost of prolonged chaperone production. This placement highlights evolutionary compromise: speed of recovery is prioritized over absolute efficiency (Fig 5), since delayed clearance of misfolded proteins poses a greater risk to survival than modest metabolic costs. Moreover, while the efficiency of individual chaperone (benefit-to-cost ratio) increases with stronger sequestration (S5 Fig), this does not ensure overall efficiency. Beyond the optimal regime (dashed region in Fig 4), improved individual effectiveness comes at the cost of prolonged production, delayed recovery (Fig 4D), and reduced folding yield (S9 Fig).

Beyond the mechanistic insights provided by our model, the identification of synergistic and trade-off regimes suggests broader evolutionary and biomedical implications. The positioning of wild-type parameters within a trade-off regime indicates that the heat shock response is not optimized for maximal activation or efficiency alone (Fig 3). Instead, it balances rapid recovery against the metabolic cost of sustained chaperone production, consistent with the general principles of resource allocation and fitness optimization [67,68]. Such regulation likely improves survival in fluctuating thermal environments, where delayed clearance of misfolded proteins may be more detrimental than modest energetic costs. The observed balance between response time and benefit-to-cost is also relevant in higher organisms, where dysregulation of chaperone networks contributes to aging and neurodegenerative disorders [12,76,77]. In this context, our finding that excessive feedforward activation can paradoxically slow recovery suggests that therapeutic strategies aimed at globally enhancing heat shock factor activity may exhibit nonmonotonic effects unless coordinated with degradation pathways. This is particularly relevant for chaperonopathies, in which mutations or imbalances in chaperone systems impair proteome maintenance [78,79]. Our results imply that improving individual chaperone efficiency alone may not ensure optimal system-level recovery if activation and shutdown dynamics are not properly balanced. While the present framework adopts a coarse-grained representation of the chaperone network, combining multiple chaperone classes into a single effective species, this simplification enables the identification of robust, topology-driven design principles governing HSR regulation. Incorporating additional molecular details may shift the quantitative boundaries of the identified regimes or modulate response amplitudes. However, the emergence of synergistic and trade-off behaviors arises from the fundamental interplay between feedforward activation, sequestration, and degradation, and is therefore expected to remain qualitatively preserved. Accordingly, although quantitative extrapolation to other organisms or translational contexts should be approached with appropriate caution, the core insights are likely to extend broadly across proteostasis systems. Future experimental perturbations of $\sigma_{32}$ translation, sequestration, and degradation strengths could test the predicted regimes and further clarify how regulatory trade-offs shape proteostasis dynamics.

In summary, *E. coli* HSR is not merely sum of its parts; rather, it emerges from a complex interplay of feedforward, sequestration, and degradation. These modules not only cooperate, but also compete to shape overall response in distinct ways. While a higher $\sigma_{32}$ translation can boost folding yield, it may paradoxically slow recovery, an emergent behavior that cannot be predicted by studying individual modules in isolation. Our model provides a quantitative framework for understanding these trade-offs, and its predictions could be experimentally tested by varying the stability of heat shock mRNAs. This approach highlights how the HSR balances speed, efficiency, and adaptability, offering insights relevant for both fundamental biology and the design of synthetic stress-response systems.

## Supporting information

S1 Text Model Validation. (PDF)

S2 Text Comparison of Regulatory Modules under Identical Conditions. (PDF)

S3 Text State-space Representation of Heat Shock Response Dynamics. (PDF)

S4 Text Sensitivity Analysis. (PDF)

S5 Text Robustness of Model Predictions to Aggregation, Unfolding, and Refolding Assumptions. (PDF)

S1 FigHeat Shock Response Dynamics Under Strong Feedforward Control.Time courses of (A) total $\sigma_{32}$ concentration, (B) chaperone production rate, (C) folded protein levels, and (D) benefit-to-cost ratio are plotted for different feedforward strengths ($\eta_{T}$). Here, $\eta_{o}$ is the pre-stress translation rate of $\sigma_{32}$.(TIFF)

S2 FigDelayed Recovery at High Feedforward Strength Despite High Chaperone Levels.Response time is plotted as a function of total chaperone concentration. Dot colors gradient from blue to yellow indicate increasing feedforward strengths ($\eta_{T}$). Response time decreases with increasing chaperone levels. However, at high feedforward strengths ($\eta_{T}$, green to yellow), response time begins to rise again, even when chaperone concentrations are high. The wild-type parameter set are represented by red square.(TIFF)

S3 FigFolding Yield Across Feedforward Strengths.Steady state folding yield are plotted as a function of feedforward strength ($\eta_{T}$). The yield remains nearly constant across the range, indicating 99% recovery of folded proteins. Dot colors gradient from blue to yellow indicate increasing feedforward strengths ($\eta_{T}$).(TIFF)

S4 Fig$\sigma_{32}$ Accumulation at High Feedforward Strength Delays Chaperone Engagement with Misfolded Proteins.Time courses of (A) $\sigma_{32}\cdot C$, (B) M·C, and (C) $\sigma_{32}\cdot C\cdot P$ complex concentrations for varying feedforward strengths ($\eta_{T}$). As $\eta_{T}$ increases, chaperone-$\sigma_{32}$ complexes accumulate rapidly at the onset of heat shock, while chaperone-misfolded protein complexes take longer to form.(TIFF)

S5 FigStronger Sequestration Improves Chaperone Utilization but Slows Recovery.(A) Response time as a function of sequestration strength α, (B) trade-off between response time and benefit-to-cost, and (C) dependence of response time on total chaperone concentration. Dot colors gradient from blue to yellow indicate increasing α values. The wild-type parameter set are represented by red square. Increasing α enhances the benefit-to-cost ratio but slows the response. Sequestration drives the HSR into a trade-off regime where elevated chaperone levels support rapid activation, but the benefit-to-cost ratio is reduced.(TIFF)

S6 FigDegradation of $\sigma_{32}$ Frees Chaperones to Enhance Speed and Efficiency.(A) Response time as a function of degradation strength (β), (B) relationship between response time and benefit-to-cost ratio, (C) response time as a function of total chaperone concentration, (d-f) time evolution of [$\sigma_{32}.C$], [M.C], and [$\sigma_{32}.C.P$], respectively, for varying β. Dot colors (blue to yellow) indicate increasing β values, and the wild-type parameter set is indicated by a red square. Increasing β reduces total chaperone levels. At very low degradation strength, the response time remains low due to high chaperone levels, but exhibits a non-monotonic dependence as β increases. This behavior arises due to timing mismatch between the availability of chaperones for misfolded proteins and their sequestration by $\sigma_{32}$ (D,E). At higher degradation strengths, both the response time and benefit-to-cost ratio improve, indicating a synergistic regime.(TIFF)

S7 FigSequestration Suppresses Baseline $\sigma_{32}$ Activity and Enables Stress-Induced Activation.Time course of chaperone production rate for varying sequestration strength (α). It varies non-monotonically with α. As sequestration of $\sigma_{32}$ increases, baseline production before heat shock decreases, reflecting reduced availability of free $\sigma_{32}$. After heat shock, the peak production rate initially rises with α as sequestered $\sigma_{32}$ is released, driving a rapid transcriptional response. However, at very high α, excessive sequestration restricts free $\sigma_{32}$ too strongly, causing the peak production rate to decline. Chaperone production is maximized at intermediate sequestration strengths, where the balance between suppression at baseline and release upon stress is optimal.(TIFF)

S8 FigEffect of Sequestration Strength on Free $\sigma_{32}$, Misfolded Proteins, and Free Chaperone Availability.(A) Free concentration of $\sigma_{32}$, (B) concentration of misfolded proteins, and (C) free chaperone concentration are plotted for varying sequestration strength (α). At low α, most chaperones are available for folding tasks. As α increases, a larger fraction binds to $\sigma_{32}$ before stress, reducing their availability. During heat stress, free chaperones are consumed to fold misfolded proteins, then rise again as the cell adapts and protein folding is restored. At very high α, excessive sequestration keeps chaperones bound to $\sigma_{32}$ both before and during stress, limiting their availability for misfolded proteins and slowing recovery.(TIFF)

S9 FigImpact of Sequestration Strength and Misfolding Kinetics on Chaperone Production Dynamics.(A) Fold change in chaperone production rate, (B) adaptation time, and (C) response time are plotted as functions of sequestration strength (α) for different misfolding kinetics (γ).(TIFF)

S10 FigFolding Yield Under Varying Sequestration and Feedforward Strengths.Heat map showing folding yield as a function of sequestration strength (α) and feedforward strength ($\eta_{T}$). Folding yield remains high across most of the parameter space but decreases at very high α, particularly when the translation rate of the heat shock factor is low ($\eta_{T}$). The green dot represent the wild-type parameter set.(TIFF)

S11 FigFolding Yield Under Varying Feedforward and Degradation Strengths.Heat map showing folding yield as a function of feedforward strength ($\eta_{T}$) and degradation strength (β). Folding yield decreases at high $\eta_{T}$ when degradation is low, as excess heat shock factors remain bound to chaperones, leaving misfolded proteins unassisted.(TIFF)

S12 FigFolding Yield Under Varying Degradation and Sequestration Strengths.Heat map showing folding yield as a function of degradation strength (β) and sequestration strength (α). Folding yield decreases with increasing α.(TIFF)

S13 FigRobustness of Performance index.Performance index $\eta_{p}$ computed using a recovery threshold of (A) 85% and (B) 90% of pre-heat-shock folded protein levels. Lowering the recovery threshold increases the fraction of parameter space achieving recovery (slight reduction of white regions), as expected. The synergy and trade-off regimes remain robust across threshold definitions.(TIFF)

S14 Fig Mapping Response Time Across HSR Regulatory Parameters. (A) Response time plotted as a function of feedforward, sequestration, and degradation feedback strengths. (B-D) Slice plots showing side and top views of the response time.
(TIFF)

S15 Fig Mapping Benefit-to-Cost Across HSR Regulatory Parameters. (A) Benefit-to-Cost plotted as a function of feedforward, sequestration, and degradation feedback strengths. (B-D) Slice plots showing side and top views of the benefit-to-cost.
(TIFF)

S16 Fig Mapping Adaptation time Across HSR Regulatory Parameters. (A) Adaptation time plotted as a function of feedforward, sequestration, and degradation feedback strengths. (B-D) Slice plots showing side and top views of the adaptation time. Black dot represents the wild-type parameter set.
(TIFF)

S17 FigEffect of ATP Availability on HSR Performance for $K_{A}$ = 0.1.Heat shock response dynamics under varying normalized ATP availability for *K*_A_ = 0.1. (A) Total $\sigma_{32}$, (B) chaperone production rate, (C) folded proteins, (D) benefit-to-cost ratio, (E) fold change in production rate, and (F) response and adaptation times. ATP availability is represented by the normalized variable A, ranging from ATP-limited conditions (A = 0) to saturating ATP levels (A = 1).(TIFF)

S18 FigEffect of ATP Availability on HSR Performance for $K_{A}$ = 0.7.Heat shock response dynamics under varying normalized ATP availability for *K*_A_ = 0.7. (A) Total $\sigma_{32}$, (B) chaperone production rate, (C) folded proteins, (D) benefit-to-cost ratio, (E) fold change in production rate, and (F) response and adaptation times. ATP availability is normalized between limiting (0) and saturating (1) conditions.(TIFF)

S19 FigTrade-off between Response Time and Benefit-to-Cost when Irreversible Aggregation is Included.(A) Concentration of $[\sigma_{32}{]}_{t}$, (D) production rate of chaperones, (B,C) folded proteins, and (E,F) benefit-to-cost are plotted for feedforward (red line), feedforward with sequestration (blue line) and full HSR with feedforward, sequestration, and degradation (magenta line). Heat shock is introduced at time 10 min. The black line in (B,C) corresponds to folded proteins in the absence of chaperones.(TIFF)

S20 FigEffect of Feedforward Strength on Response time when Irreversible Aggregation is Included.Response time calculated for varying feedforward strength ($\eta_{T}$ values) is plotted against $\eta_{T}$ and benefit-to-cost ratio in (A,B), respectively. The red square represents the system at wild-type rate parameters. The gradient from blue to yellow represents increasing $\sigma_{32}$ sequestration with $\eta_{T}$,which also affects the timing of chaperone availability for misfolded proteins.(TIFF)

S21 FigEffect of Sequestration Strength on HSR when Irreversible Aggregation is Included.Fold change in the peak production rate relative to the pre-stress production (A), and adaptation and response times (B) are plotted for varying sequestration strength (α). The red dot corresponds to the wild-type parameter set.(TIFF)

S22 Fig Effect of Irreversible Aggregation on Synergies and Trade-offs in the Regulation of HSR. Heat maps showing the variation in benefit-to-cost (left) and response time (right) across different combinations of feedback strengths: (A,B) feedforward vs. sequestration, (C,D) feedforward vs. degradation, and (E,F) degradation vs. sequestration. Each plot illustrates how pairwise tuning of feedback mechanisms influences system performance during heat shock response. The green dot represents the system at wild-type rate parameters. The white regions show parameter combinations where recovery of folded proteins is below 97%.
(TIFF)

S23 Fig Optimal Regime of Operation when Irreversible Aggregation is Included. (A) Performance index plotted as a function of feedforward, sequestration, and degradation feedback strengths. Slice plots showing side and top views of the performance index are shown in (B), (C), and (D), respectively. The black dot in (C) and (D) corresponds to the wild-type parameter set.
(TIFF)
