# Supplementary material for: Synergies and trade-offs in the heat shock response mechanism
Source: PLoS Comput Biol. 2026 Sep 2;22(9):e1014729. doi: 10.1371/journal.pcbi.1014729 (PMC13577541; doi:10.1371/journal.pcbi.1014729)
Supplement: Supplementary file 1 [file pcbi.1014729.s001.pdf]

S1 Text. Model Validation

Most kinetic rate parameters (k_1, k_{-1}, k_2, k_{-2}, k_3, k_{-3}, k_4, k_5, k_{-5}, β, β_{dil}, a_{trans}) were taken directly from published experimental studies wherever available (Table 1). Protein folding rates were chosen to be similar to those of experimentally characterized malate dehydrogenase, used as a representative proxy for generic misfolding protein substrate. The remaining parameters governing σ_{32} synthesis and chaperone production (η_T and K_σ) were tuned to satisfy the following three calibration targets:

1. The experimentally reported steady-state σ_{32} concentration of approximately 10–50 molecules per cell under basal (non-stress) conditions [1, 2]. Consistent with this range, the model produces a basal σ_{32} concentration of approximately 0.03 μM, corresponding to approximately 30 molecules per cell (Fig. 2a of the main text).

2. The approximately 4-fold elevated post-stress steady-state level of σ_{32}, capturing its transient rise and subsequent decline following heat shock [2, 3].

3. The experimentally observed peak in chaperone production rate within approximately 5 minutes of temperature upshift [3, 4].

Together, these three constraints determined the operating regime of the model. Given this calibration procedure, the following comparisons constitute independent post hoc validation of model behavior.

1. Experimental studies report that following a temperature upshift, σ_{32} increases approximately 10-15 fold within 3–5 minutes before relaxing to a new steady state roughly 3–4 fold above basal levels [2, 3]. The model reproduces both the magnitude of this transient amplification ($\sim$13-fold) and the timing ($\sim$3 min) before relaxing to the steady state (Fig. 2a of the main text).

2. Experimental data report that the chaperone synthesis rate increases approximately 10–12 fold within minutes of heat shock and stabilizes at roughly 2-fold above pre-stress levels [3, 4, 5]. The model reproduces both the transient fold change ($\sim$11-fold) and the long-term steady-state level ($\sim$2-fold above basal) (Fig. 2d of the main text).

3. In addition, we directly compared model trajectories (magenta lines) with independent time-course experimental measurements σ_{32} and chaperone production (black lines) reported by Straus et al. [6] (see Fig. A). The model shows good agreement with the experimental data during both the rapid induction phase and the subsequent adaptation dynamics.

In total, the model was compared against three independent post hoc observations spanning steady-state levels, transient fold changes, and full time-course dynamics. Together with the calibration targets described above, these results demonstrate that, despite its coarse-grained structure, the model reproduces key experimentally observed features of the heat shock response.

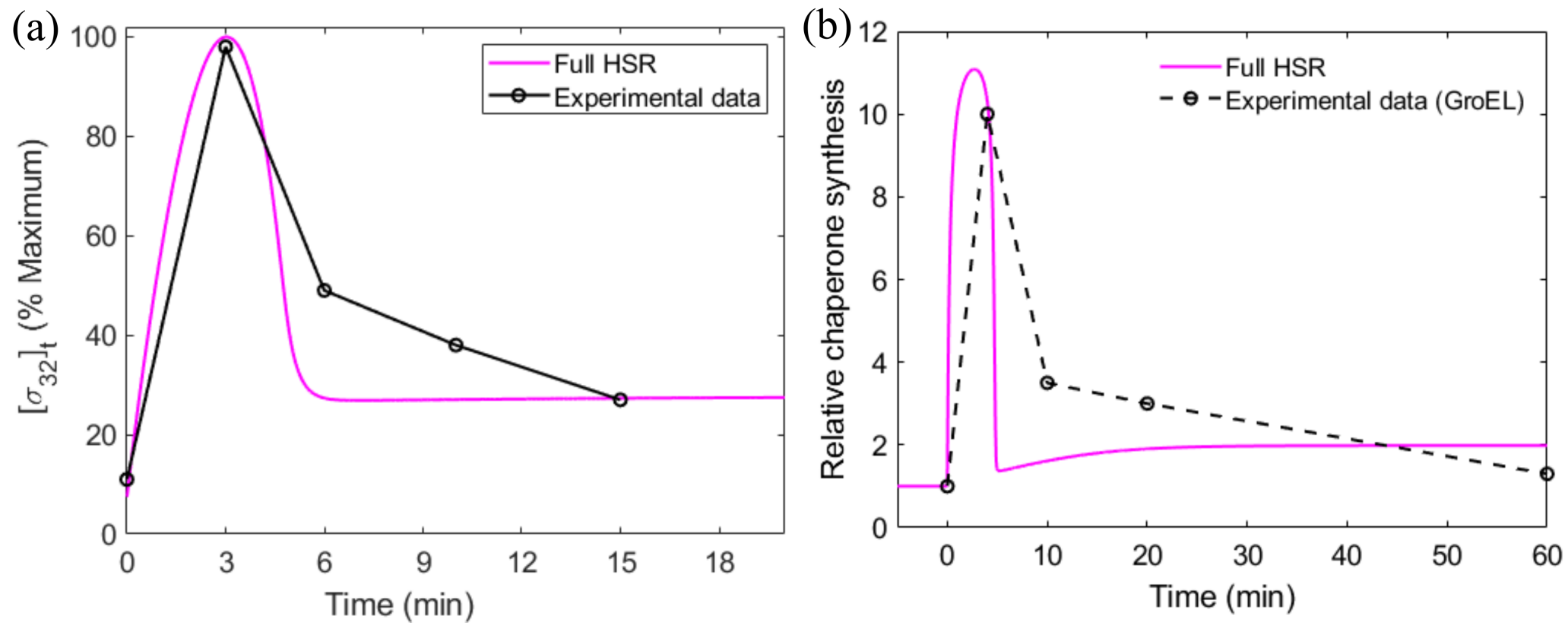

Figure A: Comparison of model predictions with experimental data for (a) σ_{32} and (b) chaperone synthesis during heat shock. Model trajectories (magenta curve) closely match experimental time-course measurements (black curve) from [6], reproducing both the magnitude and recovery kinetics of the heat shock response.

References

[1] Elizabeth A Craig and Carol A Gross. "Is hsp70 the cellular thermometer?" In: *Trends in biochemical sciences* 16 (1991), pp. 135–140.

[2] LYNN Connolly, TAKASHI Yura, and CAROL A Gross. "Autoregulation of the heat shock response in procaryotes". In: *Molecular Chaperones and Folding Catalysts Regulation, Cellular Function and Mechanism* (1999), pp. 13–33.

[3] David B Straus, William A Walter, and Carol A Gross. "The heat shock response of E. coli is regulated by changes in the concentration of σ32". In: *Nature* 329.6137 (1987), pp. 348–351.

[4] T Yamamori et al. "Transient regulation of protein synthesis in Escherichia coli upon shift-up of growth temperature". In: *Journal of Bacteriology* 134.3 (1978), pp. 1133–1140.

[5] Peggy G Lemaux et al. "Transient rates of synthesis of individual polypeptides in E. coli following temperature shifts". In: *Cell* 13.3 (1978), pp. 427–434.

[6] David Straus, William Walter, and Carol A Gross. "DnaK, DnaJ, and GrpE heat shock proteins negatively regulate heat shock gene expression by controlling the synthesis and stability of sigma 32." In: *Genes & Development* 4.12a (1990), pp. 2202–2209.
