# Supplementary material for: Synergies and trade-offs in the heat shock response mechanism
Source: PLoS Comput Biol. 2026 Sep 2;22(9):e1014729. doi: 10.1371/journal.pcbi.1014729 (PMC13577541; doi:10.1371/journal.pcbi.1014729)
Supplement: Supplementary file 2 [file pcbi.1014729.s002.pdf]

S2 Text. Comparison of Regulatory Modules under Identical Conditions

For completeness, we also compared the feedback modules by using the same kinetic parameters across all three modules (see Fig. A). Under these conditions, the feedforward-only module (red line) shows only a minimal drop in folded proteins but lacks recovery dynamics (Fig. Ab). The feedforward with sequestration module (blue line) shows a relatively larger drop followed by rapid recovery. Both of these reduced modules appear to outperform the full HSR module (magenta line) in terms of recovery. However, this apparent improvement is accompanied by very high σ_{32} levels, exceeding experimentally reported ranges by orders of magnitude (Fig. Aa). In addition, chaperone efficiency (η_{bc}) is significantly reduced in both the reduced modules (Fig. Ad), indicating inefficient utilization of chaperone pool (Fig. Ac). Thus, the faster recovery arises from excessive accumulation of σ_{32} and chaperones rather than a superior feedback architecture

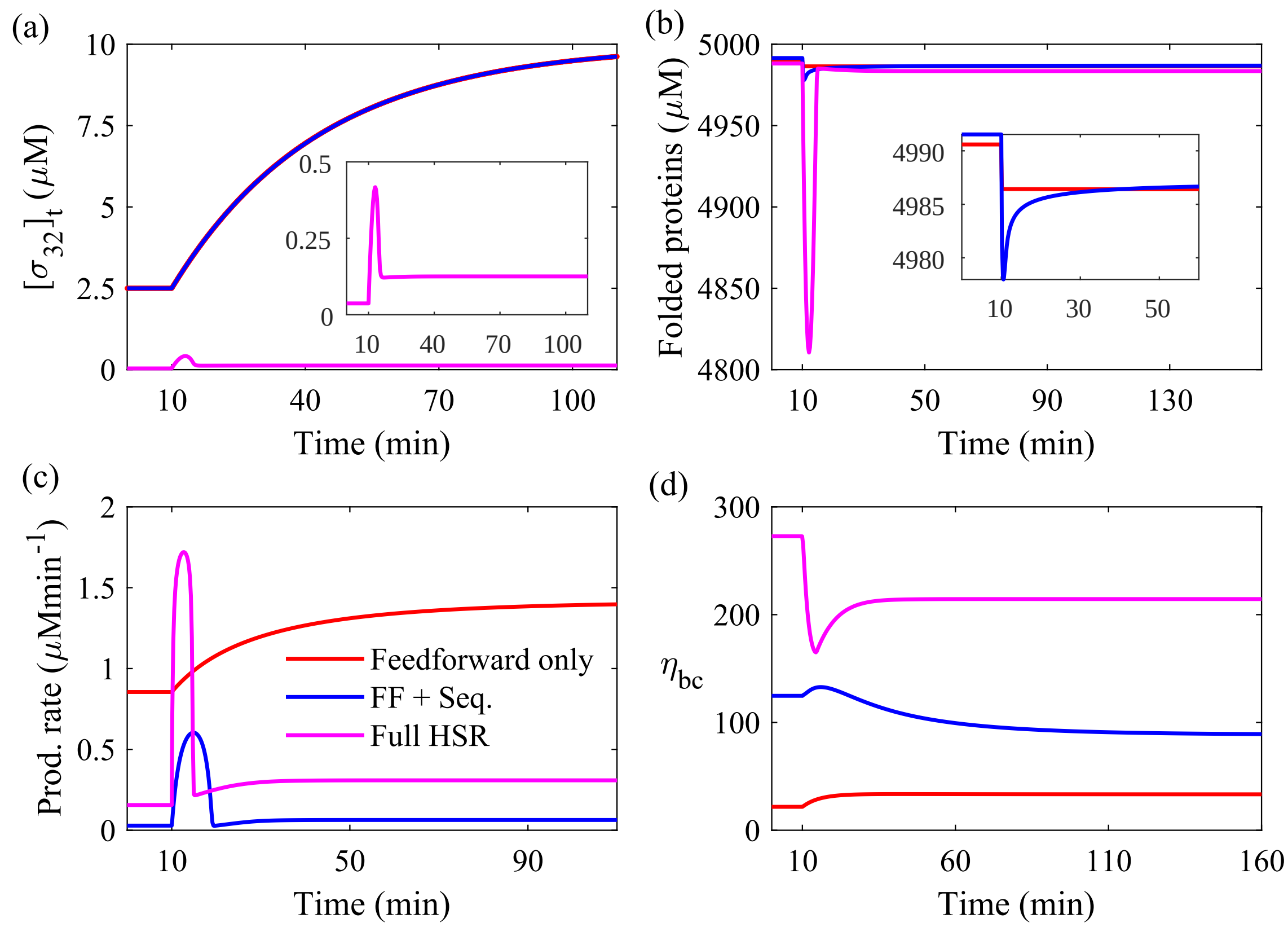

Figure A: (a) Total σ_{32} concentration, (b) folded protein levels, (c) production rate of chaperones, and (d) benefit-to-cost ratio (η_{bc}) are plotted as a function of time for the feedforward-only (red), feedforward and sequestration (blue), and full HSR module (magenta). In this comparison, translation parameters were not recalibrated to match pre-heat-shock steady states. These results illustrate that apparent improvements in response speed can arise from baseline shifts rather than genuine architectural advantages.
