# Supplementary material for: Synergies and trade-offs in the heat shock response mechanism
Source: PLoS Comput Biol. 2026 Sep 2;22(9):e1014729. doi: 10.1371/journal.pcbi.1014729 (PMC13577541; doi:10.1371/journal.pcbi.1014729)
Supplement: Supplementary file 3 [file pcbi.1014729.s003.pdf]

S3 Text. State-space representation of heat shock response dynamics

To provide an intuitive visualization of system dynamics, we analyzed the trajectories of the heat shock response in state space. Specifically, trajectories were plotted in the three-dimensional concentration space defined by total σ_{32}, chaperones, and protease, with folded protein levels represented by a color gradient (Fig. Aa-c). Heat shock is introduced at t = 10 min, and colored rings indicate selected time points along the trajectory (red: t = 10 min; green: t=15 min; blue: t=110 min), illustrating the temporal progression of the response.

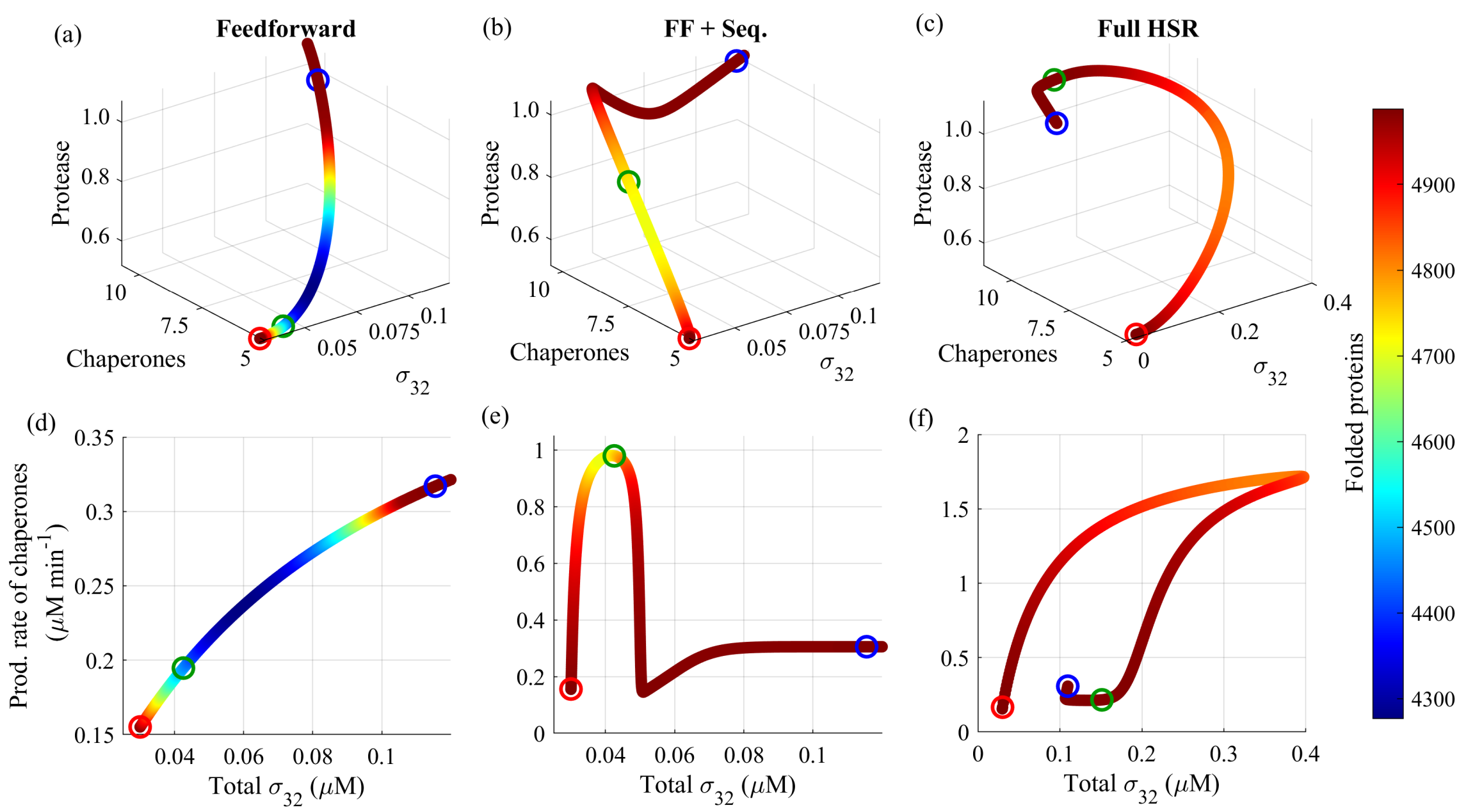

Figure A: (a–c) Three-dimensional trajectories in (σ_{32}, chaperone, protease) concentration space for the feedforward-only, feedforward with sequestration and full HSR modules. (d–f) Corresponding two-dimensional state-space projections of chaperone production rate versus total σ_{32}. Each point represents the system state following heat shock, with folded protein levels encoded by color. Heat shock is introduced at time 10 min. Colored rings indicate specific time stamps (t = 10 min, 15 min, and 110 min), highlighting the transient progression and subsequent relaxation toward steady state. The temporal markers reveal differences in trajectory curvature and recovery speed across architectures.

All three regulatory architectures converge to comparable steady-state levels; however, they differ markedly in how they traverse concentration space during heat shock. In the feedforward-only module, the trajectory progresses monotonically towards the steady-state (Fig. Aa). The green ring (5 min after heat shock) lies close to the red ring, indicating relatively slow movement through state space during the early activation phase. In the feedforward with sequestration module, the trajectory shows a larger displacement, particularly in chaperone and protease concentrations, before approaching steady state (Fig. Ab). The green ring appears at an intermediate position along the trajectory, reflecting a faster early response compared to feedforward-only module. In contrast, the full HSR architecture follows a curved trajectory that bends back toward baseline without fully returning to the it (Fig. Ac). σ_{32} levels initially rise higher than in the other modules and subsequently decrease due to regulatory feedback. Here, the green ring lies substantially farther along the trajectory and closer to the blue ring (100 min post-heat shock), indicating faster

progression through concentration space. The full HSR also maintains higher folded protein levels relative to the other architectures.

To better visualize how the regulatory architectures differ in their transient navigation of concentration space, we project the dynamics onto a reduced plane defined by chaperone production rate versus total σ_{32} (Fig. Ad-f), again encoding folded protein levels by color. This representation preserves the key qualitative features of the trajectories while making differences between regulatory architectures more apparent. In this space, again, the feedforward-only module exhibits a predominantly monotonic trajectory toward an elevated steady state, without reversal toward baseline (Fig. Ad). The feedforward with sequestration module displays a transient increase in chaperone production followed by a decline, and subsequently relaxes to a steady state that remains above baseline (Fig. Ae). The full HSR architecture follows a loop-like path characterized by rapid activation followed by adaptation toward a new steady state (Fig. Af). Although it bends back toward the pre-stress value, it does not fully return to the original steady state, reflecting the sustained chaperone demand imposed by elevated temperature. The relative positioning of the green ring mirrors that observed in the three-dimensional state space, indicating consistent progression across representations.

These representations highlight that differences between regulatory architectures arise primarily from transient dynamics and the manner in which the system navigates concentration space, rather than from differences in steady-state behavior.
