# Supplementary material for: Synergies and trade-offs in the heat shock response mechanism
Source: PLoS Comput Biol. 2026 Sep 2;22(9):e1014729. doi: 10.1371/journal.pcbi.1014729 (PMC13577541; doi:10.1371/journal.pcbi.1014729)
Supplement: Supplementary file 4 [file pcbi.1014729.s004.pdf]

S4 Text. Sensitivity Analysis

To assess the robustness of the model predictions to parameter uncertainty, we first performed a one-at-a-time sensitivity analysis, that quantifies the influence of individual parameters and identifies whether a single parameter critically affects the model predictions. Because the HSR is governed by multiple interacting nonlinear processes, parameter variations may occur simultaneously, and such interactions cannot be captured by one-at-a-time perturbations alone. We therefore complemented this analysis with a multi-parameter perturbation analysis using Latin Hypercube Sampling (LHS) [1, 2].

1. One-at-a-time Sensitivity Analysis

We performed a detailed sensitivity analysis by independently varying the three core regulatory parameters: feedforward translation strength (η_T), sequestration strength (α) and degradation strength (β). Each parameter was varied over an extended range from -100% to +100% relative to the baseline (wild-type), while keeping all other parameters fixed. For each perturbation, we computed key model outputs, including response time, adaptation time, steady-state σ_{32}, total chaperone levels, folded protein levels, and the benefit-to-cost ratio.

The results of these parameter variations are summarized in Fig. A, where each output is plotted as a function of relative parameter change. This allows a comparison of how individual regulatory modules influence different aspects of the heat shock response.

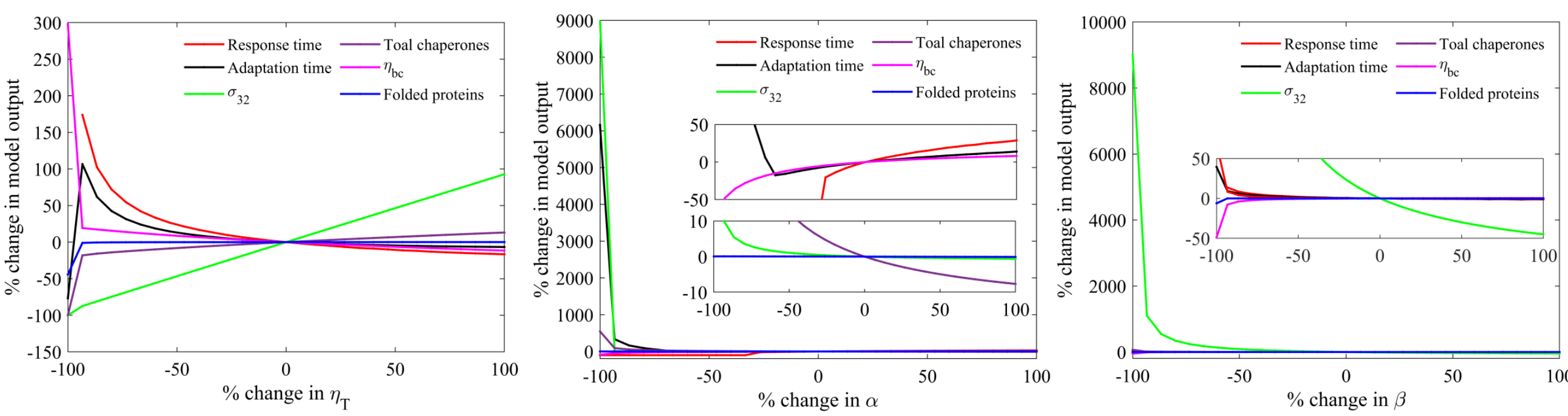

Figure A: Percentage change in model outputs as a function of percentage perturbation in (a) feedforward translation strength (η_T), (b) sequestration strength (α), and (c) degradation rate (β). Outputs include response time, adaptation time, steady-state σ_{32}, total chaperones, folded proteins, and benefit-to-cost ratio (η_{bc}).

Variations in feedforward strength (η_T) primarily affect steady-state σ_{32} levels, reflecting its direct role in synthesis, while having relatively limited impact on folded protein levels. Changes in sequestration strength (α) regulate the availability of free σ_{32}, thereby influencing the magnitude of chaperone production. Strong reductions in α lead to elevated free σ_{32}, resulting in increased chaperone production and delayed adaptation due to slower shutdown. Variations in degradation strength (β) predominantly affect σ_{32} levels, with comparatively modest effects on other outputs. Across the explored parameter range, model outputs vary smoothly without abrupt transitions or oscillatory dynamics, and the system consistently converges to a steady state. Extreme parameter reductions (approaching -100%) lead to expected biological responses, such as accumulation of σ_{32} and delayed recovery, rather than numerical instability.

Overall, this analysis indicates that the model behavior is robust to small perturbations in key regulatory parameters, and that the observed system dynamics are not sensitive to precise parameter values.

2. Multi-parameter Perturbation Analysis

Although the one-at-a-time analysis demonstrates that model outputs vary smoothly and robustly under individual parameter perturbations, it does not capture the effects of simultaneous variation of multiple parameters. To address this limitation, we performed an additional multi-parameter perturbation analysis using Latin Hypercube Sampling [1, 2, 3], which simultaneously varies multiple parameters and provides efficient, approximately uniform coverage of the multidimensional parameter space.

Specifically, we simultaneously perturbed the sequestration strength (α), degradation strength (β), protein folding rate (k_{uf}), and aggregation rate (k_{agg}) using Latin Hypercube Sampling. LHS was employed because it provides efficient and approximately uniform coverage of the multidimensional parameter space while requiring substantially fewer simulations than exhaustive sampling. Two perturbation ranges were considered, with parameters varied independently within $\pm25\%$ and $\pm50\%$ of their reference values, generating 500 parameter sets for each case.

To examine the robustness of feedforward regulation, we varied the feedforward strength (η_T) over a broad range (0.05–9 μM min^{-1}) for every sampled parameter set. This resulted in 500 response-time trajectories for each perturbation range. As shown in Fig. B, the characteristic nonmonotonic dependence of response time on feedforward strength was consistently preserved under both $\pm25\%$ (Fig. Ba) and $\pm50\%$ (Fig. Bb) perturbations. While simultaneous parameter variation especially $\pm50\%$ perturbations broadened the distribution of response times and increased quantitative variability, particularly at very low and very high feedforward strengths, the minimum response-time region remained confined to essentially the same intermediate range of η_T. Importantly, the wild-type parameter set remained within this high-performance region for both perturbation ranges.

Similarly, to examine the robustness of sequestration, we varied the sequestration strength (α) over the range 10^{-2}-10^{2}, while simultaneously perturbing the feedforward strength (η_T), degradation strength (β), protein folding rate (k_{uf}), and aggregation rate (k_{agg}) using LHS. As shown in Fig. C, the characteristic dependence of the fold change in chaperone production, adaptation time, and response time on sequestration strength was preserved across both perturbation ranges. Although the numerical values and the spread of the trajectories increased under larger perturbations, the qualitative system behavior remained unchanged. Specifically, moderate sequestration continued to balance rapid stress adaptation with efficient chaperone utilization, whereas both weak and excessively strong sequestration degraded overall performance. These results demonstrate that the predicted regulatory role of sequestration is robust to correlated multi-parameter uncertainty.

Finally, because our results predict that degradation acts synergistically with feedforward activation and sequestration to improve HSR performance, we examined whether this conclusion remains robust under correlated multi-parameter uncertainty. To this end, we varied the degradation strength by one order of magnitude in both directions and repeated the above analyses in Figs. B and C. For the reduced degradation case, the parameters were perturbed by $\pm25\%$ (Fig. Da-c) and $\pm50\%$(Fig. Dd-f). For the enhanced degradation case, the parameters were perturbed within $\pm50\%$ of their reference values (Fig. Dg-i). Across all cases, the characteristic feedforward and sequestration response patterns remained preserved. As expected, reduced degradation delayed

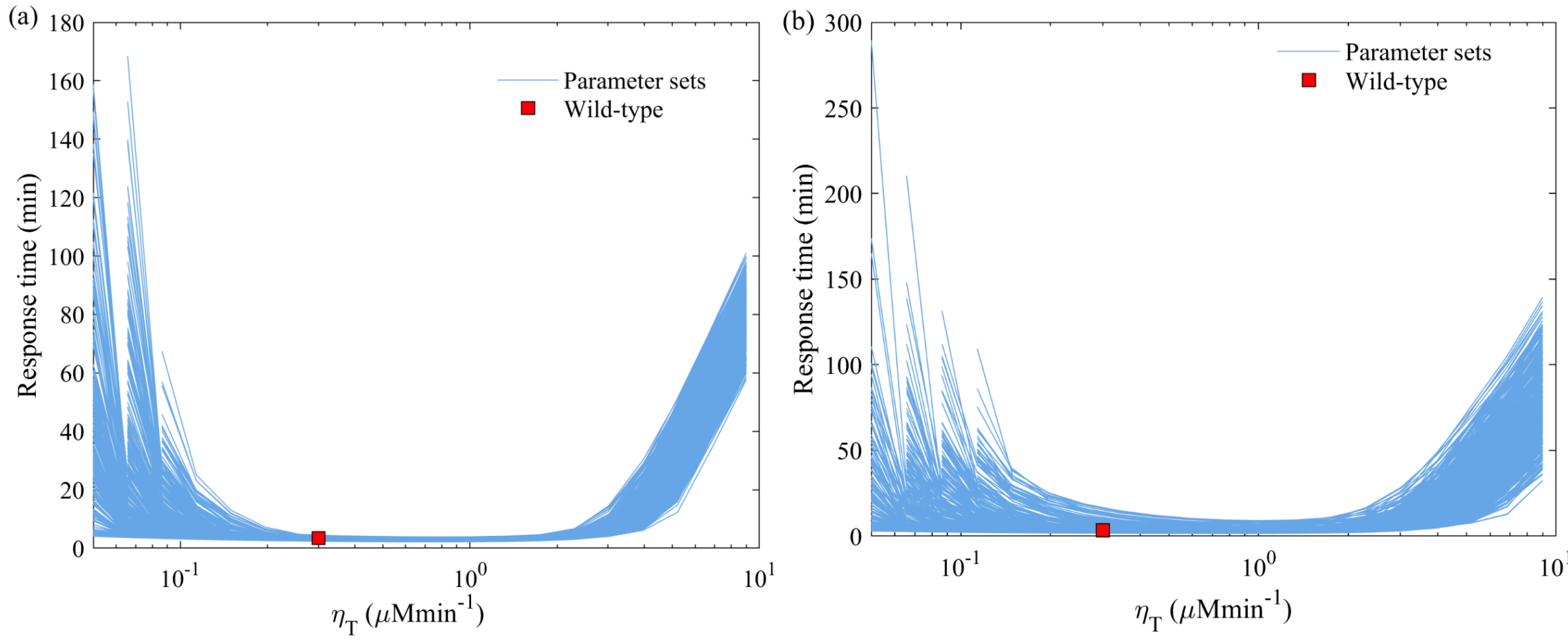

Figure B: Robustness of feedforward regulation under correlated multi-parameter perturbations: Response time as a function of feedforward strength (η_T) obtained by simultaneously perturbing the sequestration strength (α), degradation strength (β), protein folding rate (k_{uf}), and aggregation rate (k_{agg}) using Latin Hypercube Sampling (LHS). The interacting parameters were varied within (a) $\pm 25\%$ and (b) $\pm 50\%$ of their reference values to generate 500 parameter sets. For each sampled parameter set, the feedforward strength (η_T) was independently varied, and the corresponding response time was computed. Blue curves represent the response-time trajectories for all sampled parameter sets, while the red square denotes the wild-type parameter set.

recovery, increased response time variability and, particularly under $\pm 50\%$ perturbations, due to slower σ_{32} removal and reduced chaperone recycling. Conversely, stronger degradation accelerated recovery, reduced variability, and broadened the parameter region over which efficient HSR operation was achieved. Importantly, these changes affected the quantitative performance of the system without altering the underlying regulatory relationships, demonstrating that the synergistic role of degradation and is not a consequence of a particular parameter combination.

Taken together, these analyses demonstrate that correlated multi-parameter perturbations primarily influence the extent of the high-performance region, while preserving the qualitative conclusions identified in this study. Specifically, the nonmonotonic dependence on feedforward activation and sequestration, the existence of an intermediate operating regime that balances response speed and resource utilization, and the synergistic contribution of degradation all remain robust under simultaneous variation of multiple interacting kinetic parameters.

References

[1] Jon C Helton and Freddie Joe Davis. "Latin hypercube sampling and the propagation of uncertainty in analyses of complex systems". In: *Reliability Engineering & System Safety* 81.1 (2003), pp. 23–69.

[2] Michael D McKay, Richard J Beckman, and William J Conover. "A comparison of three methods for selecting values of input variables in the analysis of output from a computer code". In: *Technometrics* 42.1 (2000), pp. 55–61.

[3] Simeone Marino et al. "A methodology for performing global uncertainty and sensitivity analysis in systems biology". In: *Journal of theoretical biology* 254.1 (2008), pp. 178–196.

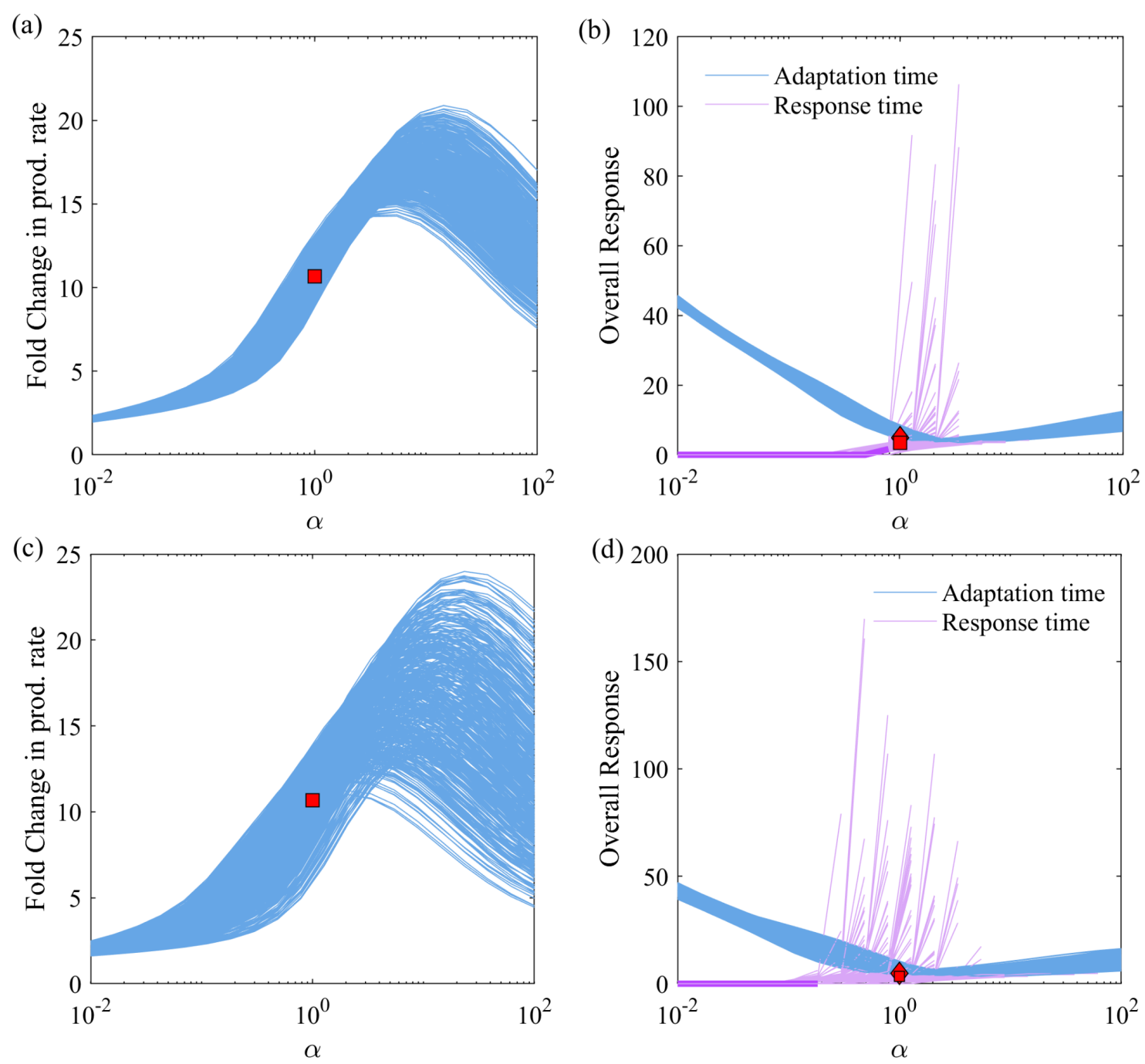

Figure C: Robustness of sequestration regulation under correlated multi-parameter perturbations: The feedforward strength (η_T), degradation strength (β), protein folding rate (k_{uf}), and aggregation rate (k_{agg}) were simultaneously perturbed using Latin Hypercube Sampling (LHS) within (a,b) ±25% and (c,d) ±50% of their reference values to generate 500 parameter sets. For each sampled parameter set, the sequestration strength (α) was independently varied over the range 10^{-2}–10^{2}, and the corresponding fold change in chaperone production, adaptation time (blue), and response time (magenta) were computed. Panels (a,c) show the fold change in chaperone production, while panels (b,d) show the corresponding adaptation time and response time. Blue and magenta curves represent the sampled parameter sets, whereas the red square denotes the wild-type parameter set.

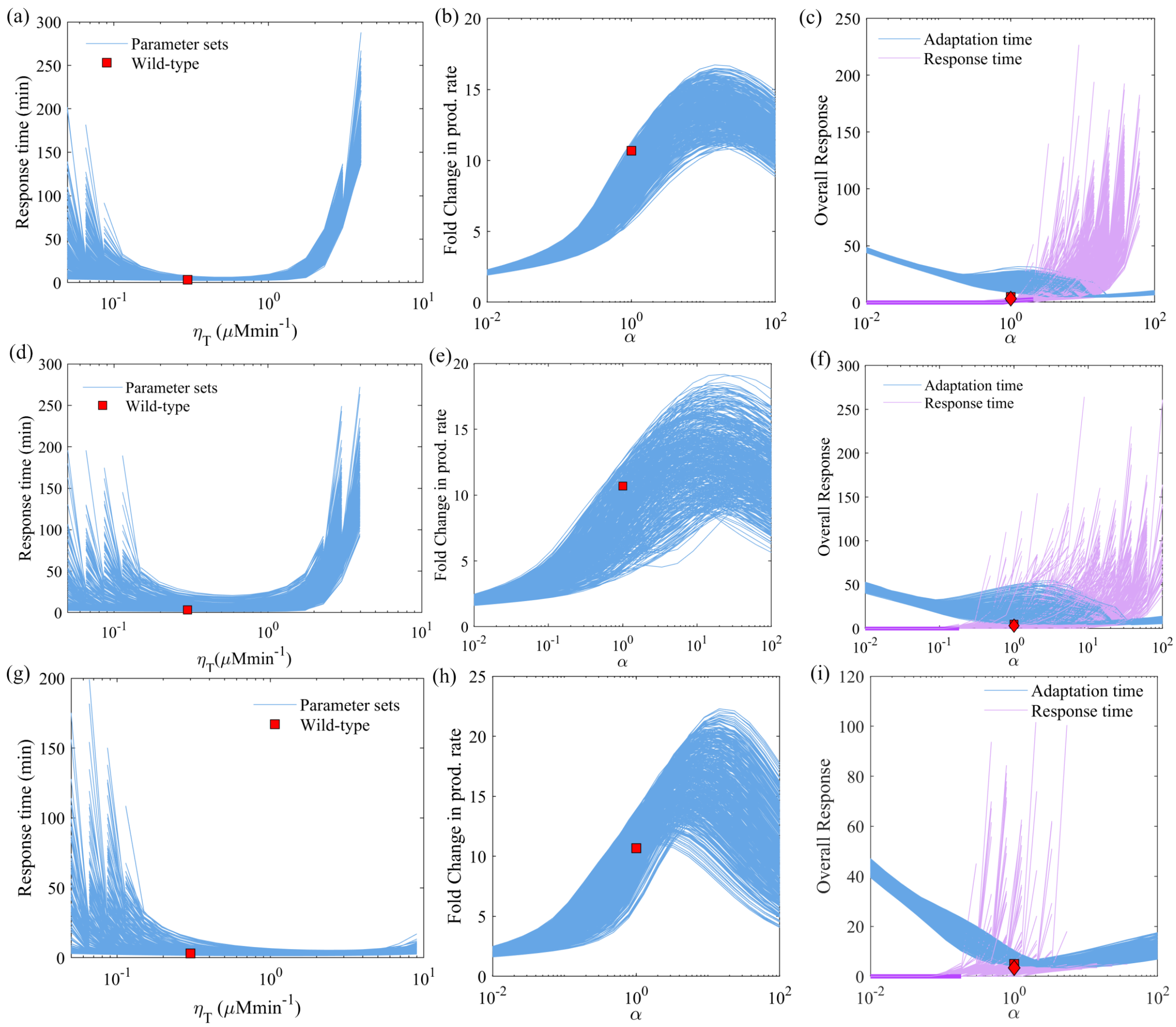

Figure D: Robustness of the synergistic role of degradation under correlated multi-parameter perturbations: The multi-parameter perturbation analyses shown in Figs. B and C were repeated after modifying the degradation strength (β). The degradation strength was decreased by one order of magnitude relative to its reference value in (a-f) and increased by one order of magnitude in (g-i). For the reduced degradation case, the parameters were perturbed within $\pm 25\%$ (a-c) and $\pm 50\%$ (d-f) of their reference values. For the enhanced degradation case, the parameters were perturbed within $\pm 50\%$ of their reference values (g-i). The red squares denote the wild-type parameter set.
