# Supplementary material for: Synergies and trade-offs in the heat shock response mechanism
Source: PLoS Comput Biol. 2026 Sep 2;22(9):e1014729. doi: 10.1371/journal.pcbi.1014729 (PMC13577541; doi:10.1371/journal.pcbi.1014729)
Supplement: Supplementary file 5 [file pcbi.1014729.s005.pdf]

S5 Text. Robustness of Model Predictions to Aggregation, Unfolding, and Refolding Assumptions.

To examine robustness of the model predictions to a wider range of aggregation and refolding parameters, we have now performed additional analyses by independently varying the aggregation rate (k_{agg}), unfolded-protein influx, and refolding rate by ten-fold above and below their reference values. Here, we examine whether these perturbations preserve (i) the nonmonotonic dependence of response time on feedforward strength (η_T), (ii) the nonmonotonic dependence of fold change and adaptation time on sequestration strength (α), and (iii) the existence and location of the high-performance operating region in the performance landscape.

1. Robustness to variation in aggregation rate:

When the aggregation rate was reduced ten-fold, the proteotoxic damage became less severe, resulting in faster recovery and a larger region of parameter space capable of achieving 97% folded-protein recovery. Nevertheless, the qualitative conclusions remained unchanged (Figs. 1 - 4). Fig. 1 shows that the full HSR module continued to outperform the reduced regulatory architectures. Consistent with our findings, Fig. 2 demonstrates nonmonotonic dependence of response time on the feedforward strength, while Fig. 3 confirms that sequestration continued to exhibit its dual regulatory role by modulating fold change, response time, and adaptation time. Finally, Fig. 4 shows that qualitative structure of the performance landscape remains unchanged, with only quantitative expansions of the operating regime.

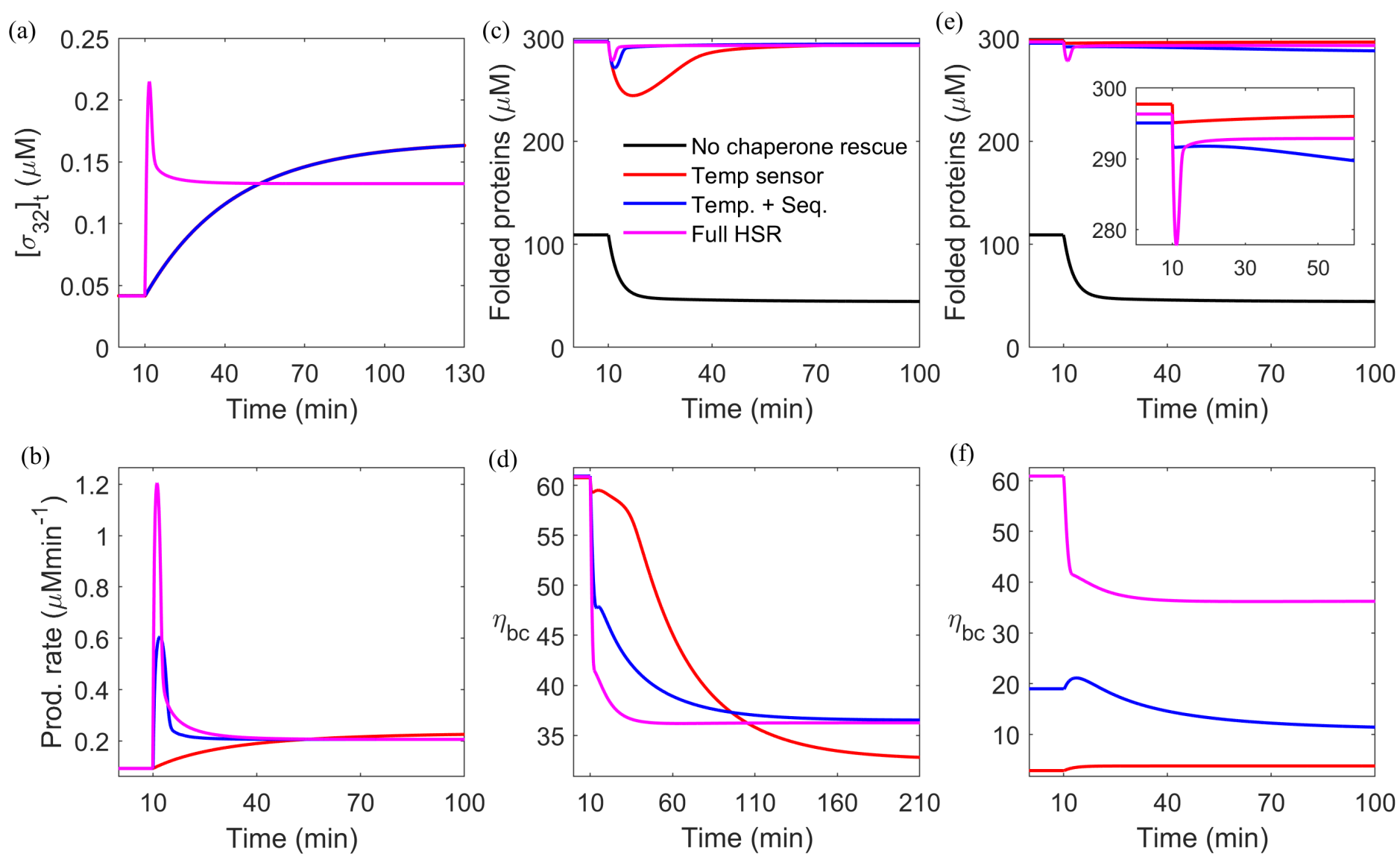

Figure 1: Heat shock response when aggregation is reduced ten-fold: (a) Concentration of $[\sigma_{32}]_t$, (b) production rate of chaperones, (c,e) folded proteins, and (d,f) benefit-to-cost are plotted for feedforward (red line), feedforward with sequestration (blue line) and full HSR with feedforward, sequestration, and degradation (magenta line). Heat shock is introduced at time 10 min. The black line in (b,c) corresponds to folded proteins in the absence of chaperones. For panels (a–d), pre–heat shock steady-state levels were matched across all modules. Subsequently, the translation rates were increased in the reduced modules to match the recovery dynamics of the full HSR, and the resulting behavior is shown in panels (e,f).

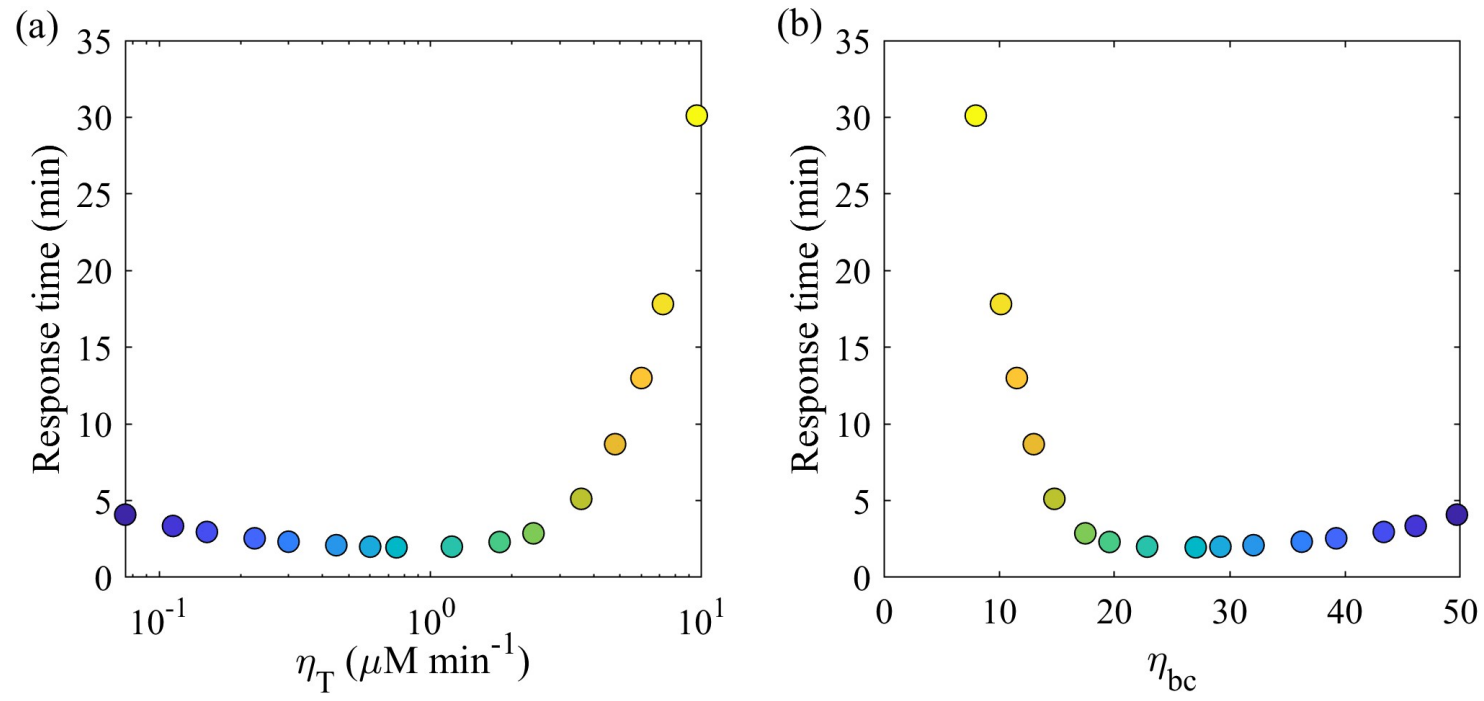

Figure 2: Heat shock response when aggregation is reduced ten-fold: Response time for varying feedforward strength (η_T values) is plotted against η_T and benefit-to-cost ratio in (a,b), respectively. The gradient from blue to yellow represents increasing σ_{32} sequestration with η_T, which also affects the timing of chaperone availability for misfolded proteins.

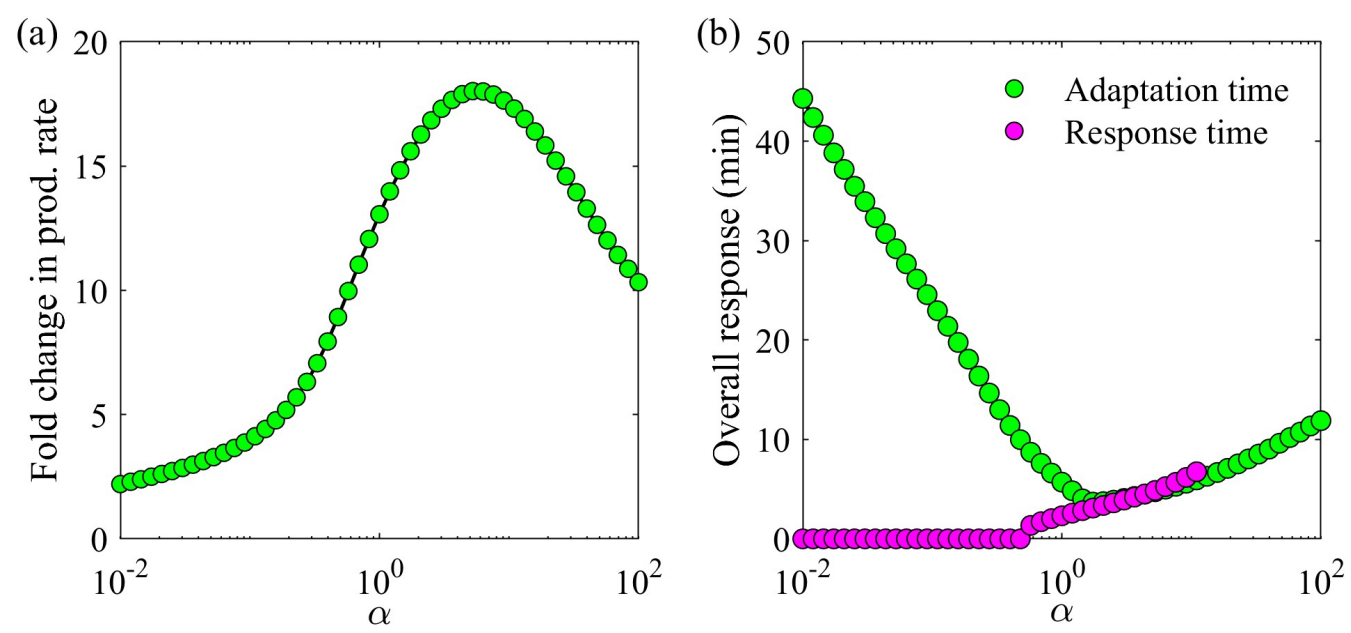

Figure 3: Heat shock response when aggregation is reduced ten-fold: (a) Fold change in the peak production rate relative to the pre-stress production, and (b) adaptation and response times are plotted for varying sequestration strength (α).

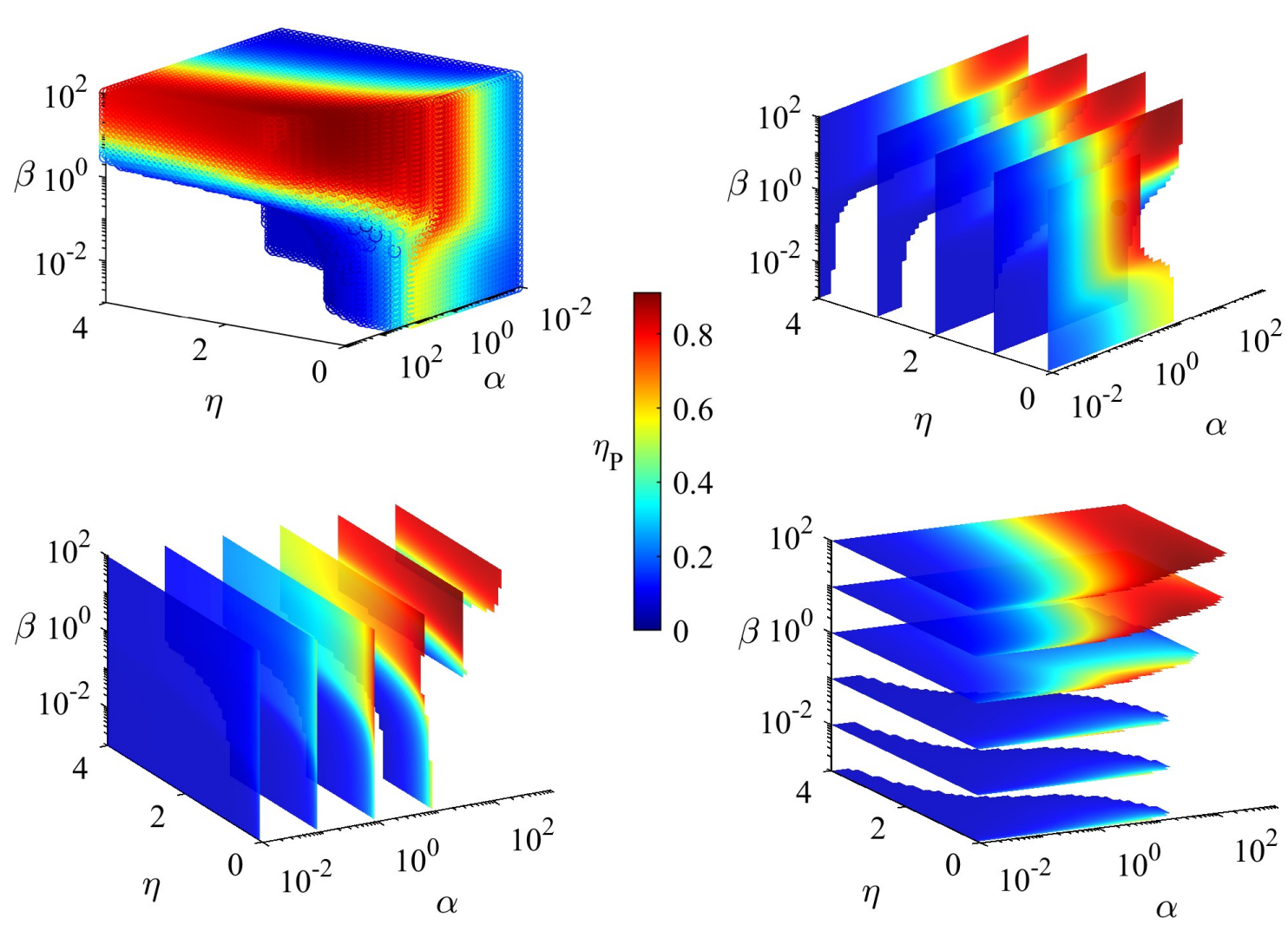

Figure 4: Heat shock response when aggregation is reduced ten-fold: (a) Performance index plotted as a function of feedforward, sequestration, and degradation feedback strengths. Slice plots showing side and top views of the performance index are shown in (b), (c), and (d), respectively.

Conversely, increasing the aggregation rate by one order of magnitude imposed a substantially more severe proteotoxic burden. As expected, folded-protein recovery became more difficult, the parameter space capable of achieving 97% folded proteins recovery reduced, and the maximum attainable performance index was reduced (Figs. 5 - 8). These changes arise from the increased accumulation of irreversibly aggregated proteins, which limits the ability of the HSR to restore the folded-protein pool. Despite these quantitative changes, the central conclusions again remained unchanged. The trade-off between response time and benefit-to-cost ratio across different modules persisted (Fig. 5), Fig. 6 shows that excessive feedforward activation still produced paradoxical slowing of recovery, while Fig. 7 confirms that sequestration retained the same qualitative influence on fold change and adaptation. Fig. 8 further demonstrates that, although severe aggregation substantially restricts the high-performance operating regime, increasing degradation strength partially restores this region by promoting more efficient recovery. However, compared to the extended model of the main text (with experimentally motivated rates), achieving an optimal response now requires stronger feedforward activation and sequestration in addition to high degradation, reflecting the greater regulatory effort needed to counter the increased aggregation burden.

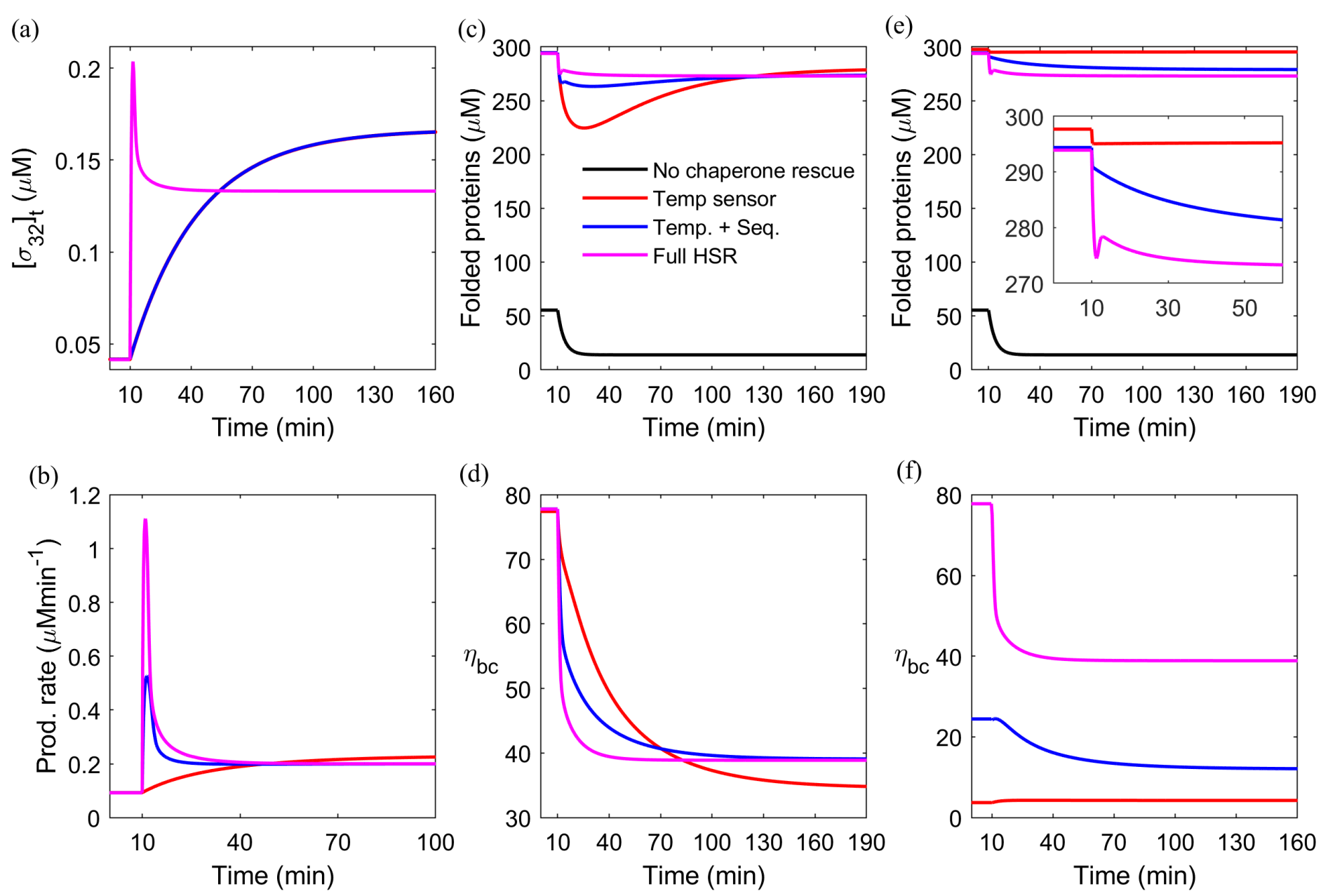

Figure 5: Heat shock response when aggregation is increased ten-fold: (a) Concentration of $[\sigma_{32}]_t$, (b) production rate of chaperones, (c,e) folded proteins, and (d,f) benefit-to-cost are plotted for feedforward (red line), feedforward with sequestration (blue line) and full HSR with feedforward, sequestration, and degradation (magenta line). Heat shock is introduced at time 10 min. The black line in (b,c) corresponds to folded proteins in the absence of chaperones. For panels (a–d), pre–heat shock steady-state levels were matched across all modules. Subsequently, the translation rates were increased in the reduced modules to match the recovery dynamics of the full HSR, and the resulting behavior is shown in panels (e,f).

Collectively, these analyses demonstrate that altering the aggregation rate primarily changes the severity of proteotoxic stress and therefore the quantitative values of the performance metrics, while the mechanistic conclusions regarding the interplay between feedforward activation, sequestration, and degradation remain robust over a broad range of aggregation strengths.

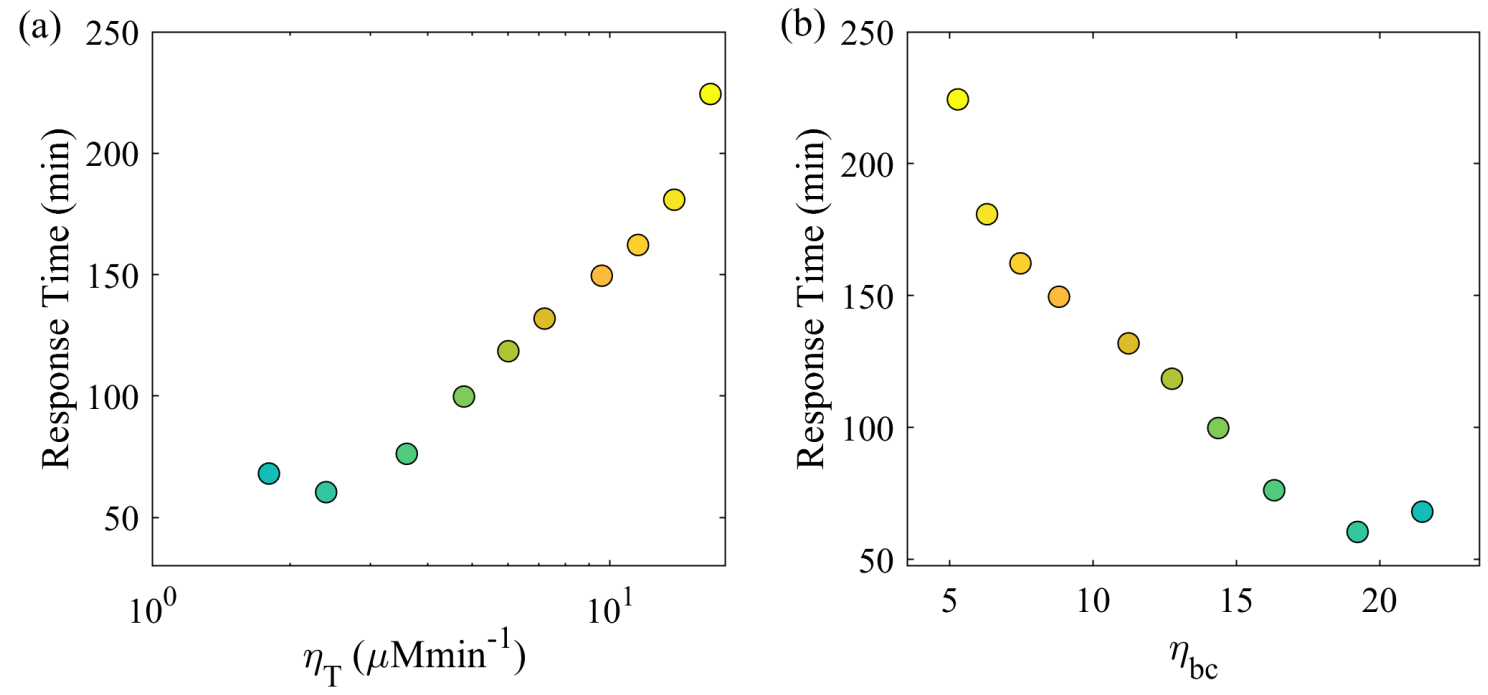

Figure 6: Heat shock response when aggregation is increased ten-fold: Response time for varying feedforward strength (η_T values) is plotted against η_T and benefit-to-cost ratio in (a,b), respectively. The gradient from blue to yellow represents increasing σ_{32} sequestration with η_T, which also affects the timing of chaperone availability for misfolded proteins.

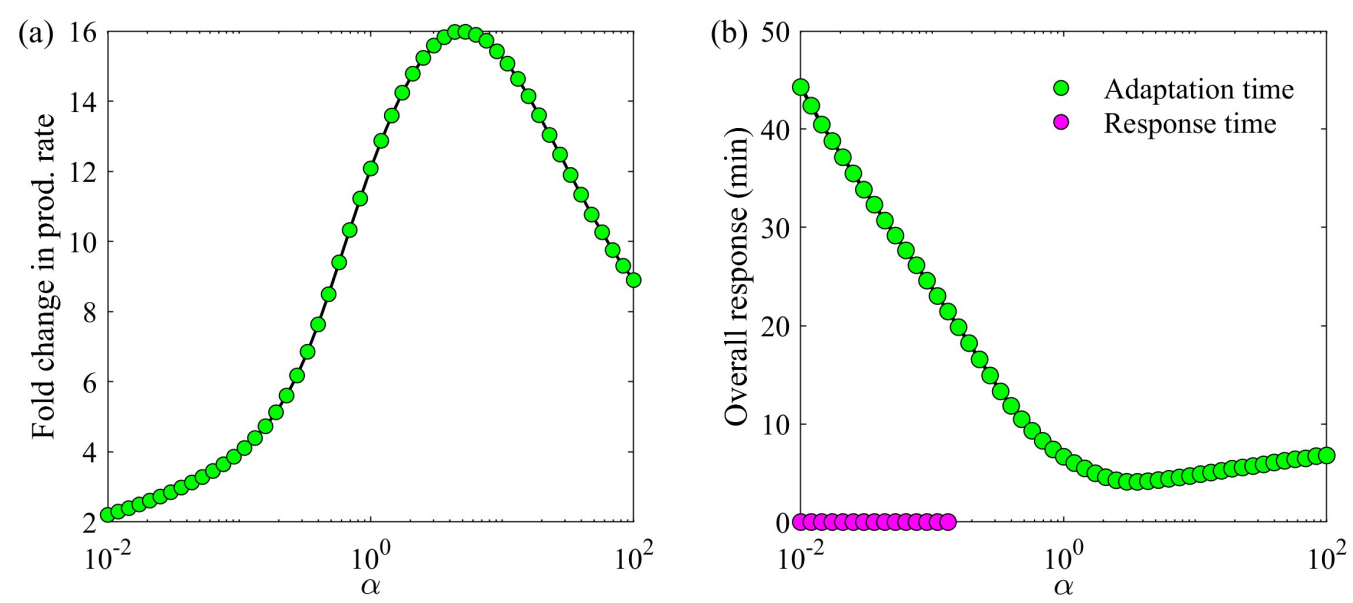

Figure 7: Heat shock response when aggregation is increased ten-fold: (a) Fold change in the peak production rate relative to the pre-stress production, and (b) adaptation and response times are plotted for varying sequestration strength (α).

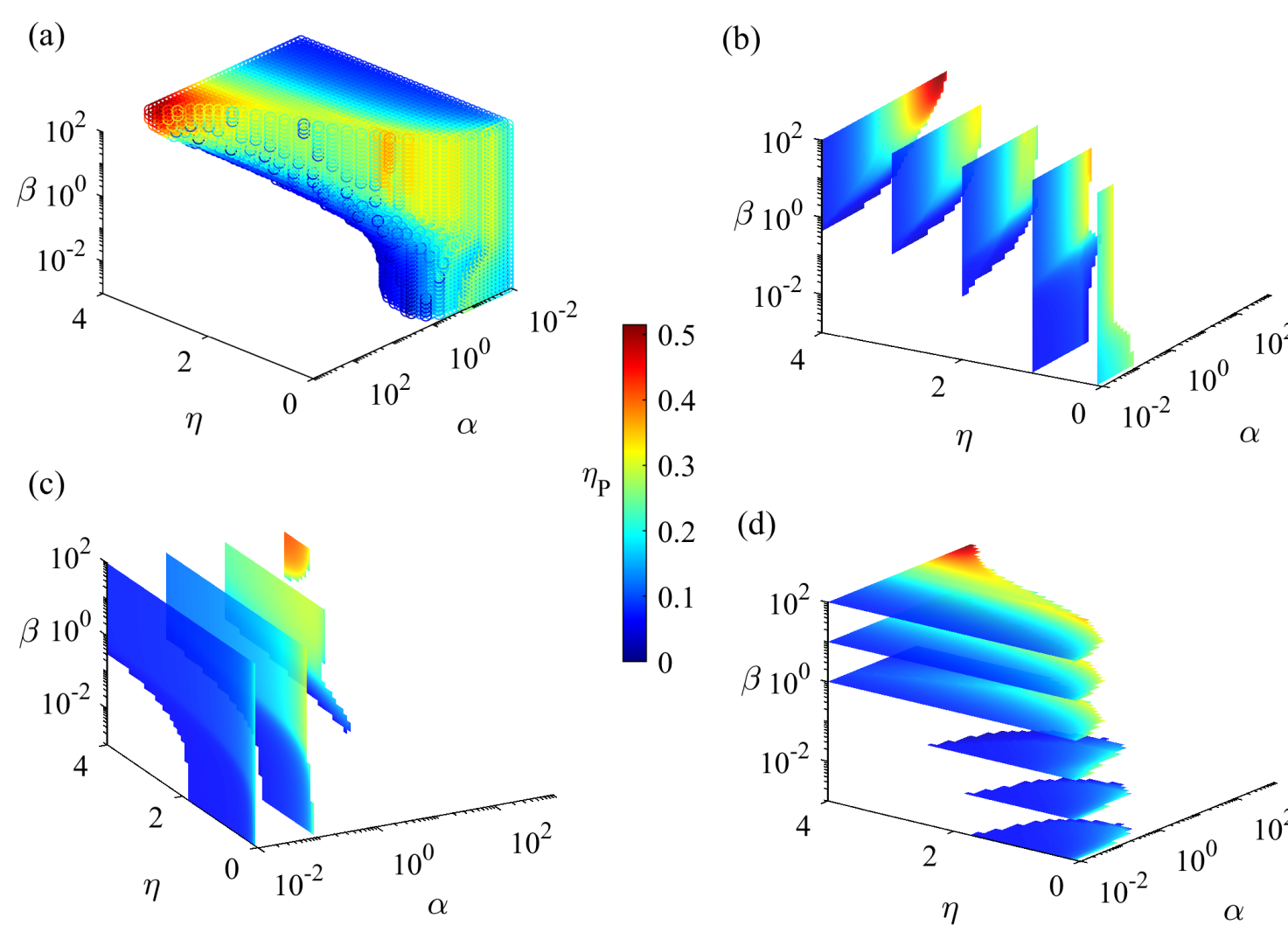

Figure 8: Heat shock response when aggregation is increased ten-fold: (a) Performance index plotted as a function of feedforward, sequestration, and degradation feedback strengths. Slice plots showing side and top views of the performance index are shown in (b), (c), and (d), respectively.

2. Robustness to variation in unfolded protein influx:

When the unfolded protein influx was reduced ten-fold, the overall proteotoxic burden experienced by the system was substantially lower. Consequently, activation of the HSR was weaker, resulting in reduced chaperone production (Fig. 9b). Since fewer unfolded proteins entered the folding cycle, the total number of folded proteins recovered was also lower (Fig. 9c). As a result, the benefit-to-cost ratio also decreased (Fig. 9d), reflecting the reduced number of proteins rescued per chaperone molecule.

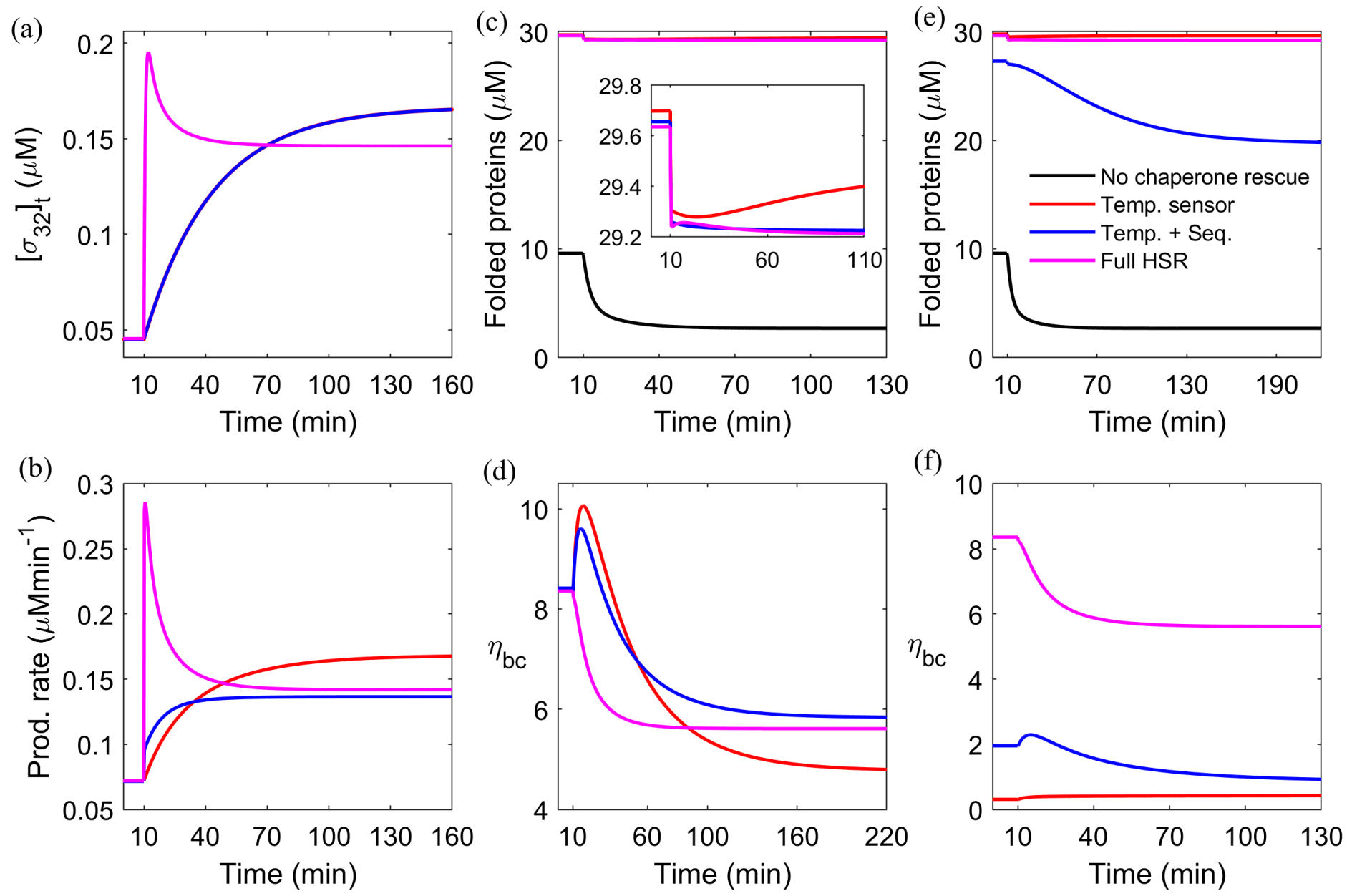

Figure 9: Heat shock response when unfolding flux is decreased ten-fold: (a) Concentration of $[\sigma_{32}]_t$, (b) production rate of chaperones, (c,e) folded proteins, and (d,f) benefit-to-cost are plotted for feedforward (red line), feedforward with sequestration (blue line) and full HSR with feedforward, sequestration, and degradation (magenta line). Heat shock is introduced at time 10 min. The black line in (b,c) corresponds to folded proteins in the absence of chaperones. For panels (a–d), pre–heat shock steady-state levels were matched across all modules. Subsequently, the translation rates were increased in the reduced modules to match the recovery dynamics of the full HSR, and the resulting behavior is shown in panels (e,f).

Despite these quantitative changes, the qualitative behavior remained robust. Fig. 10 shows paradoxical slowing at high feedforward strength (η_T). Likewise, Fig. 11 shows that increasing sequestration strength (α) retained its dual regulatory role by enhancing the fold change in chaperone production through σ_{32} sequestration while simultaneously reducing the adaptation time by facilitating σ_{32} degradation. In contrast to the experimentally motivated parameter set, the fold change increased monotonically over the explored range of α. This is likely because the reduced influx of unfolded proteins substantially reduces the proteotoxic burden, preventing sequestration from becoming sufficiently strong to limit σ_{32} availability and observe a decrease in fold change. This likely reflects a shift in nonmonotonic dynamics beyond the explored parameter range rather than a change in the underlying regulatory mechanism.

Fig. 12 confirms that the qualitative structure of the performance landscape is preserved despite quantitative changes in the optimal operating regime. Compared to the extended model of the main

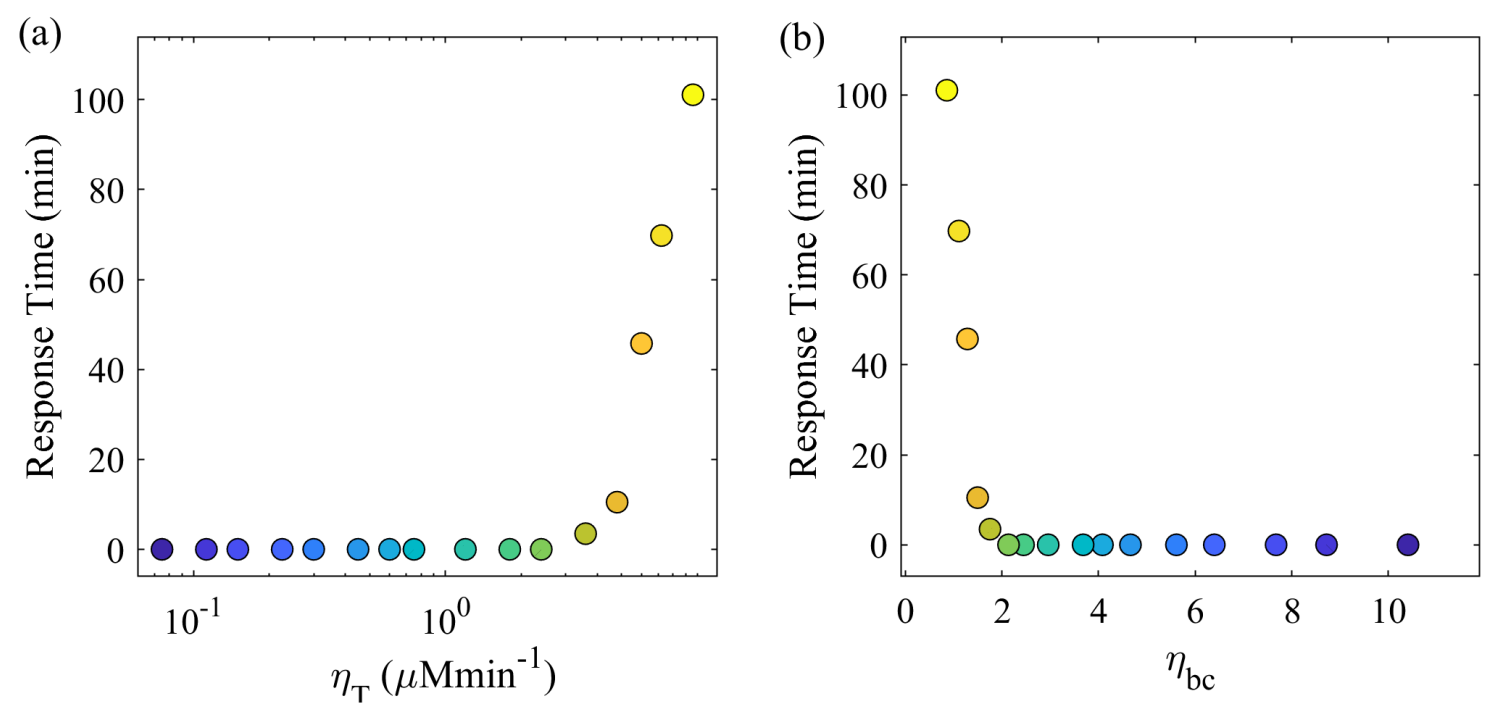

Figure 10: Heat shock response when unfolding flux is decreased ten-fold: Response time for varying feedforward strength (η_T values) is plotted against η_T and benefit-to-cost ratio in (a,b), respectively. The gradient from blue to yellow represents increasing σ_{32} sequestration with η_T, which also affects the timing of chaperone availability for misfolded proteins.

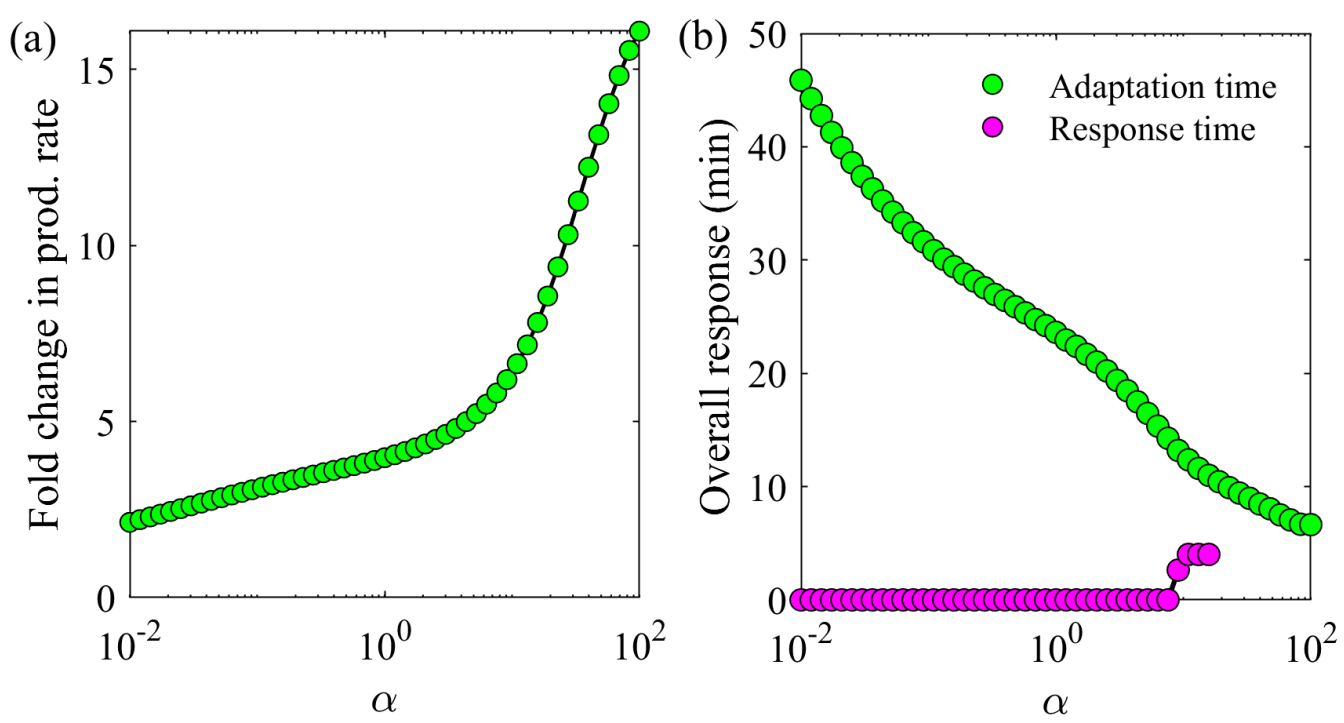

Figure 11: Heat shock response when unfolding flux is decreased ten-fold: (a) Fold change in the peak production rate relative to the pre-stress production, and (b) adaptation and response times are plotted for varying sequestration strength (α).

text, the high-performance region shifts toward a lower feedforward strength and the maximum attainable performance index is reduced. This is because a lower unfolded protein influx reduces the proteotoxic burden and, consequently, the number of proteins that require rescue. Under these conditions, stronger feedforward activation incurs a relatively larger regulatory cost without a proportional improvement in folded-protein recovery, resulting in lower benefit-to-cost ratios and reduced overall performance.

Conversely, increasing the unfolded protein influx by one order of magnitude imposed a substantially greater proteotoxic burden on the cell, resulting in stronger activation of the HSR. Consequently, both chaperone production (Fig. 13b) and the benefit-to-cost ratio (Fig. 13e) increased. As expected, the larger influx of unfolded proteins caused a more pronounced depletion of the folded-protein pool immediately after heat shock, followed by gradual recovery (Fig. 13c). Consistent with the extended model of the main text, Fig. 13 shows that the full HSR architecture continued to outperform the reduced regulatory architectures.

Figure 14 again demonstrates the nonmonotonic dependence of response time on feedforward strength (η_T). However, compared to the extended model of the main text, lower η_T values produce longer response delays. This is because the increased influx of unfolded protein, especially under stress, requires stronger σ_{32} activation to generate sufficient chaperone levels for efficient protein

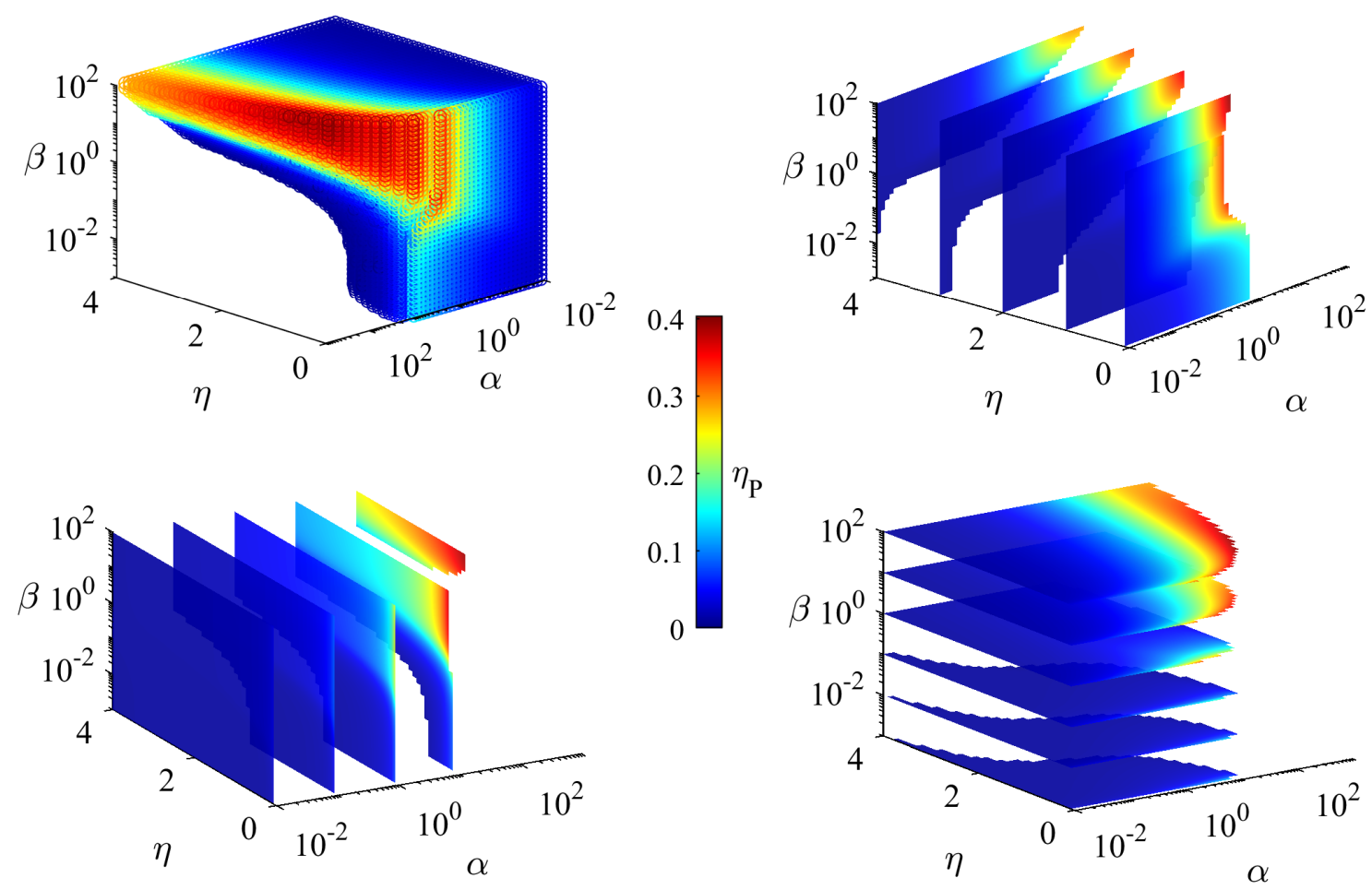

Figure 12: Heat shock response when unfolding flux is decreased ten-fold: (a) Performance index plotted as a function of feedforward, sequestration, and degradation feedback strengths. Slice plots showing side and top views of the performance index are shown in (b), (c), and (d), respectively.

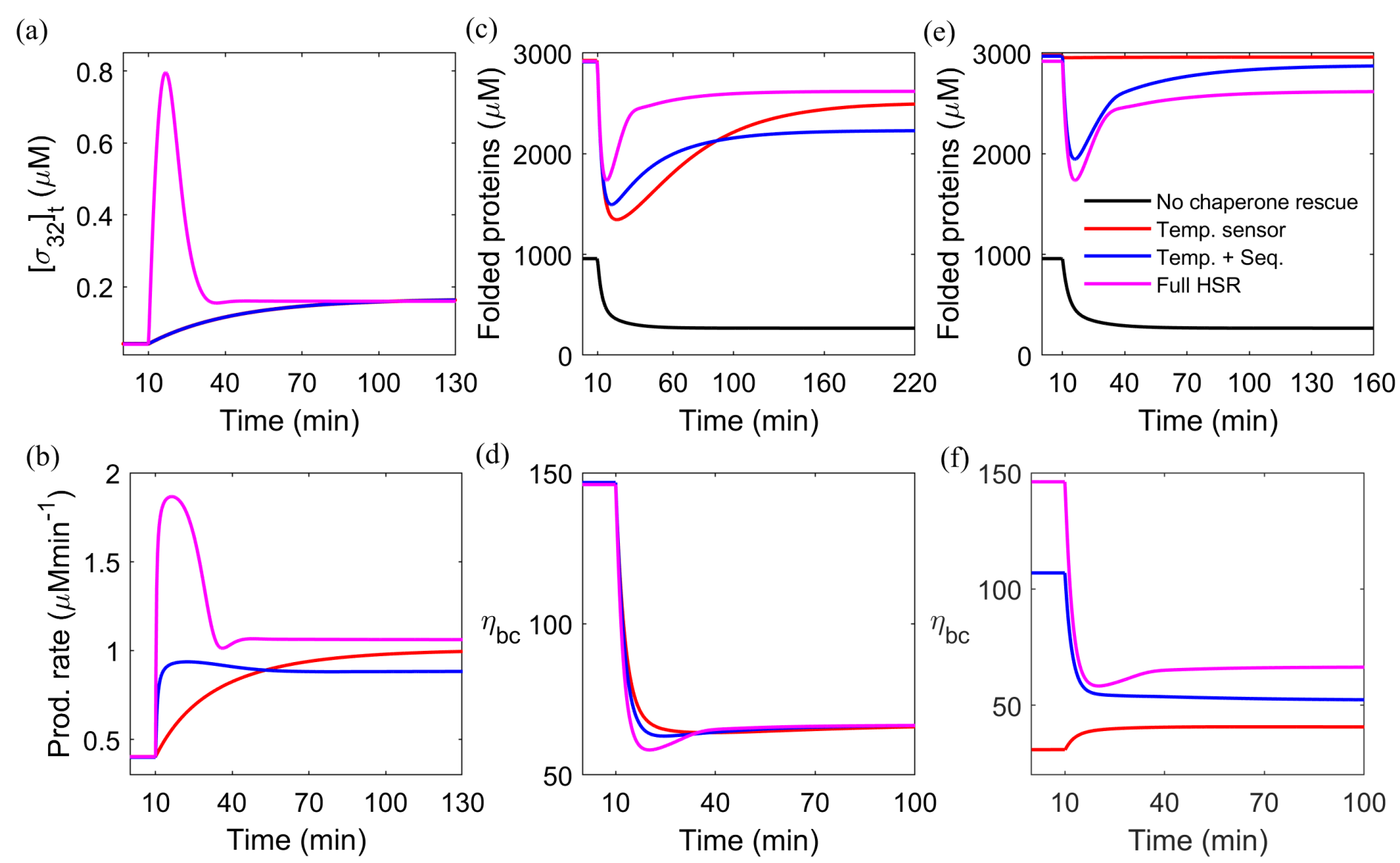

Figure 13: Heat shock response when unfolding flux is increased ten-fold: (a) Concentration of $[\sigma_{32}]_t$, (b) production rate of chaperones, (c,e) folded proteins, and (d,f) benefit-to-cost are plotted for feedforward (red line), feedforward with sequestration (blue line) and full HSR with feedforward, sequestration, and degradation (magenta line). Heat shock is introduced at time 10 min. The black line in (b,c) corresponds to folded proteins in the absence of chaperones. For panels (a–d), pre–heat shock steady-state levels were matched across all modules. Subsequently, the translation rates were increased in the reduced modules to match the recovery dynamics of the full HSR, and the resulting behavior is shown in panels (e,f).

recovery. Consequently, weak feedforward activation becomes inadequate under the increased proteotoxic burden. Nevertheless, the synergistic and trade-off regimes between response time and benefit-to-cost ratio are preserved (Fig. 14b).

Figure 15 shows that both the fold change in chaperone production and the adaptation time

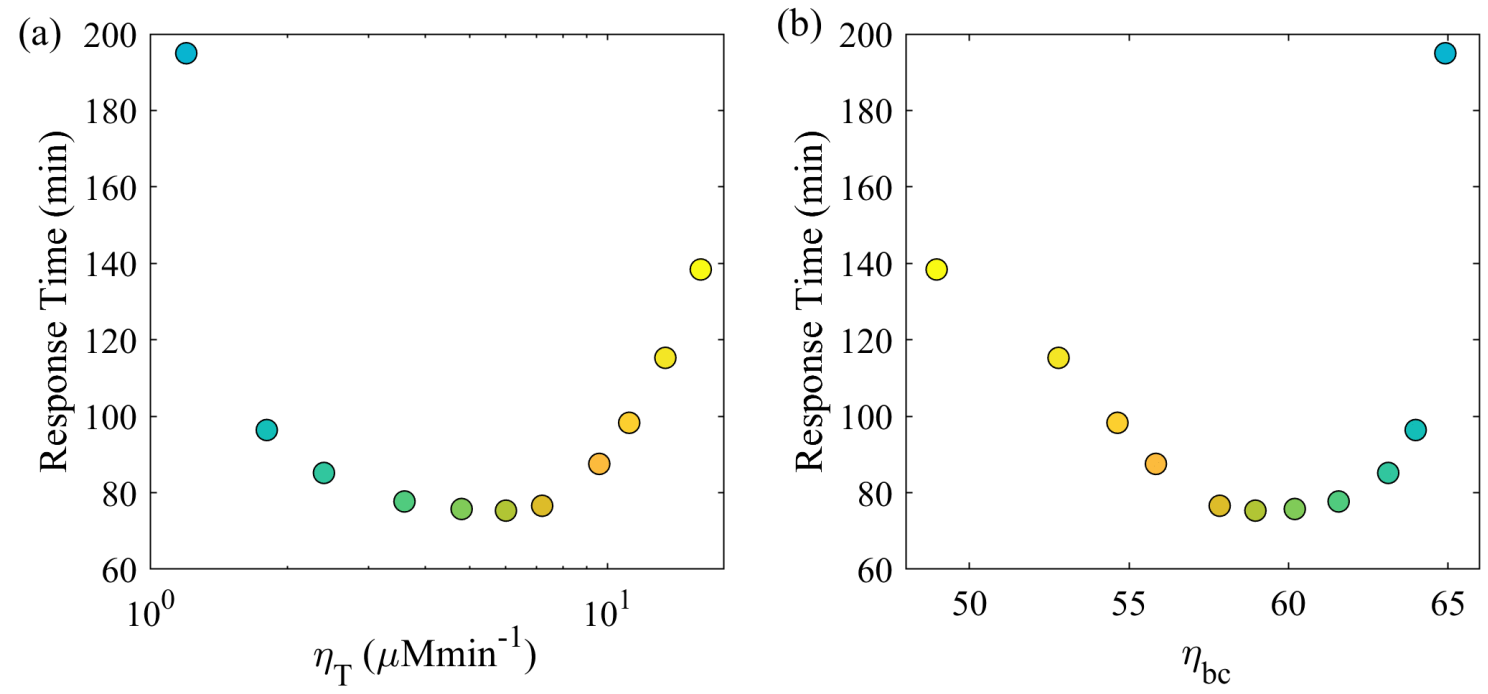

Figure 14: Heat shock response when unfolding flux is increased ten-fold: Response time for varying feedforward strength (η_T values) is plotted against η_T and benefit-to-cost ratio in (a,b), respectively. The gradient from blue to yellow represents increasing σ_{32} sequestration with η_T, which also affects the timing of chaperone availability for misfolded proteins.

retain their characteristic nonmonotonic dependence on sequestration strength (α). However, compared to the extended model with experimentally motivated rates, the overall response time is longer and becomes more sensitive to increasing sequestration strength. This is expected because the higher influx of unfolded protein imposes a greater demand for chaperone-assisted refolding. Consequently, stronger sequestration diverts a larger fraction of chaperones toward σ_{32} regulation, reducing their availability for refolding the expanded pool of misfolded proteins. As a result, recovery of 97% of the folded-protein pool becomes progressively more difficult at higher α.

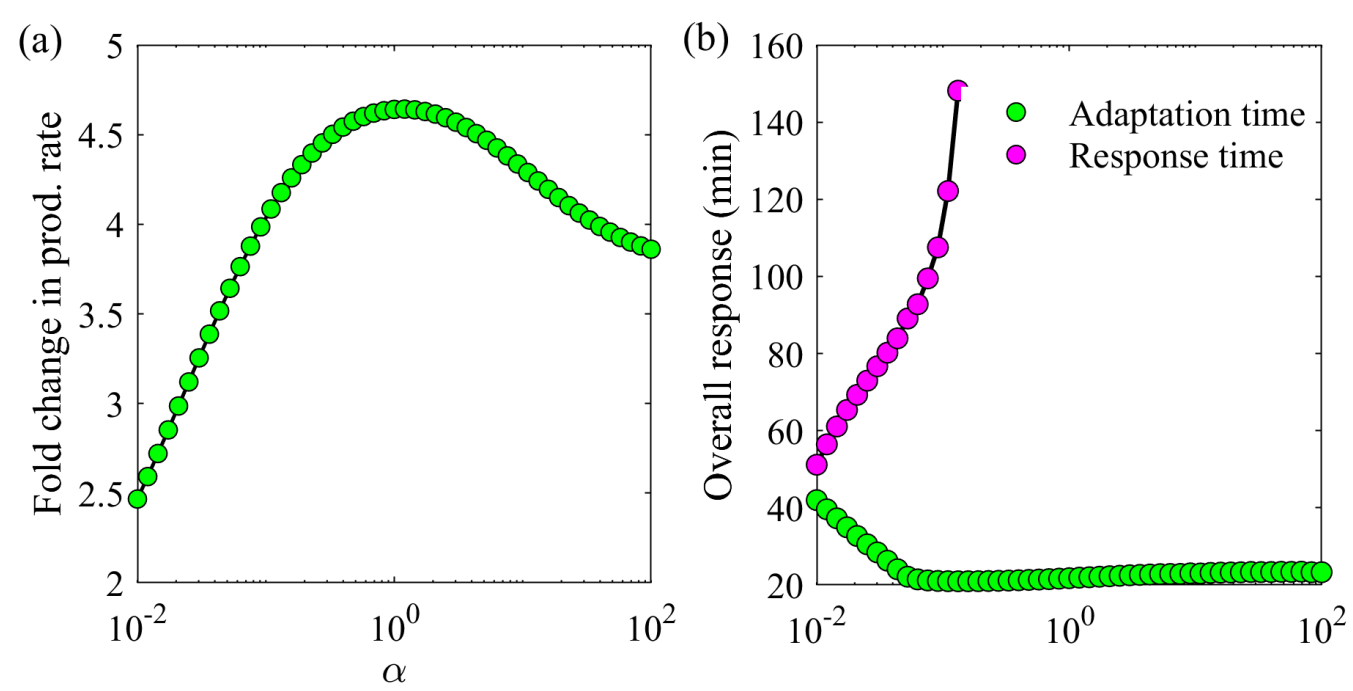

Figure 15: Heat shock response when unfolding flux is increased ten-fold: (a) Fold change in the peak production rate relative to the pre-stress production, and (b) adaptation and response times are plotted for varying sequestration strength (α).

Finally, Fig. 16 shows that the qualitative structure of the performance landscape remains preserved. The optimal operating regime continues to occupy the same region of parameter space, although it contracts and the white region (corresponding to less than 97% folded protein recovery) expands compared to the extended model of the main text. This is expected because of the higher proteotoxic burden, making near-complete recovery more difficult to achieve. Nevertheless, the high performance region increases, reflecting the greater benefit obtained when a larger pool of unfolded proteins is successfully rescued.

Overall, varying the unfolded protein influx also modulates the severity of proteotoxic stress and therefore quantitatively alters recovery dynamics, chaperone utilization, and the extent of the high-performance region. Nevertheless, the principal mechanistic conclusions remain unchanged.

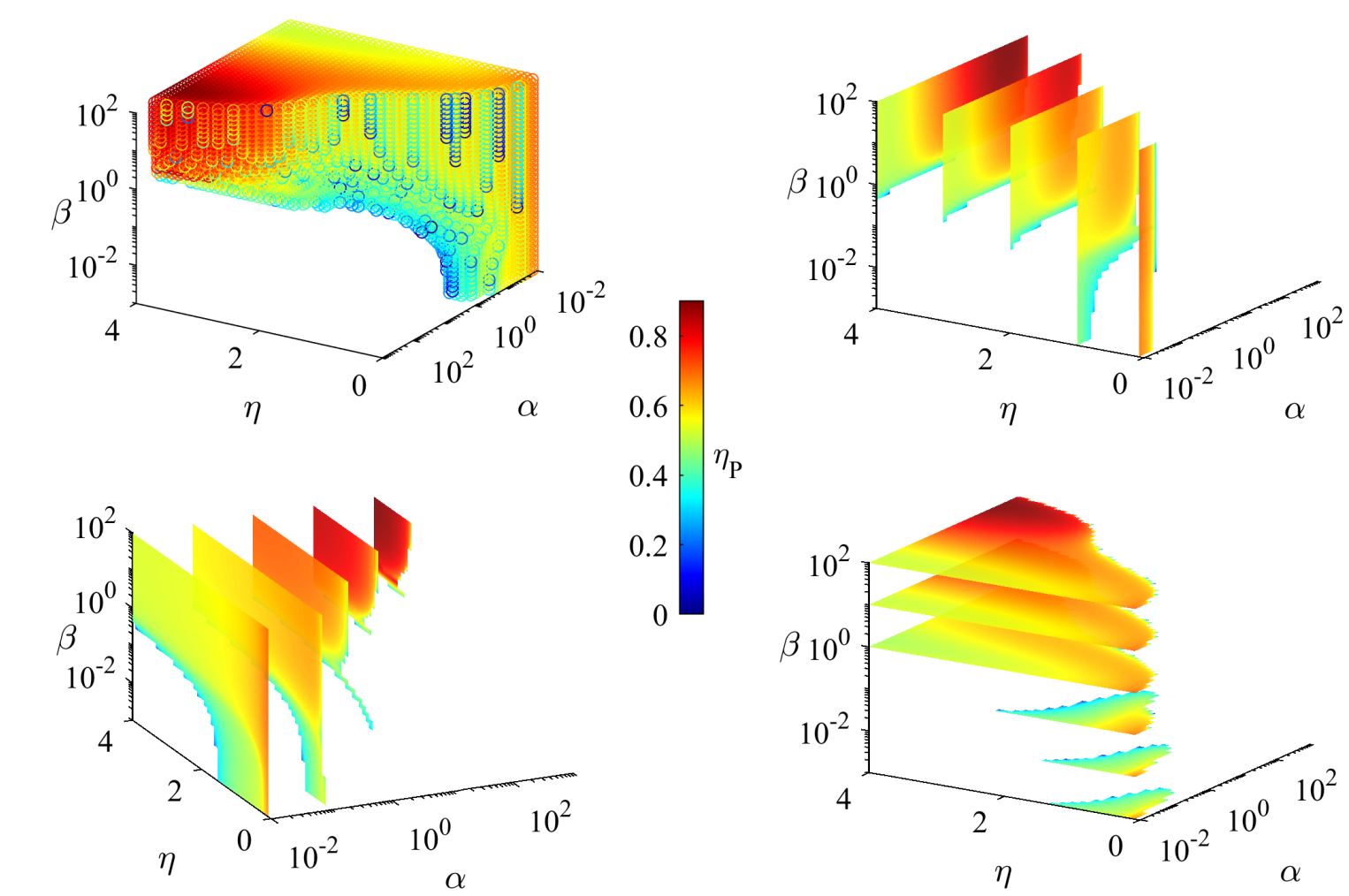

Figure 16: Heat shock response when unfolding flux is increased ten-fold: (a) Performance index plotted as a function of feedforward, sequestration, and degradation feedback strengths. Slice plots showing side and top views of the performance index are shown in (b), (c), and (d), respectively.

3. Robustness to variation in the protein folding rate:

When the protein folding rate was reduced by one order of magnitude, the stability of folded proteins decreased, making proteins substantially more susceptible to misfolding during heat shock. Consequently, in the absence of chaperones, the folded-protein pool rapidly collapsed and approached zero during stress (Fig. 17c). Because each protein now requires increased chaperone-assistance, the benefit-to-cost ratio decreased, reflecting fewer folded proteins rescued per chaperone molecule (Fig. 17d). Nevertheless, Fig. 17 shows that the full HSR architecture continued to outperform the reduced regulatory modules.

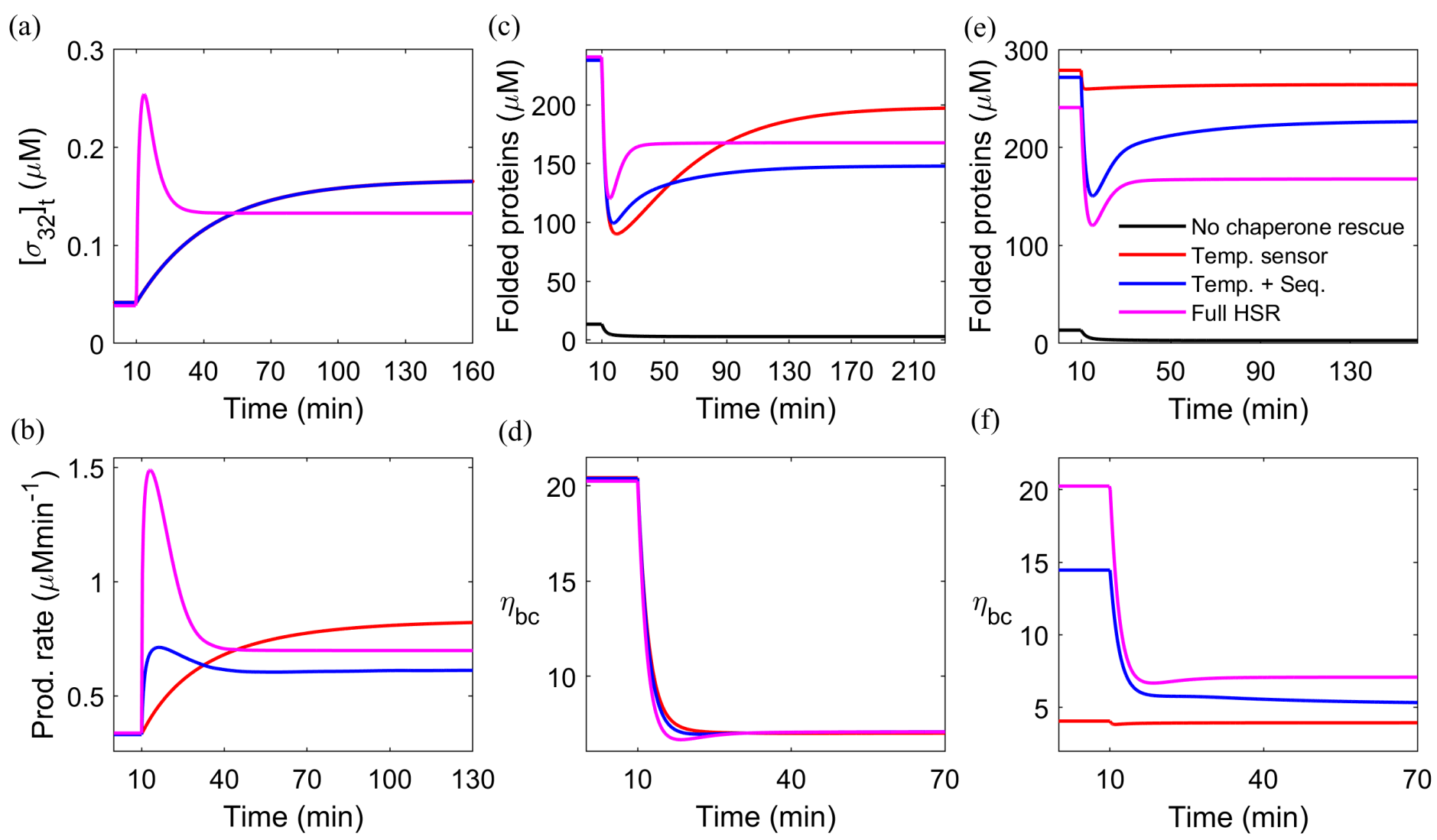

Figure 17: Heat shock response when protein folding rate is decreased ten-fold: (a) Concentration of $[\sigma_{32}]_t$, (b) production rate of chaperones, (c,e) folded proteins, and (d,f) benefit-to-cost are plotted for feedforward (red line), feedforward with sequestration (blue line) and full HSR with feedforward, sequestration, and degradation (magenta line). Heat shock is introduced at time 10 min. The black line in (b,c) corresponds to folded proteins in the absence of chaperones. For panels (a–d), pre–heat shock steady-state levels were matched across all modules. Subsequently, the translation rates were increased in the reduced modules to match the recovery dynamics of the full HSR, and the resulting behavior is shown in panels (e,f).

Fig. 18 shows nonmonotonic dependence of response time on feedforward strength (η_T), demonstrating that this behavior is robust even under substantially reduced folding capacity. Fig. 19 shows that fold change in chaperone production and adaptation time retained their nonmonotonic dependence on sequestration strength (α). However, none of the explored parameter combinations achieved the 97% recovery criterion because the reduced folding capacity made near-complete recovery difficult. Consequently, Fig. 20 shows that the high-performance region of the performance landscape became substantially smaller and the white region (failure to achieve 97% recovery) expanded.

Conversely, increasing the protein folding rate enhanced protein stability, making the folded-protein pool considerably more resistant to heat-induced misfolding. As a result, the folded-protein concentration exhibited only a small decrease following heat shock (Fig. 21c). The benefit-to-cost ratio increases after heat shock because the higher intrinsic folding stability before stress results in minimal demand for chaperone-assisted refolding. Heat shock increases protein misfolding, enabling

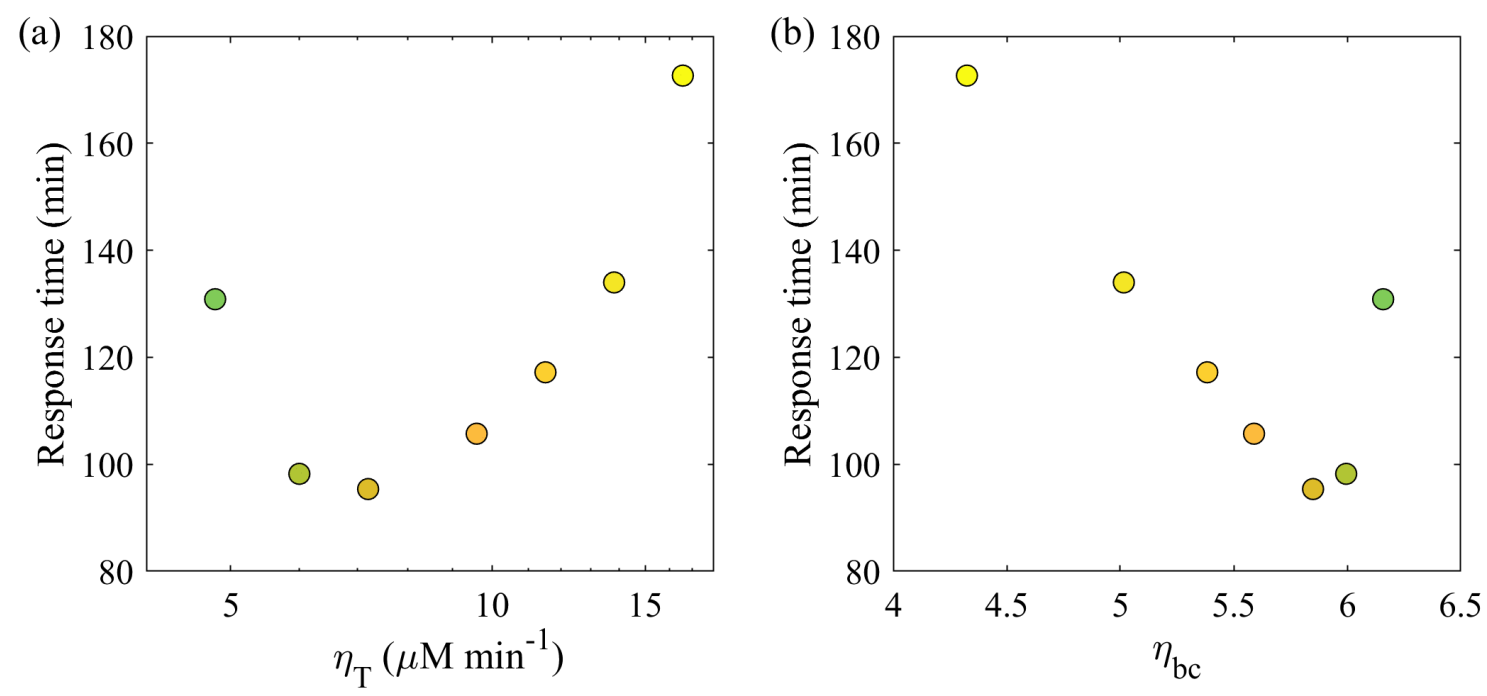

Figure 18: Heat shock response when protein folding rate is decreased ten-fold: Response time for varying feedforward strength (η_T values) is plotted against η_T and benefit-to-cost ratio in (a,b), respectively. The gradient from blue to yellow represents increasing σ_{32} sequestration with η_T, which also affects the timing of chaperone availability for misfolded proteins.

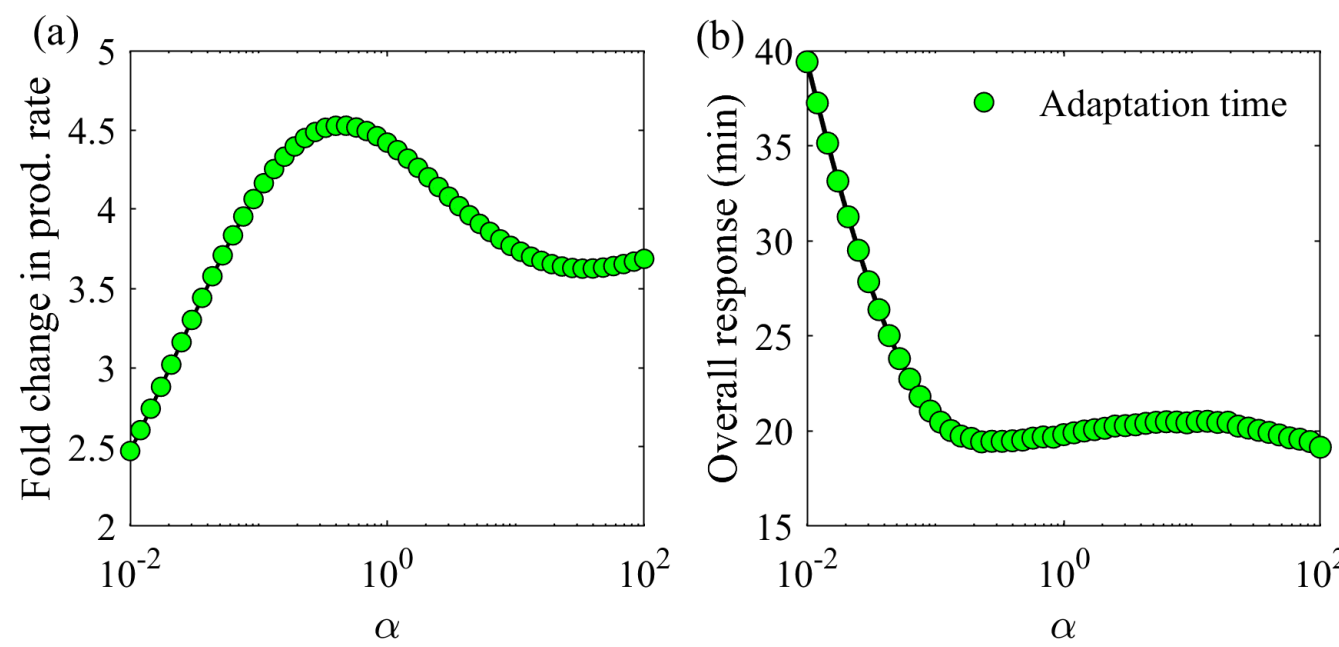

Figure 19: Heat shock response when protein folding rate is decreased ten-fold: (a) Fold change in the peak production rate relative to the pre-stress production, and (b) adaptation and response times are plotted for varying sequestration strength (α).

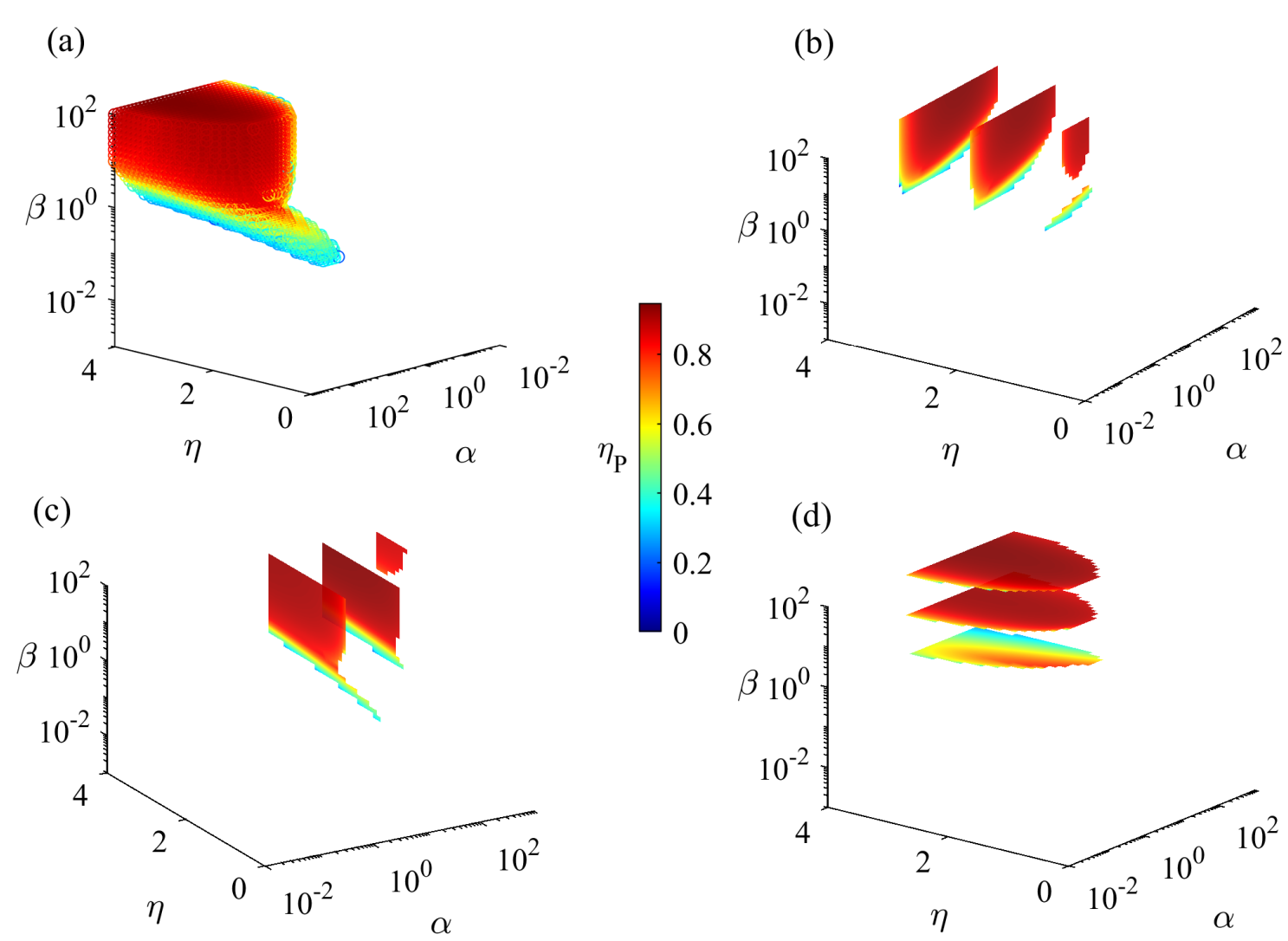

Figure 20: Heat shock response when protein folding rate is decreased ten-fold: (a) Performance index plotted as a function of feedforward, sequestration, and degradation feedback strengths. Slice plots showing side and top views of the performance index are shown in (b), (c), and (d), respectively.

chaperones to rescue a larger number of proteins, thereby increasing η_{bc} (Fig. 21d). Moreover, Fig. 21 shows that full HSR architecture continued to outperform the reduced modules.

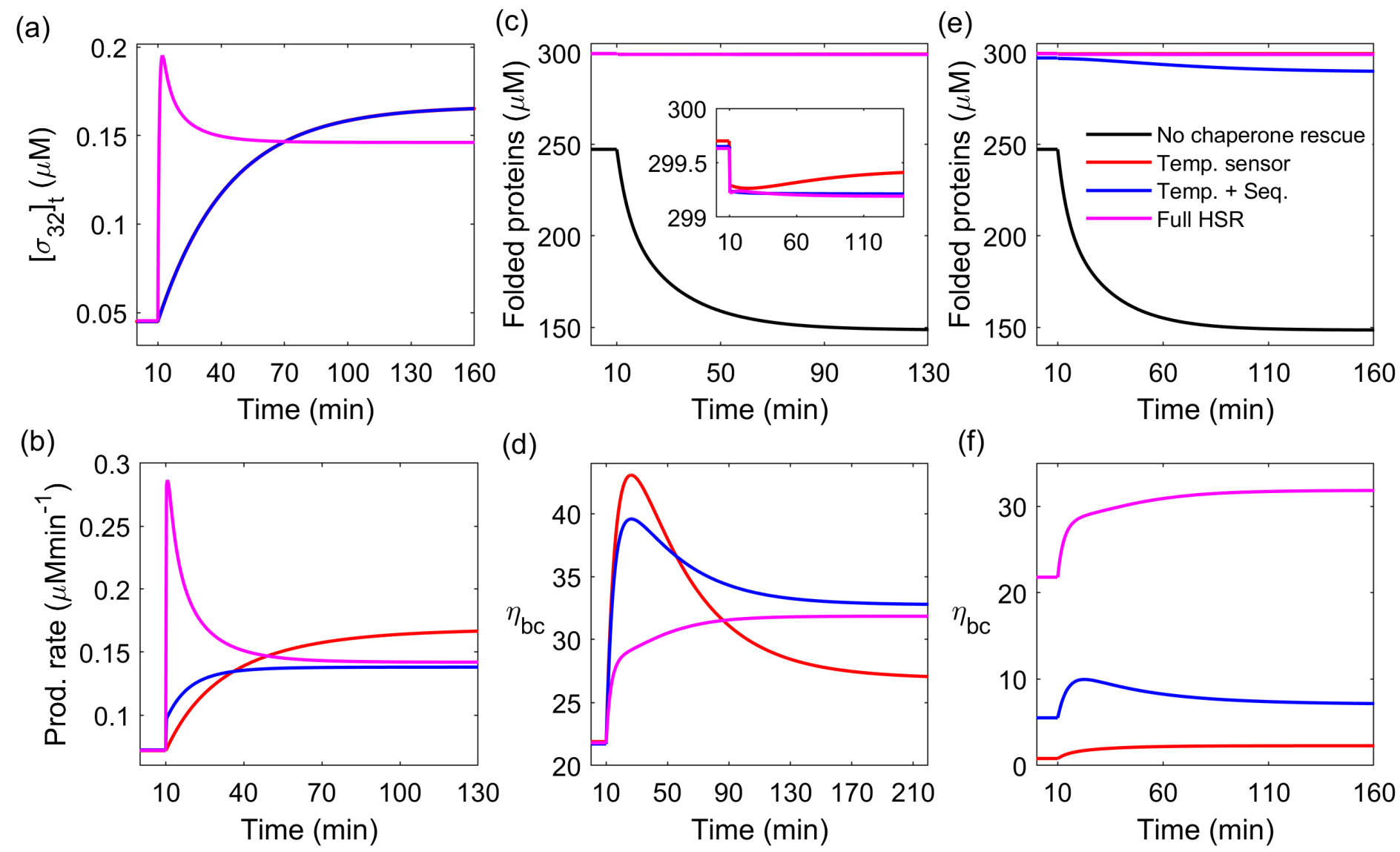

Figure 21: Heat shock response when protein folding rate is increased ten-fold: (a) Concentration of $[\sigma_{32}]_t$, (b) production rate of chaperones, (c,e) folded proteins, and (d,f) benefit-to-cost are plotted for feedforward (red line), feedforward with sequestration (blue line) and full HSR with feedforward, sequestration, and degradation (magenta line). Heat shock is introduced at time 10 min. The black line in (b,c) corresponds to folded proteins in the absence of chaperones. For panels (a–d), pre–heat shock steady-state levels were matched across all modules. Subsequently, the translation rates were increased in the reduced modules to match the recovery dynamics of the full HSR, and the resulting behavior is shown in panels (e,f).

Figure 22 again demonstrates the paradoxical slowing of recovery at higher feedforward strengths (η_T). However, compared to the extended model of the main text, recovery remains substantially faster over a broad range of η_T because the higher protein folding rate enables the folded-protein pool to recover more easily. Figure 23 shows that, in the explored range of sequestration strengths, the fold change increases monotonically while the adaptation time decreases monotonically. Moreover, the nonmonotonic behavior is not observed because the higher intrinsic folding stability makes the proteome more resilient to heat-induced damage. Consequently, substantially stronger sequestration is required before chaperone availability becomes sufficiently limiting to suppress chaperone production and reduce the fold change. As a result, the response time remains close to zero across most of the explored parameter range and increases only slightly at very high sequestration strengths (Fig. 23b).

Figure 24 shows that the qualitative structure of the performance landscape is preserved. Compared with the extended model in the main text, nearly the entire explored parameter space satisfies the 97% recovery criterion because the higher protein folding rate enables efficient recovery over a broad range of parameter combinations. However, the highest-performance region remains localized to high sequestration and degradation strengths with relatively low feedforward activation. This reflects the fact that, when proteins are intrinsically more stable, excessive feedforward activation increases chaperone production without providing a proportional improvement in folded-protein recovery, thereby reducing the benefit-to-cost ratio. Consistent with our previous

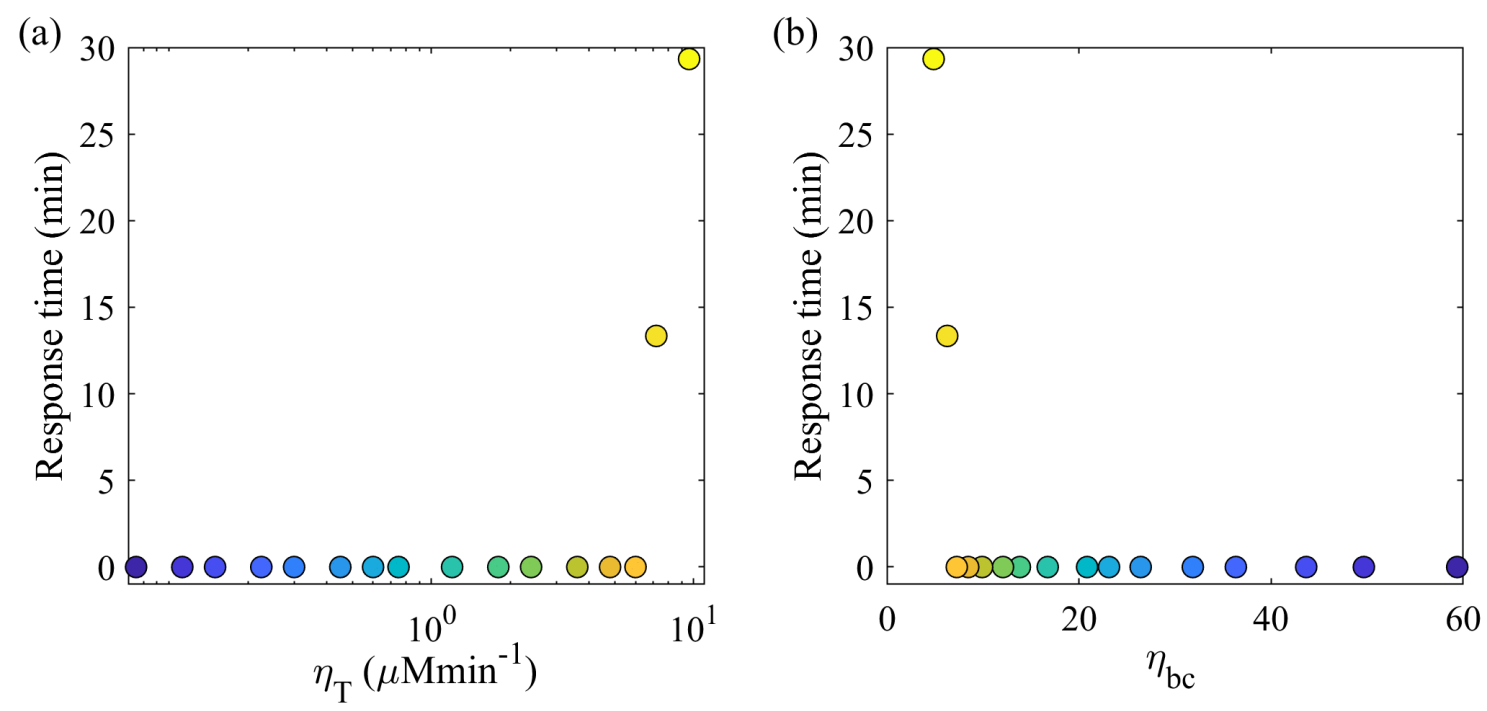

Figure 22: Heat shock response when protein folding rate is increased ten-fold: Response time for varying feedforward strength (η_T values) is plotted against η_T and benefit-to-cost ratio in (a,b), respectively. The gradient from blue to yellow represents increasing σ_{32} sequestration with η_T, which also affects the timing of chaperone availability for misfolded proteins.

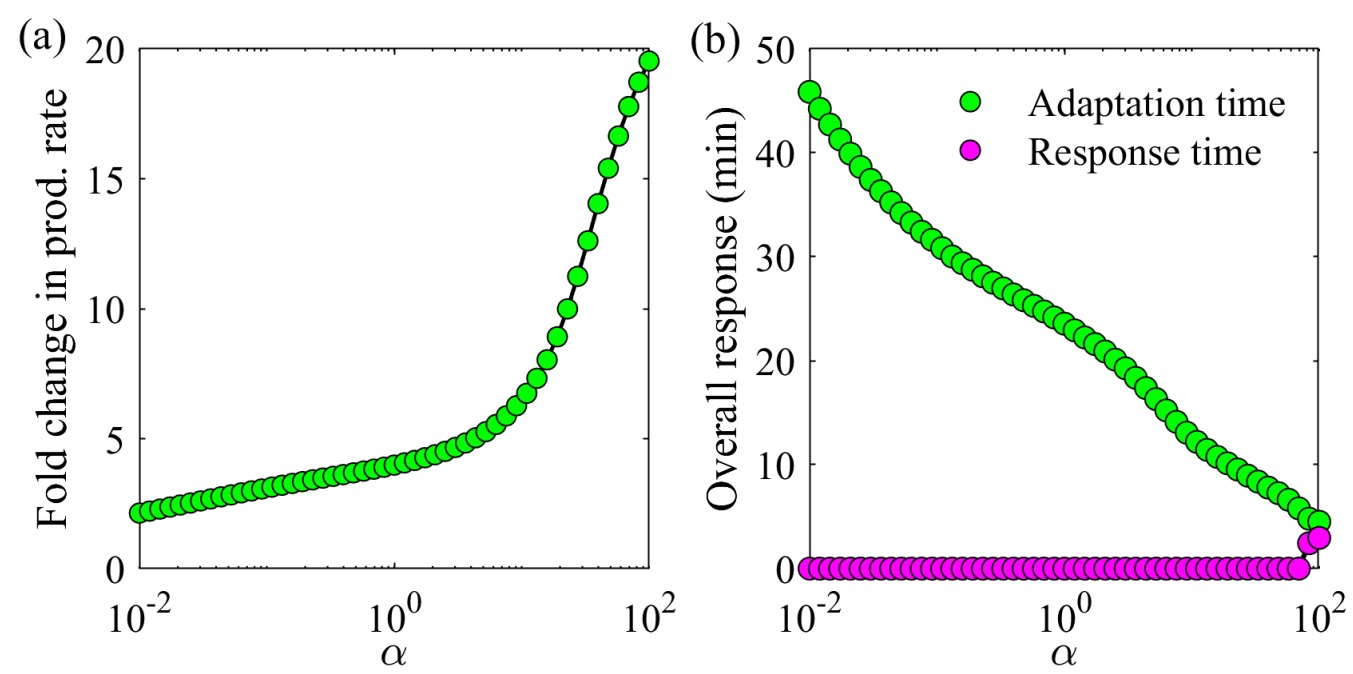

Figure 23: Heat shock response when protein folding rate is increased ten-fold: (a) Fold change in the peak production rate relative to the pre-stress production, and (b) adaptation and response times are plotted for varying sequestration strength (α).

results, degradation continues to act synergistically with feedforward activation and sequestration by clearing excess σ_{32}, releasing chaperones for protein folding, and preventing unnecessary chaperone overproduction.

Overall, varying the protein folding rate primarily modulates the intrinsic resilience of the proteome to heat-induced damage and therefore quantitatively alters recovery dynamics and the extent of the optimal operating regime.

Taken together, these robustness analyses demonstrate that the aggregation rate, unfolded-protein influx, and protein folding rate primarily modulate the severity of proteotoxic stress by altering the balance between protein damage and repair. Upon varying each of these rate parameters by an order of magnitude above and below their experimentally compatible values, the observed behavior closely replicated the qualitative conclusions of the main text. As expected, increasing the aggregation rate, increasing unfolded-protein influx, or reducing the protein folding rate made recovery more challenging, whereas the opposite perturbations facilitated recovery. A qualitatively different picture emerged only under two conditions: when the protein folding rate was increased ten-fold and when the unfolded protein influx was reduced ten-fold. In both the cases, the characteristic nonmonotonic dependence of response time on feedforward strength and fold change on sequestration strength was not observed within the explored parameter range. The main difference between these two and remaining other conditions is that the proteotoxic stress is substantially

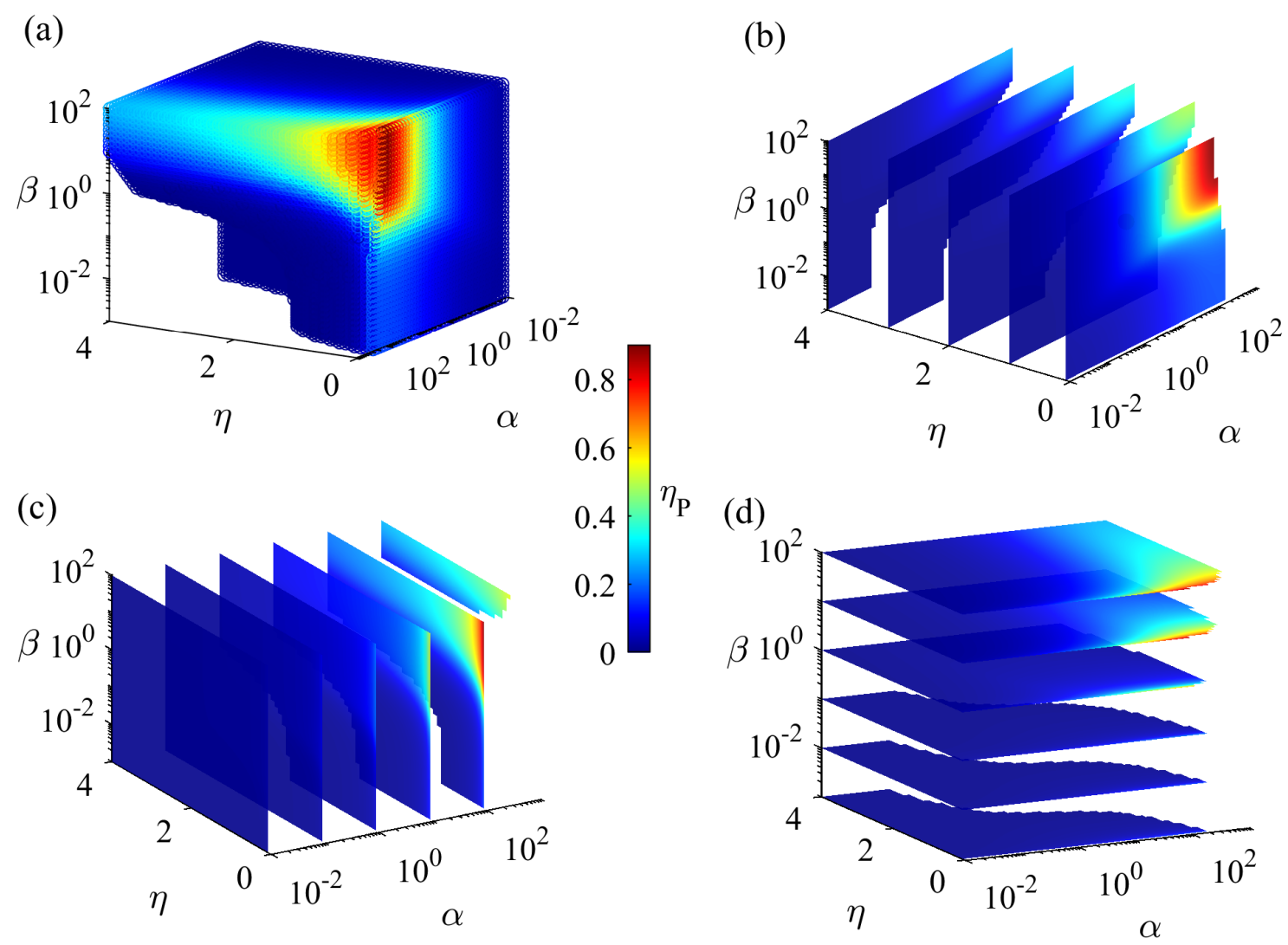

Figure 24: Heat shock response when protein folding rate is increased ten-fold: (a) Performance index plotted as a function of feedforward, sequestration, and degradation feedback strengths. Slice plots showing side and top views of the performance index are shown in (b), (c), and (d), respectively.

milder, with reduced misfolding and aggregation tendency. This shows that deviation only occurs when system is closer to normal conditions (without stress) where the chaperone burden is low. Despite the absence of nonmonotonic behavior in Figs. 10, 11, 22, and 23, the core conclusions remain intact. The trade-off between response speed and chaperone efficiency, the paradoxical slowing of recovery at high feedforward activation, the dual regulatory role of sequestration in simultaneously enhancing chaperone production and enabling timely shutdown, the synergistic contribution of degradation, and the emergence of a high-performance operating region, remain robust across the explored parameter ranges. These findings demonstrate that the main conclusions of this study arise from the interplay among the three feedback modules of the HSR rather than from any particular choice of aggregation, folding, or unfolding parameters.
